# Cell microparticles loaded with tumor antigen and resiquimod reprogram tumor-associated macrophages and promote stem-like CD8^+^ T cells to boost anti-PD-1 therapy

**DOI:** 10.1038/s41467-023-41438-9

**Published:** 2023-09-13

**Authors:** Xiaoqiong Zhang, Zhaohan Wei, Tuying Yong, Shiyu Li, Nana Bie, Jianye Li, Xin Li, Haojie Liu, Hang Xu, Yuchen Yan, Bixiang Zhang, Xiaoping Chen, Xiangliang Yang, Lu Gan

**Affiliations:** 1 National Engineering Research Center for Nanomedicine, College of Life Science and Technology, Huazhong University of Science and Technology, Wuhan, China; 2 Key Laboratory of Molecular Biophysics of the Ministry of Education, College of Life Science and Technology, Huazhong University of Science and Technology, Wuhan, China; 3 Hubei Key Laboratory of Bioinorganic Chemistry and Materia Medica, Huazhong University of Science and Technology, Wuhan, China; 4 Hubei Bioinformatics and Molecular Imaging Key Laboratory, Huazhong University of Science and Technology, Wuhan, China; 5 Hepatic Surgery Center, Tongji Hospital, Tongji Medical College, Huazhong University of Science and Technology, Wuhan, China

**Keywords:** Drug delivery, Cancer immunotherapy, Tumour immunology, Nanotechnology in cancer

## Abstract

The durable response rate to immune checkpoint blockade such as anti-programmed cell death-1 (PD-1) antibody remains relatively low in hepatocellular carcinoma (HCC), mainly depending on an immunosuppressive microenvironment with limited number of CD8^+^ T cells, especially stem-like CD8^+^ T cells, in tumor tissues. Here we develop engineered microparticles (MPs) derived from alpha-fetoprotein (AFP)-overexpressing macrophages to load resiquimod (R848@M2pep-MPs_AFP_) for enhanced anti-PD-1 therapy in HCC. R848@M2pep-MPs_AFP_ target and reprogram immunosuppressive M2-like tumor-associated macrophages (TAMs) into M1-like phenotype. Meanwhile, R848@M2pep-MPs_AFP_-reprogrammed TAMs act as antigen-presenting cells, not only presenting AFP antigen to activate CD8^+^ T cell-mediated antitumor immunity, but also providing an intra-tumoral niche to maintain and differentiate stem-like CD8^+^ T cells. Combination immunotherapy with anti-PD-1 antibody generates strong antitumor immune memory and induces abundant stem-like CD8^+^ T cell proliferation and differentiation to terminally exhausted CD8^+^ T cells for long-term immune surveillance in orthotopic and autochthonous HCC preclinical models in male mice. We also show that the R848-loaded engineered MPs derived from macrophages overexpressing a model antigen ovalbumin (OVA) can improve anti-PD-1 therapy in melanoma B16-OVA tumor-bearing mice. Our work presents a facile and generic strategy for personalized cancer immunotherapy to boost anti-PD-1 therapy.

## Abstract (web summary)

Tumor associated macrophages (TAMs) contribute to the immunosuppressive tumor microenvironment, including hepatocellular carcinoma (HCC). Here the authors show that macrophage-derived microparticles modified with a M2-like macrophage targeting peptide and loaded with the TLR7/8 agonist resiquimod reprogram TAMs from immunosuppressive to inflammatory, promoting anti-tumor immune responses in preclinical HCC models.

## Introduction

Hepatocellular carcinoma (HCC) is the sixth most prevalent malignancy and the third leading cause of cancer-related death worldwide^1^. Recently, immune checkpoint blockade (ICB), including anti-programmed cell death-1 (PD-1) antibody has achieved remarkable clinical success in a variety of cancers^2–4^. However, the objective response rates of anti-PD-1 therapy with pembrolizumab or nivolumab in patients with advanced HCC reached only about 18.3% or 15%, respectively^5,6^. Hence, it is highly desirable to develop efficient strategies to improve the therapeutic efficacy of anti-PD-1 antibody in HCC.

Anti-PD-1 antibody exerts anticancer activity by blocking the binding of PD-1 on T cells to PD-L1 on tumor cells to reinvigorate T cell-mediated antitumor immunity^2^, whose clinical effectiveness is highly associated with tumor immunosuppressive microenvironment and the extent of CD8^+^ T cell infiltration into tumors^7,8^. Recent works have shown that in addition to the magnitude of CD8^+^ T cells, the quality of CD8^+^ T cells is also an important determinant of the therapeutic effect of anti-PD-1 antibody^9,10^. A subset of PD-1^+^ exhausted T cells referred to as stem-like CD8^+^ T cells or progenitor exhausted T cells that express transcription factor T cell factor 1 (TCF1, encoded by *Tcf7*) exhibit expansion, self-renewal, persistence and differentiation capacities^11,12^. These CD8^+^PD-1^+^TCF-1^+^ T cells expand and differentiate into terminally exhausted CD8^+^ T cells (CD8^+^PD-1^+^TCF-1^-^ T cells) with increased cytotoxicity but are short-lived in the tumor microenvironment in response to anti-PD-1 antibody treatment^9,13,14^. The presence of stem-like CD8^+^ T cells correlates with the clinical benefit of anti-PD-1 therapy, and cancer patients who have a higher percentage of stem-like CD8^+^ T cells experience a longer duration of response to anti-PD-1 antibody^14^. Stem-like CD8^+^ T cells preferentially reside in the regions of aggregations of major histocompatibility complex II (MHC II)^+^ cells in tumors^15^. These antigen-presenting cell (APC) dense regions serve as an intra-tumoral niche for stem-like CD8^+^ T cells, which sustain the terminally exhausted CD8^+^ T cells to exert antitumor immune responses^15^. Thus, improving tumor immunosuppressive microenvironment and simultaneously expanding the population of CD8^+^ T cells, especially stem-like CD8^+^ T cells with an APC niche in tumor tissues, might be a viable strategy for enhancing the response to anti-PD-1 antibody in HCC therapy.

Tumor-associated macrophages (TAMs) with an M2-like phenotype (M2-like TAMs), one of the most abundant tumor-infiltrating immune cells, act as the main drivers for HCC development and progression^16^. M2-like TAMs serve to maintain a strong immunosuppressive microenvironment by expressing inhibitory immune checkpoints (such as PD-L1, PD-L2, B7-H4 and VISTA) and secreting cytokines (such as transforming growth factor-β and IL-10) to inhibit CD8^+^ T cell recruitment and activation as well as increase regulatory T (Treg) cells^17–19^. However, TAMs are highly plastic and can acquire M1-like phenotype in response to tumor microenvironment changes or therapeutic interventions^19,20^. M1-like TAMs exert antitumor activity by secreting proinflammatory cytokines, such as tumor necrosis factor-α (TNF-α) and IL-12^21,22^. In addition, unlike M2-like TAMs which highly express lysosomal cysteine protease to impede antigen cross-presentation and prevent CD8^+^ T cell activation^23^, M1-like TAMs as professional APCs play important roles in the induction of antitumor immunity, thereby serving as a bridge linking innate and adaptive immunity^24,25^. Especially, recent work has shown that antigen presentation by TAMs as a key factor correlates with immune resistance. In the resistant tumors, TAMs remain inactive and do not exert antigen-presenting activity^26^. In view of the high abundance of TAMs in tumor tissues and the relevance between antigen presentation by TAMs and immune resistance, reprogramming M2-like TAMs toward M1-like phenotype to ameliorate tumor immunosuppressive microenvironment, present tumor antigen to activate CD8^+^ T cells and create an APC niche suitable for stem-like CD8^+^ T cells might be a promising strategy for boosting anti-PD-1 therapy in HCC.

**Fig. 1:**
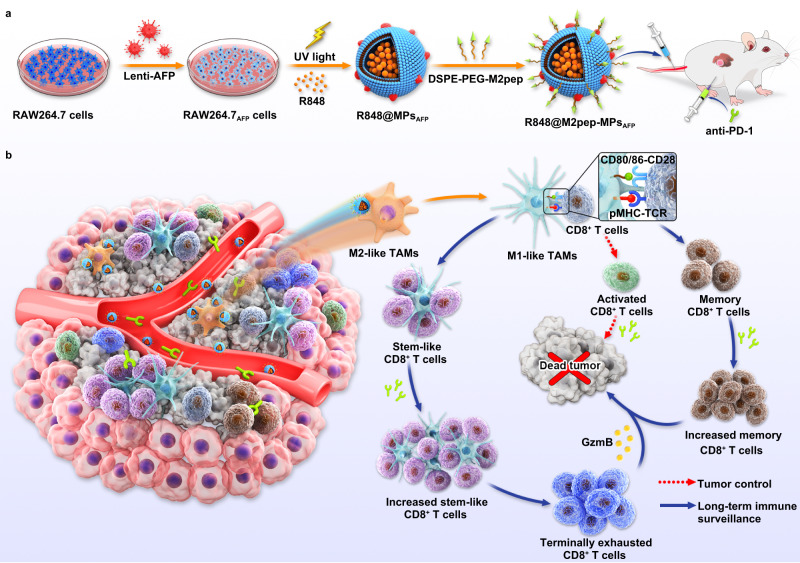
Schematic of R848@M2pep-MPs_AFP_ as an efficient therapeutic strategy to potentiate anti-PD-1 antibody therapy in HCC. **a** Schematic illustration of the preparation of R848@M2pep-MPs_AFP_. R848@M2pep-MPs_AFP_ were obtained by loading R848 to M2pep-conjugated MPs derived from AFP-overexpressing RAW264.7 cells by lentivirus transduction. **b** Schematic illustration of R848@M2pep-MPs_AFP_ to boost anti-PD-1 antibody therapy in HCC. R848@M2pep-MPs_AFP_ targeted and reprogrammed M2-like TAMs into M1-like phenotype, followed by presenting AFP antigen to activate antigen-specific CD8^+^ T cell for tumor control and promote stem-like CD8^+^ T cell proliferation and differentiation. Combination with anti-PD-1 antibody generated strong antitumor immune memory and induced abundant stem-like CD8^+^ T cell proliferation and differentiation to terminally exhausted CD8^+^ T cells for long-term immune surveillance.

Resiquimod (R848), a potent Toll-like receptor 7 and 8 (TLR7/8) agonist for skin lesion treatment^27^, shows antiviral and antitumor immune responses^28^. R848 was widely used to reprogram M2-like TAMs to M1-like phenotype via the TLR7 MyD88-dependent signaling pathway^28,29^. Meanwhile, R848 may function as an immune adjuvant by binding to TLR7/8 in APCs, resulting in the release of multiple immunomodulatory cytokines, such as IL-6, IL-12 and interferon α to activate APCs and T cells^30^. However, systemic R848 administration will induce cytokine release syndrome and systemic autoimmunity^31^. Thus, how to achieve the targeted delivery of R848 to M2-like TAMs for improved therapeutic effects remains crucial. Recently, Turco et al and Rodell et al constructed R848-loaded β-cyclodextrin nanoparticles (CDNP-R848) to reprogram M2-like TAMs to M1-like phenotype for improved tumor immunosuppressive microenvironment^32,33^, however, the long-term antitumor activity remains to be further improved. Cell microparticles (MPs), extracellular vesicles with a diameter of 100–1000 nm, are released from cells by direct budding from the plasma membrane^34–36^. Macrophage-derived MPs exhibit great potential as drug delivery vehicles due to their tumor-targeting ability, high biocompatibility and low immunogenicity^19,37,38^. Alpha-fetoprotein (AFP), an oncofetal antigen and liver cancer marker overexpressed in the majority of human HCC^39^, has been used as HCC vaccine antigen^40,41^. In this work, AFP-overexpressing macrophage-derived MPs (MPs_AFP_) which are modified with M2 macrophage-targeting peptide (M2pep), preferentially binding to M2-like macrophages than other leukocytes^42,43^, are used to load R848 (R848@M2pep-MPs_AFP_, Fig. 1a). R848@M2pep-MPs_AFP_ efficiently target and reprogram M2-like TAMs into M1-like phenotype to ameliorate tumor immunosuppressive microenvironment. Importantly, R848@M2pep-MPs_AFP_-reprogrammed M2-like TAMs process and present AFP antigen to CD8^+^ T cells with R848 as the immune adjuvant, resulting in promoted CD8^+^ T cell proliferation and activation as well as enhanced stem-like CD8^+^ T cells in tumor tissues. Meanwhile, R848@M2pep-MPs_AFP_- reprogrammed M2-like TAMs can provide an intra-tumoral niche to maintain and differentiate stem-like CD8^+^ T cells. In combination of these characteristics, R848@M2pep-MPs_AFP_ efficiently boost anti-PD-1 therapy in HCC, generating antitumor immune memory and inducing stronger stem-like CD8^+^ T cell proliferation and differentiation to achieve a long-term immune surveillance (Fig. 1b). Moreover, the constructed nanocomposites can realize personalized cancer immunotherapy in combination with anti-PD-1 antibody by integrating tumor-specific antigen to macrophage-derived MPs in addition to HCC therapy.

## Results

### Responsiveness of HCC to anti-PD-1 antibody is associated with TAMs and CD8^+^ T cells

**Fig. 2:**
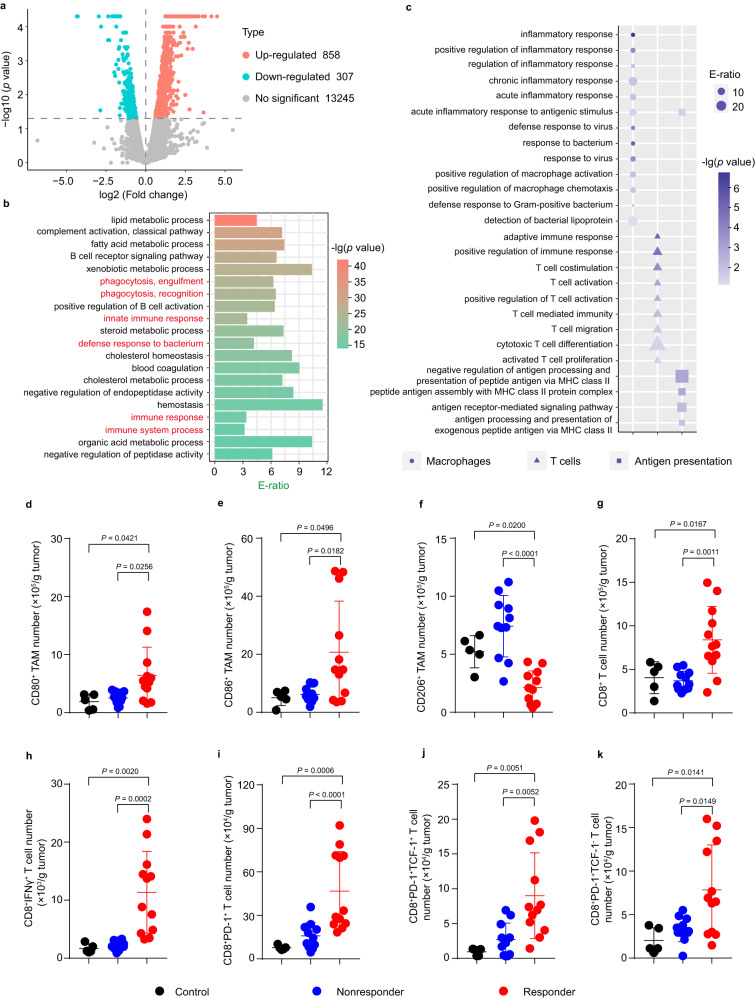
Relationship between the sensitivity of anti-PD-1 antibody therapy and tumor microenvironment. **a** Volcano plots showing differential gene expression in the tumor tissues of anti-PD-1 antibody-responsive and nonresponsive mice. (*n* = 3 mice per group; two-tailed Student’s *t*-test for comparison using Cuffdiff in the Cufflinks package). **b** Top 20 enrichment ratios (E-ratio) in GO term enrichment analysis of differentially expressed genes in tumor tissues of anti-PD-1 antibody-responsive and nonresponsive mice. (n = 3 mice per group; one-sided hypergeometric test). **c** GO enrichment analysis of the differentially expressed genes associated with macrophages, T cells and antigen presentation pathways in tumor tissues of anti-PD-1 antibody-responsive and nonresponsive mice. (*n* = 3 mice per group; one-sided hypergeometric test). **d**–**k** The numbers of CD80^+^ TAMs (**d**), CD86^+^ TAMs (**e**), CD206^+^ TAMs (**f**), CD8^+^ T (**g**), CD8^+^IFNγ^+^ T (**h**), CD8^+^PD-1^+^ T (**i**), CD8^+^PD-1^+^TCF-1^+^ T (**j**), and CD8^+^PD-1^+^TCF-1^-^ T (**k**) cells in tumor tissues of orthotopic Hepa1-6 tumor-bearing mice after intraperitoneal injection of PBS or anti-PD-1 antibody at the dosage of 5 mg kg^−1^ every four days for 5 times indicated in Supplementary Fig. 1a. Data are presented as means ± s.d. (*n* = 5 mice for control group, *n* = 11 mice for anti-PD-1 nonresponsive group, *n* = 12 mice for anti-PD-1 responsive group; one-way ANOVA followed by Tukey’s HSD post-hoc test). Source data are provided as a Source Data file.

Tumor microenvironment profoundly affects the therapeutic efficacy of ICB^8^. In view of the fact that inbred mouse strains bearing monoclonal cancer cell line-derived tumors respond in a dichotomous manner to ICB^44–46^, to gain insight into the mechanisms of ICB resistance in HCC, orthotopic Hepa1-6 tumor-bearing mice were intraperitoneally injected with PBS or anti-PD-1 antibody every four days for five times (Supplementary Fig. 1a) and then the tumors from the PBS-treated mice, anti-PD-1 antibody-responsive and nonresponsive mice which were selected according to tumor weight after treatment relative to PBS-treated group (Supplementary Fig. 1b, c) were used for analyzing the difference in gene expression using bulk RNA sequencing (RNA-seq). Gene Ontology (GO) term enrichment analysis showed that the expressions of some genes involved in phagocytosis, innate immune response, defense response to bacterium and immune response were significantly elevated in the anti-PD-1 antibody-responsive tumors compared with anti-PD-1 antibody-nonresponsive tumors (Fig. 2a, b). Since macrophages are effector cells of the innate immune system that phagocytose bacteria and other harmful micro-organisms^47^, GO enrichment analysis also confirmed that the expressions of genes associated with macrophage functions including inflammatory response, macrophage activation and chemotaxis, and pathogen response were significantly higher in the anti-PD-1 antibody-responsive tumors than those in anti-PD-1 antibody-nonresponsive tumors (Fig. 2c), suggesting that TAMs in the anti-PD-1 antibody-responsive mice might exhibit a stronger activation function. TAMs were more abundant than dendritic cells (DCs) in tumor tissues (Supplementary Fig. 2). Similar to DCs, macrophages are also crucial components of adaptive immune systems by presenting antigens to T cells^24,25^. In contrast to anti-PD-1 antibody-nonresponsive tumors, the expression of genes related to antigen presentation, T cell activation and T cell-mediated immunity was significantly enhanced in the anti-PD-1 antibody-responsive tumors (Fig. 2c). These results indicated that the activation and antigen presentation of TAMs as well as the immune response of T cells may be closely related to the responsiveness of HCC to anti-PD-1 antibody.

To further demonstrate the relationship between the responsiveness to anti-PD-1 antibody and TAMs or CD8^+^ T cells in orthotopic Hepa1-6 tumor-bearing mice, the numbers of immune cells in tumors of PBS- or anti-PD-1 antibody-treated mice were detected by flow cytometry. Significantly more CD80^+^ TAMs (Fig. 2d) and CD86^+^ TAMs (Fig. 2e), while less CD206^+^ TAMs (Fig. 2f) were detected in anti-PD-1-responsive mice compared with PBS-treated and anti-PD1-nonresponsive mice. Moreover, the numbers of CD8^+^ T cells (Fig. 2g) and CD8^+^IFNγ^+^ cells (Fig. 2h) were much higher in the anti-PD-1 antibody-responsive mice compared with other groups. Besides the number of tumor-infiltrating CD8^+^ T cells, the composition of the tumor-infiltrating CD8^+^ T cells can be an important determinant to respond to ICB^12^. Stem-like T cells having expansion, regeneration, and differentiation capacities remain responsive to ICB by acting as a precursor to generate terminally exhausted T cells with increased cytotoxicity^11,12^. Consistently, the numbers of PD-1^+^ exhausted CD8^+^ T cells (CD8^+^PD-1^+^ T cells, Fig. 2i), stem-like CD8^+^ T cells (CD8^+^PD-1^+^TCF-1^+^ T cell, Fig. 2j) and terminally exhausted CD8^+^ T cells (CD8^+^PD-1^+^TCF-1^-^ T cells, Fig. 2k) in tumor tissues were the highest in anti-PD-1-responsive mice compared with other groups. These results further confirmed that the responsiveness of HCC to anti-PD-1 therapy was associated with the status of TAMs and CD8^+^ T cells, suggesting that reprogramming of TAMs to enhance CD8^+^ T cell function might improve the sensitivity of HCC to anti-PD-1 antibody.

### Preparation and characterization of R848@M2pep-MPs_AFP_

**Fig. 3:**
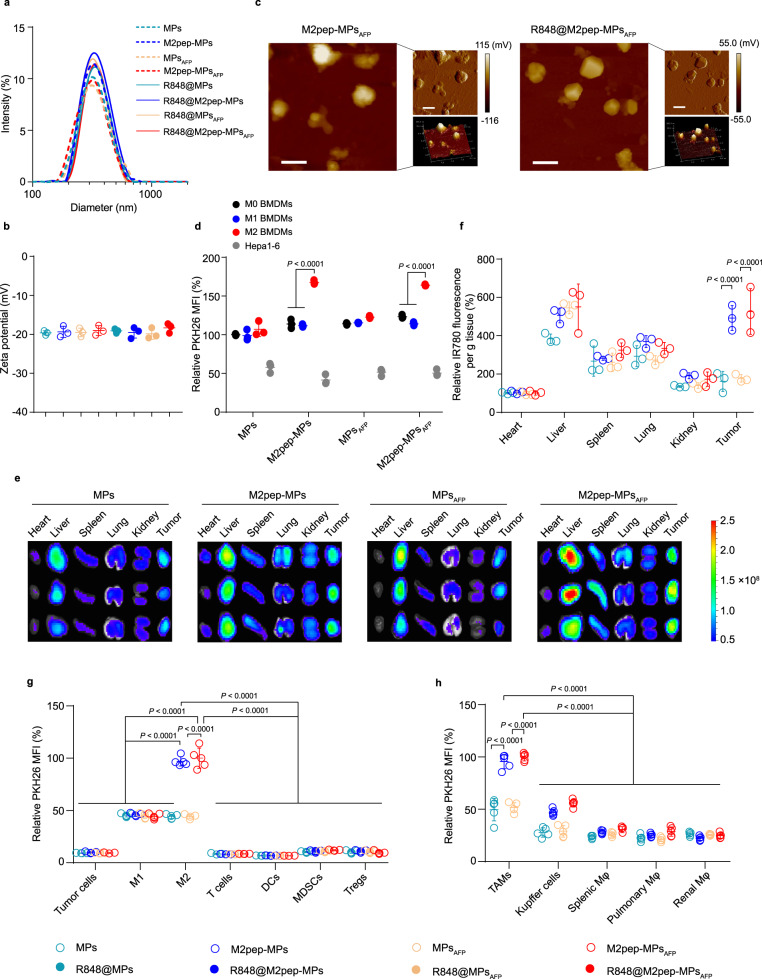
Characterization and M2-like TAM targeting of R848@M2pep-MPs_AFP_. **a,** **b** Hydrodynamic diameters (**a**) and zeta potentials (**b**) of MPs, M2pep-MPs, MPs_AFP_, M2pep-MPs_AFP_, R848@MPs, R848@M2pep-MPs, R848@MPs_AFP_ and R848@M2pep-MPs_AFP_ by DLS analysis. Data are presented as means ± sd (*n* = 3 independent samples). **c** Representative AFM height images (left), amplitude images (upper right) and three-dimensional morphology (lower right) of M2pep-MPs_AFP_ and R848@M2pep-MPs_AFP_. Scale bars: 400 nm. Images are representative of three independent samples. **d** Relative PKH26 mean fluorescence intensity (MFI) in BMDMs (M0 BMDMs), LPS- and IFNγ-stimulated BMDMs (M1-like BMDMs), IL-4-stimulated BMDMs (M2-like BMDMs) and Hepa1-6 cells after treatment with PKH26-labeled MPs, M2pep-MPs, MPs_AFP_ or M2pep-MPs_AFP_ at the concentration of 10 µg protein mL^−1^ for 4 h by flow cytometry. Data are presented as means ± sd (*n* = 3 biological independent samples; one-way ANOVA followed by Tukey’s HSD post-hoc test). **e**, **f** Ex vivo imaging (**e**) and relative fluorescence intensity (**f**) of IR780 in major organs and tumors of orthotopic Hepa1-6 tumor-bearing mice at 24 h after intravenous injection of IR780-labeled MPs, M2pep-MPs, MPs_AFP_ or M2pep-MPs_AFP_ at the dosage of 15 mg protein kg^−1^. Data are presented as means ± s.d. for (**f**). (*n* = 3 mice per group; one-way ANOVA followed by Tukey’s HSD post-hoc test). **g** Relative PKH26 MFI in tumor cells (CD45^-^ cells), M1-like TAMs (CD11b^+^F4/80^+^CD80^+^ cells), M2-like TAMs (CD11b^+^F4/80^+^CD206^+^ cells), T cells (CD45^+^CD3^+^ cells), DCs (CD45^+^F4/80^-^CD11c^+^ cells), MDSCs (CD45^+^CD11b^+^Gr1^+^ cells) and Tregs (CD45^+^CD3^+^CD4^+^CD25^+^FoxP3^+^ cells) in tumor tissues of orthotopic Hepa1-6 tumor-bearing mice at 24 h after intravenous injection of PKH26-labeled MPs, M2pep-MPs, MPs_AFP_ or M2pep-MPs_AFP_ at the dosage of 15 mg protein kg^−1^. Data are presented as means ± sd (*n* = 5 mice per group; two-way ANOVA followed by Bonferroni’s multiple comparisons post-test). **h** Relative PKH26 MFI in TAMs, liver Kupffer cells, splenic macrophages, pulmonary macrophages and renal macrophages (CD11b^+^F4/80^+^ cells) of orthotopic Hepa1-6 tumor-bearing mice at 24 h after treatment indicated in (**g**). Data are presented as means ± sd (*n* = 5 mice per group; two-way ANOVA followed by Bonferroni’s multiple comparisons post-test). Source data are provided as a Source Data file.

To construct engineered MPs derived from murine RAW264.7 macrophages for reprogramming TAMs, RAW264.7 cells were first infected with a lentivirus expressing murine AFP gene, an extensively used antigen marker for the diagnosis and treatment of HCC^39,41^, to obtain AFP-overexpressing macrophages (denoted as RAW264.7_AFP_ cells). RAW264.7 and RAW264.7_AFP_ cells were irradiated with ultraviolet for 1 h, and then treated with R848 (0.2 mg mL^−1^) for obtaining R848-packaging MPs and MPs_AFP_ (denoted as R848@MPs and R848@MPs_AFP_, respectively). The overexpression of AFP in RAW264.7_AFP_ cells (Supplementary Fig. 3a) and MPs_AFP_ (Supplementary Fig. 3b) was verified by western blotting. AFP overexpression significantly increased the ratio of CD80^+^ (Supplementary Fig. 4a), CD86^+^ (Supplementary Fig. 4b) and MHC II^+^ macrophages (Supplementary Fig. 4c), suggesting that AFP overexpression efficiently promoted macrophage maturation. Consistently, the expression of CD80 (Supplementary Fig. 4d), CD86 (Supplementary Fig. 4e) and MHC II (Supplementary Fig. 4f) was significantly enhanced in MPs_AFP_. It has been reported that M2pep (CYEQDPWGVKWWYK) possesses a high affinity for M2-like TAMs^42,43,48^. To achieve M2-like TAM targeting, R848@MPs and R848@MPs_AFP_ were incubated with DSPE-PEG-M2pep to obtain M2pep-modified R848@MPs and R848@MPs_AFP_ (denoted as R848@M2pep-MPs and R848@M2pep-MPs_AFP_, respectively). Confocal microscopic analysis clearly showed the colocalization of FITC-labeled M2pep and PKH26-labeled MPs (Supplementary Fig. 5), confirming that M2pep was successfully modified on MPs. Compared with MPs_AFP_ modified with mannose targeted to mannose receptor CD206/MRC1 highly expressed in M2-like macrophages (Man-MPs_AFP_)^19^, M2pep-MPs_AFP_ showed a stronger targeting capacity to M2-like macrophages (Supplementary Fig. 6). High-performance liquid chromatography (HPLC) analysis indicated that the R848 loading capacity for R848@M2pep-MPs_AFP_ was about 0.035 μg of R848 per μg protein. Dynamic light scattering (DLS) analysis revealed that R848@M2pep-MPs_AFP_ had a size of 352.9 nm (Fig. 3a) and zeta potential of -18.3 mV (Fig. 3b), similarly to R848@MPs, R848@MPs_AFP_ and R848@M2pep-MPs. Atomic force microscopy (AFM) showed that R848@M2pep-MPs_AFP_ were monodisperse and irregularly spherical (Fig. 3c). Moreover, R848@M2pep-MPs and R848@M2pep-MPs_AFP_ exhibited a pH-responsive sustained drug release (Supplementary Fig. 7). The size and zeta potential of R848@M2pep-MPs_AFP_ did not display obvious changes in phosphate-buffered saline (PBS) with or without 10% fetal bovine serum (FBS) after 7 days (Supplementary Fig. 8), indicating that R848@M2pep-MPs_AFP_ were relatively stable.

### M2pep-MPs_AFP_ efficiently target M2-like TAMs

To investigate the M2-like macrophage targeting ability of M2pep-modified MPs or MPs_AFP_, murine bone marrow-derived macrophages (M0 BMDMs), LPS- and IFNγ-stimulated BMDMs (M1-like BMDMs), IL-4-stimulated BMDMs (M2-like BMDMs) and murine Hepa1-6 hepatocarcinoma cells were treated with PKH26-labeled MPs, M2pep-MPs, MPs_AFP_ or M2pep-MPs_AFP_ for 4 h and then the mean fluorescence intensity (MFI) of intracellular PKH26 was evaluated by flow cytometry (Fig. 3d). M2-like BMDMs exhibited the strongest intracellular PKH26 fluorescence after treatment with M2pep-MPs or M2pep-MPs_AFP_ compared with other groups, suggesting a preferable M2-like macrophage targeting capacity of M2pep-MPs and M2pep-MPs_AFP_. Similar results were detected in IL-4-stimulated RAW264.7 cells (M2-like macrophages, Supplementary Fig. 9a). Pretreatment with free M2pep significantly reduced the intracellular content of M2pep-MPs and M2pep-MPs_AFP_ in M2-like macrophages (Supplementary Fig. 9b), demonstrating that the M2-like macrophage targeting capacity of M2pep-MPs and M2pep-MPs_AFP_ was mediated by M2pep. Intracellular tracing analysis of Rhodamine B (a model drug)-loaded M2pep-MPs_AFP_ (RhB@M2pep-MPs_AFP_) revealed that drug and M2pep-MPs_AFP_ were internalized into M2-like macrophages simultaneously and then distributed in lysosomes, followed by drug release with time (Supplementary Fig. 10).

The M2-like TAM targeting ability of M2pep-MPs and M2pep-MPs_AFP_ was further confirmed in vivo. Orthotopic Hepa1-6 tumor-bearing mice were intravenously injected with IR780-labeled MPs, M2pep-MPs, MPs_AFP_ or M2pep-MPs_AFP_. Whole-animal fluorescence imaging showed that more IR780 fluorescence was detected in tumor-bearing liver tissues of M2pep-MPs- and M2pep-MPs_AFP_-treated mice at 24 h after injection (Supplementary Fig. 11). Moreover, more M2pep-MPs and M2pep-MPs_AFP_ were accumulated in the tumors than MPs and MPs_AFP_ by ex vivo IR780 fluorescence analysis (Fig. 3e, f), suggesting that M2pep conjugation efficiently enhanced the tumor accumulation of MPs and MPs_AFP_. To further investigate whether M2pep-MPs and M2pep-MPs_AFP_ could be efficiently internalized by M2-like TAMs after reaching tumor tissues, PKH26-labeled MPs, M2pep-MPs, MPs_AFP_ or M2pep-MPs_AFP_ were intravenously injected into orthotopic Hepa1-6 tumor-bearing mice. At 24 h after treatment, the tumor tissues were digested into single cell suspensions and the PKH26 fluorescence intensities in different cells, including tumor cells, M1- and M2-like TAMs, T cells, DCs, myeloid-derived suppressor cells (MDSCs) and Tregs, were detected by flow cytometry (Fig. 3g). Compared with other cells, M1- and M2-like TAMs captured more MPs, M2pep-MPs, MPs_AFP_ or M2pep-MPs_AFP_. However, significantly more M2pep-MPs and M2pep-MPs_AFP_ were accumulated in M2-like TAMs than MPs and MPs_AFP_, confirming specific targeting of M2pep conjugation to M2-like TAMs. In addition, more M2pep-MPs and M2pep-MPs_AFP_ were enriched in TAMs than those in Kupffer cells, splenic macrophages, pulmonary macrophages and renal macrophages (Fig. 3h), excluding the targeting accumulation of M2pep-MPs_AFP_ in other tissue-resident macrophages.

### R848@M2pep-MPs_AFP_ efficiently reprogram M2-like macrophages and activate CD8^+^ T cells

To investigate the reprogramming of M2-like macrophages into M1-like phenotype by R848@M2pep-MPs_AFP_, IL-4-stimulated RAW264.7 cells were treated with PBS, MPs, M2pep-MPs, MPs_AFP_, M2pep-MPs_AFP_, free R848, R848@MPs, R848@M2pep-MPs, R848@MPs_AFP_ or R848@M2pep-MPs_AFP_ for 24 h, and the expressions of M1- and M2-related markers were detected by flow cytometry and reverse transcription-quantitative polymerase chain reaction (RT-qPCR). Compared with PBS, MPs, M2pep-MPs and free R848, R848@MPs obviously enhanced the protein expressions of CD80 (Supplementary Fig. 12a), CD86 (Supplementary Fig. 12b) and MHC II (Supplementary Fig. 12c), and the mRNA expressions of *CD80* (Supplementary Fig. 13a), *CD86* (Supplementary Fig. 13b), *TNF-α* (Supplementary Fig. 13c) and *iNOS* (Supplementary Fig. 13d) (M1-related markers), while decreased the protein expressions of CD206 (Supplementary Fig. 12d), and mRNA expressions of *Mgl1* (Supplementary Fig. 13e) and* Mrc1* (Supplementary Fig. 13f) (M2-related markers), thus corroborating the M2-like macrophage reprogramming capacity of R848@MPs. However, M2pep modification and AFP overexpression in MPs further enhanced the R848@MPs-induced M2-like macrophage reprogramming (Supplementary Fig. 12a–d and Supplementary Fig. 13a–f), which might be due to the M2pep-promoted internalization by M2-like macrophage and AFP-stimulated M1-like macrophage activation. R848@M2pep-MPs_AFP_ exhibited the strongest reprogramming activity compared with other groups (Supplementary Fig. 12a–d and Supplementary Fig. 13a–f). The excellent M2-like macrophage reprogramming capacity of R848@M2pep-MPs_AFP_ was further confirmed in IL-4-stimulated murine BMDMs (Supplementary Fig. 14a–d). The classically activated M1-like macrophages exert antitumor effects by secreting reactive nitrogen and oxygen species and proinflammatory cytokines. Consistently, the supernatants of R848@M2pep-MPs_AFP_-treated M2-like macrophages exhibited the strongest cytotoxicity against Hepa1-6 cells than those of MPs-, M2pep-MPs-, MPs_AFP_-, M2pep-MPs_AFP_-, free R848-, R848@MPs-, R848@M2pep-MPs- or R848@MPs_AFP_-treated group (Supplementary Fig. 15a). However, Etanercept (Etan), an inhibitor of TNF-α significantly reduced the cytotoxicity of the supernatants of R848@M2pep-MPs_AFP_-treated M2-like macrophages against Hepa1-6 cells (Supplementary Fig. 15b), suggesting that TNF-α secreted by R848@M2pep-MPs_AFP_-reprogrammed M2-like macrophages might be responsible for their strong cytotoxicity. Meanwhile, R848@M2pep-MPs_AFP_-reprogrammed M2-like macrophages displayed stronger phagocytosis of Hepa1-6 cells (Supplementary Fig. 16). The R848@M2pep-MPs_AFP_-induced reprogramming of M2-like macrophages was irrelevant to the lentivirus transfection, as no significant differences in the M2-like macrophage reprogramming capacity (Supplementary Fig. 17a–c) and the corresponding cytotoxicity of the supernatants of the reprogrammed M2-like macrophages (Supplementary Fig. 17d) were detected in the R848@M2pep-MPs- and R848-loaded M2pep-conjugated MPs derived from RAW264.7 cells stably transfected with empty vector (denoted as R848@M2pep-MPs_EV_)-treated groups.

M1-like macrophages as the classic APCs can present antigens to activate CD8^+^ T cells^23,25^. To investigate the AFP antigen presentation capacity of R848@M2pep-MPs_AFP_-educated M2-like macrophages, the proliferation and activation of CD8^+^ T cells induced by R848@M2pep-MPs_AFP_-reprogrammed M2-like RAW264.7 cells were determined (Supplementary Fig. 18a). Compared with MPs-, M2pep-MPs-, MPs_AFP_-, M2pep-MPs_AFP_-, free R848-, R848@MPs-, R848@M2pep-MPs- or R848@MPs_AFP_-treated group, R848@M2pep-MPs_AFP_-treated M2-like macrophages significantly enhanced the percentages of proliferated CD8^+^ T cells (Supplementary Fig. 18b), and activated CD8^+^ T cells expressing IFNγ (Supplementary Fig. 18c) and Granzyme B (GzmB, Supplementary Fig. 18d). Meanwhile, the activated CD8^+^ T cells by R848@M2pep-MPs_AFP_-reprogrammed M2-like macrophages possessed the strongest cytotoxicity against murine hepatocellular carcinoma Hepa1-6 cells which expressed AFP antigen in an effector/target ratio-dependent manner (Supplementary Fig. 18e–g). However, no significant difference was detected in the cytotoxicity of CD8^+^ T cells activated by R848@M2pep-MPs- and R848@M2pep-MPs_AFP_-reprogrammed M2-like macrophages against murine melanoma B16 cells overexpressing ovalbumin (OVA, B16-OVA cells) (Supplementary Fig. 18h–j). These results revealed that R848@M2pep-MPs_AFP_-reprogrammed M2-like macrophages, as APCs, may efficiently process and present AFP to promote the proliferation and activation of CD8^+^ T cells, thereby resulting in specific killing of AFP-expressing Hepa1-6 cells.

**Fig. 4:**
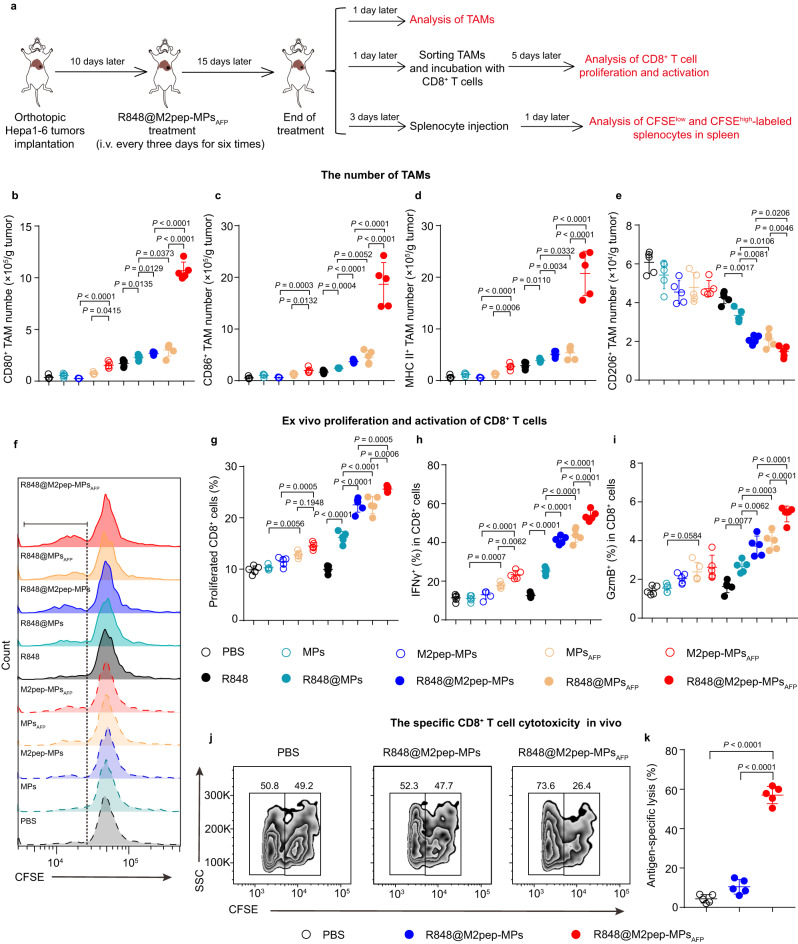
Efficiently reprogramming M2-like TAMs and activating CD8^+^ T cells by R848@M2pep-MPs_AFP_ in vivo. **a** Schematic schedule for reprogramming M2-like TAMs and activating CD8^+^ T cells by R848@M2pep-MPs_AFP_ in orthotopic Hepa1-6 tumor-bearing mice. **b**–**e** The numbers of CD80^+^ (**b**), CD86^+^ (**c**), MHC II^+^ (**d**), and CD206^+^ (**e**) TAMs in tumor tissues of orthotopic Hepa1-6 tumor-bearing mice after intravenous injection of PBS, MPs, M2pep-MPs, MPs_AFP_, M2pep-MPs_AFP_, R848, R848@MPs, R848@M2pep-MPs, R848@MPs_AFP_ or R848@M2pep-MPs_AFP_ at the R848 dosage of 0.5 mg kg^−1^ every three days for six times as indicated in (**a**). Data are presented as means ± s.d. (n = 5 mice per group; one-way ANOVA followed by Tukey’s HSD post-hoc test). **f**–**i** Representative flow plots (**f**) and percentages of proliferated CD8^+^ T cells (**g**), IFNγ^+^ (**h**), and GzmB^+^ (**i**) cells in CD8^+^ T cells at 5 days after co-culture with TAMs isolated from tumor tissues of orthotopic Hepa1-6 tumor-bearing mice which were intravenously injected with PBS, MPs, M2pep-MPs, MPs_AFP_, M2pep-MPs_AFP_, R848, R848@MPs, R848@M2pep-MPs, R848@MPs_AFP_ or R848@M2pep-MPs_AFP_ at the R848 dosage of 0.5 mg kg^−1^ every three days for six times as indicated in (**a**) by flow cytometry. Data are presented as means ± s.d. for (**g**–**i**) (*n* = 5 mice per group; one-way ANOVA followed by Tukey’s HSD post-hoc test). **j**, **k** Representative flow plots (**j**) and ratios of antigen-specific lysis (**k**) in splenocytes of orthotopic Hepa1-6 tumor-bearing mice at 24 h after intravenous injection of PBS, R848@M2pep-MPs or R848@M2pep-MPs_AFP_ at the R848 dosage of 0.5 mg kg^−1^ every three days for six times, followed by intravenous injection of the mixtures of CFSE^low^ OVA_257-264_-loaded and CFSE^high^ AFP_212_-loaded splenocytes at the ratio of 1:1 as indicated in (**a**) by flow cytometry. Data are presented as means ± s.d. for (**k**). (n = 5 mice per group; one-way ANOVA followed by Tukey’s HSD post-hoc test). Panels (**f**, **j**) show representative results of five independent samples. Source data are provided as a Source Data file.

The reprogramming of M2-like TAMs by R848@M2pep-MPs_AFP_ and the subsequent activation of CD8^+^ T cells were further confirmed in vivo (Fig. 4a). Orthotopic Hepa1-6 tumor-bearing mice were intravenously injected with PBS, MPs, M2pep-MPs, MPs_AFP_, M2pep-MPs_AFP_, R848, R848@MPs, R848@M2pep-MPs, R848@MPs_AFP_ or R848@M2pep-MPs_AFP_ every three days for six times. Flow cytometric analysis showed that R848@M2pep-MPs_AFP_ significantly increased the numbers of M1-like TAMs including CD80^+^ TAMs (Fig. 4b), CD86^+^ TAMs (Fig. 4c) and MHC II^+^ TAMs (Fig. 4d), while decreasing the numbers of M2-like TAMs, such as CD206^+^ TAMs (Fig. 4e) in tumor tissues, validating that R848@M2pep-MPs_AFP_ efficiently reprogrammed M2-like TAMs into M1-like phenotype. Furthermore, when TAMs isolated from tumor tissues of the above treated orthotopic Hepa1-6 tumor-bearing mice were co-cultured with the CD8^+^ T cells isolated from the spleens of health C57BL/6 mice for 5 days, the percentages of proliferated CD8^+^ T cells (Fig. 4f, g), and activated CD8^+^IFNγ^+^ T cells (Fig. 4h) and CD8^+^GzmB^+^ T cells (Fig. 4i) in R848@M2pep-MPs_AFP_-treated group were drastically higher than other groups. Moreover, an antigen-specific in vivo killing assay using carboxyfluorescein succinimidyl ester (CFSE)-labeled AFP_212_- or OVA_257-264_-preincubated splenocytes showed that R848@M2pep-MPs induced limited antigen-specific killing in mice (Fig. 4j, k). However, R848@M2pep-MPs_AFP_ showed 57.0% antigen-specific killing in vivo (Fig. 4j, k). These results further confirmed that R848@M2pep-MPs_AFP_ efficiently reprogrammed M2-like TAMs into M1-like phenotype, presenting AFP antigen to activate CD8^+^ T cell to specifically kill target cells highly expressing AFP antigen in vivo. Here, we noticed that R848@M2pep-MPs_AFP_ induced a more significant increase in MHC II expression in macrophages in vivo (Fig. 4d) compared with the in vitro results (Supplementary Fig. 12c), which might be because that besides the efficient R848@M2pep-MPs_AFP_-induced reprogramming of M2-like TAMs, the complex tumor microenvironment, such as the R848@M2pep-MPs_AFP_-promoted IFNγ expression of CD8^+^ T cells might also contribute to the increased numbers of MHCII^+^ TAMs in tumor tissues^49^.

### R848@M2pep-MPs_AFP_ significantly inhibit the growth of HCC and ameliorate tumor immune microenvironment

**Fig. 5:**
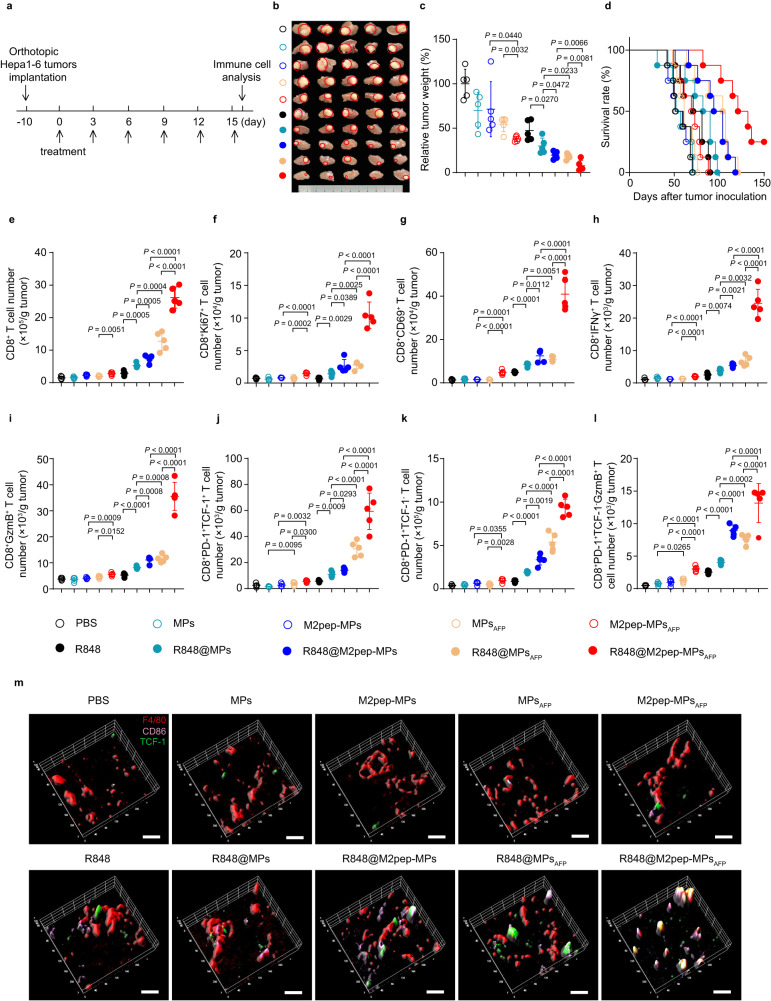
Potent antitumor activity and improved antitumor immunity of R848@M2pep-MPs_AFP_ in orthotopic Hepa1-6 tumor-bearing mice. **a** Schematic schedule for the antitumor experiment of R848@M2pep-MPs_AFP_ in orthotopic Hepa1-6 tumor-bearing mice. **b**, **c** Tumor images (**b**) and tumor weight (**c**) of orthotopic Hepa1-6 tumor-bearing mice at 16 days after intravenous injection of PBS, MPs, M2pep-MPs, MPs_AFP_, M2pep-MPs_AFP_, R848, R848@MPs, R848@M2pep-MPs, R848@MPs_AFP_ or R848@M2pep-MPs_AFP_ at the R848 dosage of 0.5 mg kg^−1^ every three days for six times. Data are presented as means ± s.d. for (**c**). (n = 5 mice per group; one-way ANOVA followed by Tukey’s HSD post-hoc test). **d** Kaplan–Meier survival plot of orthotopic Hepa1-6 tumor-bearing mice after treatment indicated in (**a**). (n = 8 mice per group). **e**–**l** The numbers of CD8^+^ T cells (gated as CD45^+^CD3^+^CD8^+^, **e**), CD8^+^Ki67^+^ T cells (gated as CD45^+^CD3^+^CD8^+^Ki67^+^, **f**), CD8^+^CD69^+^ T cells (gated as CD45^+^CD3^+^CD8^+^CD69^+^, **g**), CD8^+^IFNγ^+^ T cells (gated as CD45^+^CD3^+^CD8^+^IFNγ^+^, **h**), CD8^+^GzmB^+^ T cells (gated as CD45^+^CD3^+^CD8^+^GzmB^+^, **i**), CD8^+^PD-1^+^TCF-1^+^ T cells (gated as CD45^+^CD3^+^CD8^+^PD-1^+^TCF-1^+^, **j**), CD8^+^PD-1^+^TCF-1^-^ T cells (gated as CD45^+^CD3^+^CD8^+^PD-1^+^TCF-1^-^, **k**) and CD8^+^PD-1^+^TCF-1^-^GzmB^+^ T cells (gated as CD45^+^CD3^+^CD8^+^PD-1^+^TCF-1^-^GzmB^+^, **l**) in tumor tissues of orthotopic Hepa1-6 tumor-bearing mice at 16 days after treatment indicated in (**a**). Data are presented as means ± s.d. (n = 5 mice per group; one-way ANOVA followed by Tukey’s HSD post-hoc test). **m** Colocalization of M1-like TAMs (F4/80 (red) and CD86 (pink)-positive cells) and TCF-1 (green) in tumor tissues of orthotopic Hepa1-6 tumor-bearing mice at 16 days after treatment indicated in (**a**) by immunofluorescent staining. Scale bars: 10 μm. Images are representative of three independent samples. Source data are provided as a Source Data file.

To investigate whether R848@M2pep-MPs_AFP_ could trigger an efficient tumor suppression in HCC, orthotopic Hepa1-6 tumor-bearing mice were intravenously injected with PBS, MPs, M2pep-MPs, MPs_AFP_, M2pep-MPs_AFP_, R848, R848@MPs, R848@M2pep-MPs, R848@MPs_AFP_ or R848@M2pep-MPs_AFP_ every three days for six times (Fig. 5a). Compared with free R848, R848@MPs showed a significant anticancer activity, with 70.1% reduction in tumor weight (Fig. 5b, c). R848@M2pep-MPs and R848@MPs_AFP_ exhibited stronger anticancer effects than R848@MPs, with 80.4% and 81.8% tumor inhibition (Fig. 5b, c). The strongest anticancer activity was detected in R848@M2pep-MPs_AFP_-treated group, achieving 92.3% reduction in tumor weight (Fig. 5b, c) and 43.0% reduction in the ratio of liver weight to body weight (Supplementary Fig. 19). Kaplan–Meier survival analysis revealed that the median survival time was extended to 126.5 days in R848@M2pep-MPs_AFP_-treated group (Fig. 5d), much longer than any other groups. Notably, R848@M2pep-MPs_AFP_ did not exhibit obvious toxicity, as evidenced by the body weight change (Supplementary Fig. 20) and serological analysis (Supplementary Fig. 21). The excellent anticancer activity of R848@M2pep-MPs_AFP_ was further confirmed in mice bearing large subcutaneous Hepa1-6 tumors (about 200 mm^3^) (Supplementary Fig. 22a–l). Consistently, R848@M2pep-MPs_AFP_ exhibited the strongest ability to decrease the tumor growth and prolong the survival time of Hepa1-6 tumor-bearing mice. However, no significant difference in anticancer activity was detected in R848@M2pep-MPs- and R848@M2pep-MPs_AFP_-treated mice bearing H22 liver tumors which lacked AFP expression (Supplementary Fig. 23a–h), further confirming that R848@M2pep-MPs_AFP_-reprogrammed M2-like TAMs might present AFP antigen to activate CD8^+^ T cells and exert antigen-specific killing of tumor cells.

The tumor immune microenvironment was then investigated in orthotopic Hepa1-6 tumor-bearing mice after intravenous injection of PBS, MPs, M2pep-MPs, MPs_AFP_, M2pep-MPs_AFP_, R848, R848@MPs, R848@M2pep-MPs, R848@MPs_AFP_ or R848@M2pep-MPs_AFP_ every three days for six times by flow cytometry (Fig. 5a). R848@M2pep-MPs_AFP_ significantly increased the numbers of CD4^+^ T cells (Supplementary Fig. 24a), activated CD4^+^CD69^+^ T cells (Supplementary Fig. 24b), CD8^+^ T cells (Fig. 5e), proliferated CD8^+^ T cells (Fig. 5f) and activated CD8^+^CD69^+^ T cells (Fig. 5g), CD8^+^IFNγ^+^ T cells (Fig. 5h) and CD8^+^GzmB^+^ T cells (Fig. 5i) in tumor tissues compared with free R848, R848@MPs, R848@M2pep-MPs and R848@MPs_AFP_. Meanwhile, the numbers of stem-like CD8^+^PD-1^+^TCF-1^+^ T cells (Fig. 5j), terminally exhausted CD8^+^PD-1^+^TCF-1^-^ T cells (Fig. 5k) and terminally exhausted CD8^+^ T cells secreting GzmB (CD8^+^PD-1^+^TCF-1^-^GzmB^+^ T cells, Fig. 5l) in tumor tissues were also remarkably enhanced in R848@M2pep-MPs_AFP_-treated group. These results suggested that R848@M2pep-MPs_AFP_ efficiently elicited the antitumor immunity. Meanwhile, immunofluorescence analysis showed that more stem-like CD8^+^ T cells were colocalized with M1-like TAMs in R848@M2pep-MPs_AFP_-treated group (Fig. 5m), revealing that R848@M2pep-MPs_AFP_-reprogrammed M2-like TAMs might provide a suitable APC niche for stem-like CD8^+^ T cell proliferation and differentiation. In addition, R848@M2pep-MPs_AFP_ treatment significantly improved tumor immunosuppressive microenvironment, as indicated by the decreased numbers in MDSCs and Tregs (Supplementary Fig. 24c, d). R848@M2pep-MPs_AFP_ treatment also significantly increased the numbers of the activated CD8^+^CD69^+^ T cells (Supplementary Fig. 25a), CD8^+^IFNγ^+^ T cells (Supplementary Fig. 25b) and CD8^+^GzmB^+^ T cells (Supplementary Fig. 25c) in spleens of orthotopic Hepa1-6 tumor-bearing mice, suggesting that R848@M2pep-MPs_AFP_ efficiently activated the systemic antitumor immunity.

To determine whether CD8^+^ T cell-mediated antitumor immunity was involved in the R848@M2pep-MPs_AFP_-induced anticancer activity, anti-CD8 antibody was used to deplete CD8^+^ T cells in the orthotopic Hepa1-6 tumor-bearing mice (Supplementary Fig. 26a). As expected, anti-CD8 antibody remarkably abrogated the antitumor effects of R848@M2pep-MPs_AFP_ (Supplementary Fig. 26b–d), suggesting that CD8^+^ T cells were involved in the antitumor activity of R848@M2pep-MPs_AFP_. Meanwhile, CD4^+^ T cells, but not natural killer (NK) cells were also responsible for the R848@M2pep-MPs_AFP_-induced anticancer effects (Supplementary Fig. 27a–c). In view that R848@M2pep-MPs_AFP_-reprogrammed M2-like macrophages efficiently activated CD8^+^ T cells, to investigate that the enhanced anticancer activity and antitumor immune response were mediated by R848@M2pep-MPs_AFP_-reprogrammed macrophages, clodronate liposomes were applied to deplete macrophages in the orthotopic Hepa1-6 tumor-bearing mice^50^. As expected, macrophage depletion by clodronate liposomes (Supplementary Fig. 28d) dramatically abolished the anticancer activity of R848@M2pep-MPs_AFP_ (Supplementary Fig. 28a–c) and decreased R848@M2pep-MPs_AFP_-induced increase in the numbers of CD8^+^ T cells (Supplementary Fig. 28e), proliferative CD8^+^Ki67^+^ T cells (Supplementary Fig. 28f), and activated CD8^+^CD69^+^ T cells (Supplementary Fig. 28g), CD8^+^IFNγ^+^ T cells (Supplementary Fig. 28h) and CD8^+^GzmB^+^ T cells (Supplementary Fig. 28i), confirming that R848@M2pep-MPs_AFP_-reprogrammed TAMs efficiently activated CD8^+^ T cells to exert antitumor efficacy.

M1-like macrophages can secrete chemokines and cytokines, such as CXCL9, CXCL10 and TNF-α to induce the recruitment of CD8^+^ T cells^19,21,51^. RT-qPCR results showed that R848@M2pep-MPs_AFP_-reprogrammed M2-like macrophages highly expressed *TNF-α* (Supplementary Fig. 13c) but not *CXCL9* (Supplementary Fig. 29a) or *CXCL10* (Supplementary Fig. 29b). Transwell analysis showed that R848@M2pep-MPs_AFP_-reprogrammed M2-like macrophages could efficiently recruit CD8^+^ T cells (Supplementary Fig. 30a). However, Etan significantly decreased the recruitment of CD8^+^ T cells by R848@M2pep-MPs_AFP_-educated M2-like macrophages (Supplementary Fig. 30b), suggesting that TNF-α was involved in the recruitment of CD8^+^ T cells by R848@M2pep-MPs_AFP_-reprogrammed M2-like macrophages. To further confirm this, orthotopic Hepa1-6 tumor-bearing mice were intravenously injected with R848@M2pep-MPs_AFP_ and/or intraperitoneal injection of fingolimod (FTY720) which was used to inhibit lymphocyte migration out of secondary lymphoid organs (Supplementary Fig. 31b, c), and/or intraperitoneal injection of Etan (Supplementary Fig. 31a). Treatment with FTY720 or Etan significantly abrogated R848@M2pep-MPs_AFP_-induced anticancer activity (Supplementary Fig. 31d, e) and the enhanced CD8^+^ T cell numbers in tumor tissues (Supplementary Fig. 31f), further confirming that the R848@M2pep-MPs_AFP_-induced increase of CD8^+^ T cells in tumor tissues was at least partly dependent on the recruitment of CD8^+^ T cells, and TNF-α was involved in the R848@M2pep-MPs_AFP_-induced CD8^+^ T cell recruitment into tumor tissues.

### R848@M2pep-MPs_AFP_ efficiently improve the anticancer activity against HCC and antitumor immune response of anti-PD-1 antibody

**Fig. 6:**
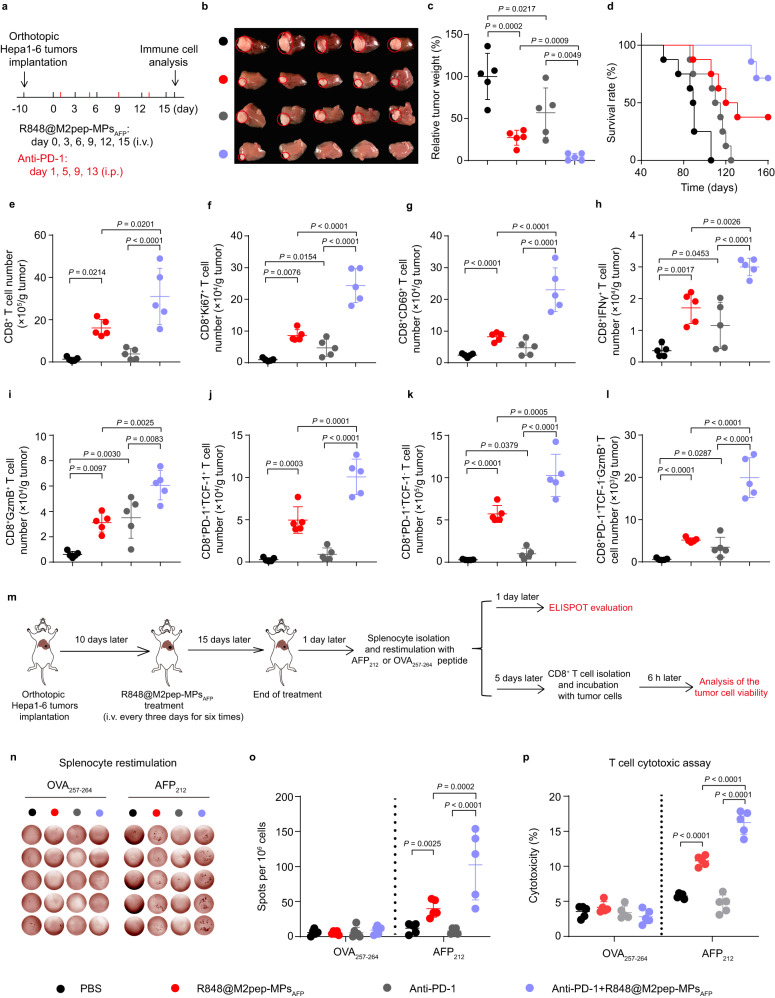
Improved antitumor effects and antitumor immunity of anti-PD-1 antibody by R848@M2pep-MPs_AFP_ in orthotopic Hepa1-6 tumor-bearing mice. **a** Schematic schedule for the antitumor experiment of combination of R848@M2pep-MPs_AFP_ and anti-PD-1 antibody in orthotopic Hepa1-6 tumor-bearing mice. **b**, **c** Tumor images (**b**) and tumor weight (**c**) of orthotopic Hepa1-6 tumor-bearing mice after treatment with R848@M2pep-MPs_AFP_ in the presence or absence of anti-PD-1 antibody at the anti-PD-1 antibody dosage of 5 mg kg^−1^ and R848 dosage of 0.5 mg kg^−1^ indicated in (**a**). Data are presented as means ± s.d. for (**c**). (n = 5 mice per group; one-way ANOVA followed by Tukey’s HSD post-hoc test). **d** Kaplan–Meier survival plot of orthotopic Hepa1-6 tumor-bearing mice after treatment indicated in (**a**). (n = 8 mice per group). **e**–**l** The numbers of CD8^+^ T cells (**e**), CD8^+^Ki67^+^ T cells (**f**), CD8^+^CD69^+^ T cells (**g**), CD8^+^IFNγ^+^ T cells (**h**), CD8^+^GzmB^+^ T cells (**i**), CD8^+^PD-1^+^TCF-1^+^ T cells (**j**), CD8^+^PD-1^+^TCF-1^-^ T cells (**k**) and CD8^+^PD-1^+^TCF-1^-^GzmB^+^ T cells (**l**) in tumor tissues of orthotopic Hepa1-6 tumor-bearing mice after treatment indicated in (**a**). Data are presented as means ± s.d. (n = 5 mice per group; one-way ANOVA followed by Tukey’s HSD post-hoc test). **m** Schematic schedule for ELISPOT and T cell cytotoxic assay. **n**, **o** Images (**n**) and numbers (**o**) of IFNγ immune spots from OVA_257-264_- or AFP_212_-restimulated splenocytes isolated from orthotopic Hepa1-6 tumor bearing mice after treatment indicated in (**m**) by the ELISPOT assay. Data are presented as means ± s.d. (n = 5 biological independent samples; two-way ANOVA followed by Bonferroni’s multiple comparisons post-test). **p** Cytotoxicity of T cells against Hepa1-6 cells when T cells isolated from OVA_257-264_- or AFP_212_-restimulated splenocytes were incubated with Hepa1-6 cells at the effector/target ratio of 10:1 for 6 h as indicated in (**m**) by LDH assay. Data are presented as means ± s.d. (n = 5 biological independent samples; two-way ANOVA followed by Bonferroni’s multiple comparisons post-test). Source data are provided as a Source Data file.

Considering that R848@M2pep-MPs_AFP_ efficiently enhanced CD8^+^ T cells and stem-like CD8^+^ T cells, remodeled tumor immunosuppressive microenvironment, and increased CD8^+^PD-1^+^ T cells (Supplementary Fig. 32a) and PD-L1^+^ tumor cells (Supplementary Fig. 32b) in tumor tissues, the effects of R848@M2pep-MPs_AFP_ on the anticancer efficacy of anti-PD-1 antibody were first evaluated in orthotopic Hepa1-6 tumor-bearing mice (Fig. 6a). As expected, R848@M2pep-MPs_AFP_ or anti-PD-1 antibody treatment significantly inhibited tumor growth, with 72.9% and 43.1% inhibition in tumor weight (Fig. 6b, c), respectively. Combination of R848@M2pep-MPs_AFP_ and anti-PD-1 antibody exhibited the strongest anticancer activity, achieving 95.9% inhibition in tumor weight (Fig. 6b, c). Kaplan–Meier survival analysis showed that 75% of mice were still alive in the R848@M2pep-MPs_AFP_ and anti-PD-1 antibody-treated group at 160 days after tumor inoculation (Fig. 6d), significantly better than other groups. R848@M2pep-MPs_AFP_ and anti-PD-1 antibody combination-elicited potent anticancer activity was further verified in mice bearing large subcutaneous Hepa1-6 tumors (Supplementary Fig. 33a–f). R848@M2pep-MPs_AFP_ and anti-PD-1 antibody combination treatment exhibited the strongest anticancer activity, resulting in 50% of mice being almost cured and 83.3% of mice being still alive at 80 days after tumor inoculation. Although combination of R848@M2pep-MPs_AFP_ and anti-PD-1 antibody increased the contents of inflammatory cytokines TNF-α (Supplementary Fig. 34a) and IFNγ (Supplementary Fig. 34b) in serum which might be due to the enhanced antitumor immunity, it did not induce significant change in serological indicators (Supplementary Fig. 35a–f), histopathological changes of major organs by Hematoxylin-eosin (H&E) staining (Supplementary Fig. 35g) and body weight (Supplementary Fig. 35h) in orthotopic Hepa1-6 tumor-bearing mice, suggesting that R848@M2pep-MPs_AFP_ and anti-PD-1 antibody treatment did not induce cytokine release syndrome to generate remarkable toxicity.

Tumor immune microenvironment analysis showed that combined treatment of R848@M2pep-MPs_AFP_ and anti-PD-1 antibody significantly increased the numbers of CD80^+^ TAMs (Supplementary Fig. 36a), CD86^+^ TAMs (Supplementary Fig. 36b) and MHC II^+^ TAMs (Supplementary Fig. 36c), while decreasing the numbers of CD206^+^ TAMs (Supplementary Fig. 36d) in tumor tissues of orthotopic HCC mice compared with R848@M2pep-MPs_AFP_ or anti-PD-1 antibody alone treatment. Meanwhile, the numbers of CD8^+^ T cells (Fig. 6e), proliferated CD8^+^ T cells (Fig. 6f), and activated CD8^+^CD69^+^ T cells (Fig. 6g), CD8^+^IFNγ^+^ T cells (Fig. 6h) and CD8^+^GzmB^+^ T cells (Fig. 6i) were the highest in tumor tissues of R848@M2pep-MPs_AFP_ and anti-PD-1 antibody-treated group. In addition, R848@M2pep-MPs_AFP_ significantly promoted the anti-PD-1 antibody-induced increase in the numbers of stem-like CD8^+^PD-1^+^TCF-1^+^ T cells (Fig. 6j), terminally exhausted CD8^+^PD-1^+^TCF-1^-^ T cells (Fig. 6k) and terminally exhausted CD8^+^ T cells secreting GzmB (Fig. 6l). The improved systemic antitumor immunity was also confirmed in the spleens of R848@M2pep-MPs_AFP_ and anti-PD-1 antibody co-treated mice (Supplementary Fig. 37a–c). Importantly, a significant increase in the numbers of effector memory T (Tem) cells (CD3^+^CD8^+^CD44^+^CD62L^−^ cells) was detected in the spleens of R848@M2pep-MPs_AFP_ and anti-PD-1 antibody co-treated mice (Supplementary Fig. 37d), suggesting that the combined treatment of R848@M2pep-MPs_AFP_ and anti-PD-1 antibody might generate immunological memory.

To further confirm the R848@M2pep-MPs_AFP_ and anti-PD-1 antibody-induced immunological memory, the splenocytes isolated from the above treated mice were exposed to the AFP_212_ or OVA_257-264_ peptide and IFNγ enzyme-linked immune absorbent spot (ELISPOT) assay was performed (Fig. 6m). Although no remarkable differences in the IFNγ spot-formation were detected in PBS-, anti-PD-1 antibody-, R848@M2pep-MPs_AFP_- and combination of R848@M2pep-MPs_AFP_ and anti-PD-1 antibody-treated group after OVA_257-264_ peptide re-stimulation, the highest frequency of IFNγ spot-forming splenocytes was detected in R848@M2pep-MPs_AFP_ and anti-PD-1 antibody-treated group after AFP_212_ peptide re-stimulation (Fig. 6n, o). Moreover, the CD8^+^ T cells isolated from splenocytes of R848@M2pep-MPs_AFP_ and anti-PD-1 antibody-treated group after AFP_212_ peptide stimulation exhibited the strongest cytotoxicity against Hepa1-6 cells (Fig. 6p). These results suggested that R848@M2pep-MPs_AFP_ and anti-PD-1 antibody-treated group produced strong antigen-specific immunological memory. The combination of R848@M2pep-MPs_AFP_ and anti-PD-1 antibody-generated immunological memory was further verified in the subcutaneous Hepa1-6 tumor-bearing mice. When these three almost cured mice in the R848@M2pep-MPs_AFP_ and anti-PD-1 antibody-treated group were re-challenged with Hepa1-6 cells in left flank and B16-OVA cells in right flank, lower tumor volume (Supplementary Fig. 33g) of Hepa1-6 tumors, but not B16-OVA tumors (Supplementary Fig. 33h) were detected compared with naïve mice, confirming that combined treatment of R848@M2pep-MPs_AFP_ and anti-PD-1 antibody generated strong antigen-specific immunological memory.

**Fig. 7:**
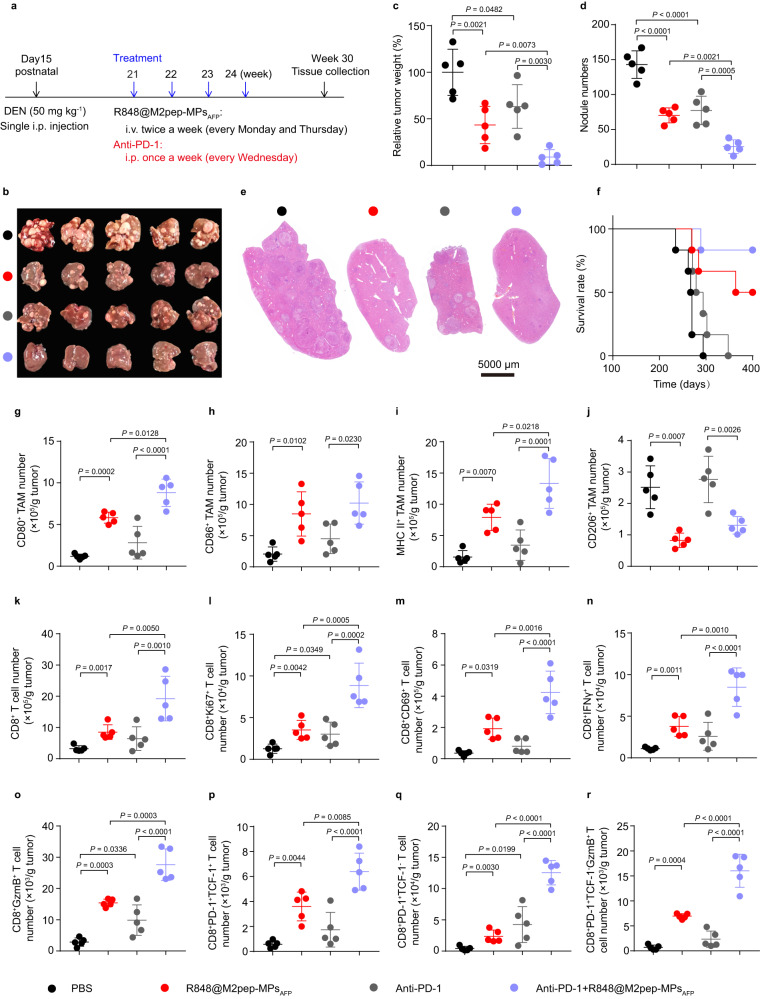
Improved antitumor activity and antitumor immunity of anti-PD-1 antibody by R848@M2pep-MPs_AFP_ in DEN-induced autochthonous HCC mice. **a** Schematic schedule for the anticancer experiment of combination of R848@M2pep-MPs_AFP_ and anti-PD-1 antibody in DEN-induced autochthonous HCC mice. **b**–**e** Tumor images (**b**), tumor weight (**c**), nodule numbers (**d**) and H&E staining of liver tissues (**e**) in DEN-induced autochthonous HCC mice at 30 weeks after treatment with R848@M2pep-MPs_AFP_ in the presence or absence of anti-PD-1 antibody at the anti-PD-1 antibody dosage of 5 mg kg^−1^ and R848 dosage of 0.5 mg kg^−1^ indicated in (**a**). Data are presented as means ± s.d. for (**c**, **d**). (n = 5 mice per group; one-way ANOVA followed by Tukey’s HSD post-hoc test). Scale bar: 5000 μm for (**e**). **f** Kaplan–Meier survival plot of DEN-induced autochthonous HCC mice after treatment indicated in (**a**). (n = 6 mice per group). **g**–**r** The numbers of CD80^+^ TAMs (**g**), CD86^+^ TAMs (**h**), MHC II^+^ TAMs (**i**), CD206^+^ TAMs (**j**), CD8^+^ T cells (**k**), CD8^+^Ki67^+^ T cells (**l**), CD8^+^CD69^+^ T cells (**m**), CD8^+^IFNγ^+^ T cells (**n**), CD8^+^GzmB^+^ T cells (**o**), CD8^+^PD-1^+^TCF-1^+^ T cells (**p**), CD8^+^PD-1^+^TCF-1^-^ T cells (**q**) and CD8^+^PD-1^+^TCF-1^-^GzmB^+^ T cells (**r**) in tumor tissues of DEN-induced autochthonous HCC mice at 30 weeks after treatment indicated in (**a**). Data are presented as means ± s.d. (n = 5 mice per group; one-way ANOVA followed by Tukey’s HSD post-hoc test). Source data are provided as a Source Data file.

The ameliorated anticancer activity and tumor immune microenvironment induced by the combination of R848@M2pep-MPs_AFP_ and anti-PD-1 antibody were further confirmed in diethylnitrosamine (DEN)-induced autochthonous HCC models (Fig. 7a). As expected, R848@M2pep-MPs_AFP_ or anti-PD-1 antibody alone significantly inhibited tumor growth, with 56.6% or 36.7% reduction in tumor weight (Fig. 7b, c), and 50.9% or 46.0% reduction in tumor nodule numbers (Fig. 7b, d), respectively. However, the combination of R848@M2pep-MPs_AFP_ and anti-PD-1 antibody generated the strongest tumor suppression, with 91.0% and 82.2% inhibition in tumor weight and tumor nodules. H&E staining of liver tissues (Fig. 7e) and longer survival time of HCC mice (Fig. 7f) further confirmed the excellent synergistic antitumor activity of the combination of R848@M2pep-MPs_AFP_ and anti-PD-1 antibody. Consistently, the tumor immune microenvironment analysis showed that the combination of R848@M2pep-MPs_AFP_ and anti-PD-1 antibody exhibited the highest numbers of M1-like TAMs (Fig. 7g–i), CD8^+^ T cells (Fig. 7k), proliferated CD8^+^ T cells (Fig. 7l), activated CD8^+^CD69^+^ T cells (Fig. 7m), CD8^+^IFNγ^+^ T cells (Fig. 7n) and CD8^+^GzmB^+^ T cells (Fig. 7o), stem-like CD8^+^PD-1^+^TCF-1^+^ T cells (Fig. 7p), terminally exhausted CD8^+^PD-1^+^TCF-1^-^ T cells (Fig. 7q) and CD8^+^PD-1^+^TCF-1^-^GzmB^+^ T cells (Fig. 7r) compared with R848@M2pep-MPs_AFP_- or anti-PD-1 antibody-treated group, while decreased the M2-like TAMs (Fig. 7j). The R848@M2pep-MPs_AFP_ and anti-PD-1 antibody-ameliorated immune microenvironment was further verified in spleens of autochthonous HCC mice (Supplementary Fig. 38a–d). These results strongly supported the notion that R848@M2pep-MPs_AFP_ efficiently improved the anti-PD-1 antibody-triggered anticancer activity and antitumor immunity in HCC.

### R848@M2pep-MPs_OVA_ efficiently boost anti-PD-1 therapy against B16-OVA tumors

**Fig. 8:**
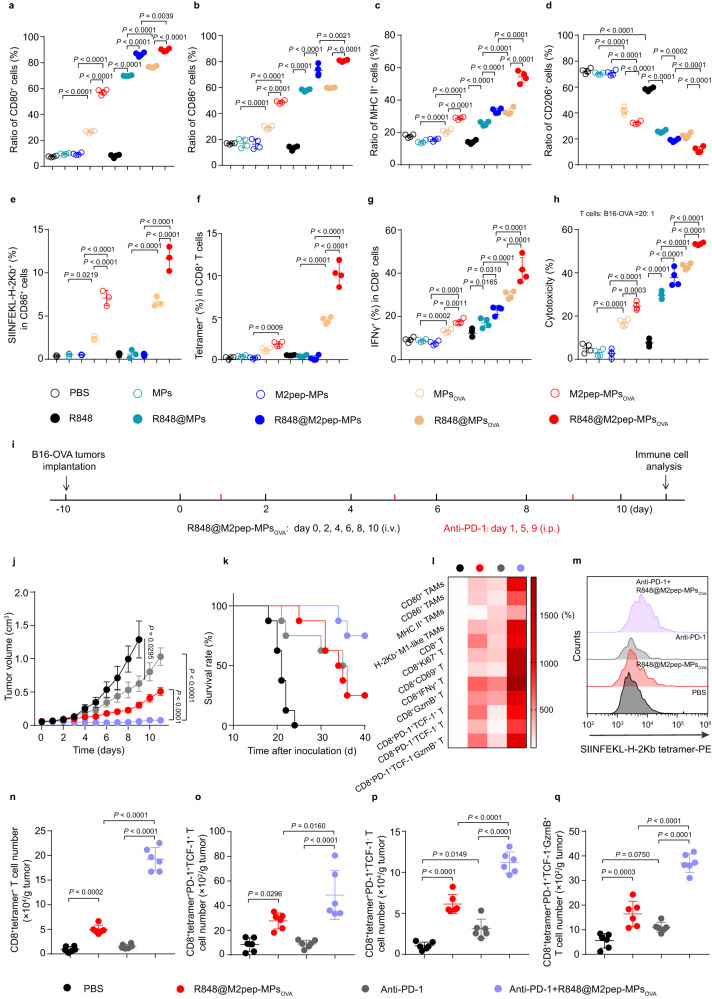
Improved anticancer activity and antitumor immunity of anti-PD-1 antibody by 848@M2pep-MPs_OVA_ in B16-OVA tumor-bearing mice. **a**–**e** Percentages of CD80^+^ (**a**), CD86^+^ (**b**), MHC II^+^ (**c**), and CD206^+^ cells (**d**) and SIINFEKL-H-2Kb^+^ M1-like macrophages (**e**) in IL-4-stimulated RAW264.7 cells after treatment with the indicated formulations at R848 concentration of 2 nM for 24 h. Data are presented as means ± sd (*n* = 4 and 3 biological independent samples for (**a**–**d**) and (**e**), respectively; one-way ANOVA followed by Tukey’s HSD post-hoc test). **f**, **g** Percentages of SIINFEKL-H-2Kb tetramer^+^ (**f**) and IFNγ^+^ cells (**g**) in CD8^+^ T cells at 5 days after co-culture with IL-4-stimulated RAW264.7 cells pretreated indicated in (**a**–**e**). Data are presented as means ± sd (*n* = 4 biological independent samples; one-way ANOVA followed by Tukey’s HSD post-hoc test). **h** Cytotoxicity of CD8^+^ T cells treated indicated in (**f**, **g**) against B16-OVA cells after co-incubation at the effector/target ratio of 20:1 for 6 h. Data are presented as means ± s.d. (*n* = 4 biological independent samples; one-way ANOVA followed by Tukey’s HSD post-hoc test). **i** Schematic schedule for anticancer experiment of combination of R848@M2pep-MPs_OVA_ and anti-PD-1 antibody. **j**, **k** Tumor growth curves (**j**) and Kaplan–Meier survival plots (**k**) of B16-OVA tumor-bearing mice after treatment with R848@M2pep-MPs_OVA_ in the presence or absence of anti-PD-1 antibody at anti-PD-1 antibody and R848 dosage of 5 and 0.5 mg kg^−1^ indicated in (**i**), respectively. Data are presented as means ± sem for (**j**). (*n* = 6 and 8 mice per group for (**j**) and (**k**), respectively; one-way ANOVA followed by Tukey’s HSD post-hoc test). **l** Relative immune cell numbers in tumors of B16-OVA tumor-bearing mice after treatment indicated in (**i**). (*n* = 6 mice per group). **m**–**q** Representative flow plots of SIINFEKL-H-2Kb tetramer^+^ in CD3^+^CD8^+^T cells (**m**) and numbers of CD8^+^tetramer^+^ (**n**), CD8^+^tetramer^+^PD-1^+^TCF-1^+^ (**o)**, CD8^+^tetramer^+^PD-1^+^TCF-1^-^ (**p**) and CD8^+^tetramer^+^PD-1^+^TCF-1^-^GzmB^+^ T (**q**) cells in tumors of B16-OVA tumor-bearing mice after treatment indicated in (**i**). Data are presented as means ± s.d. (*n* = 6 mice per group; one-way ANOVA followed by Tukey’s HSD post-hoc test). Source data are provided as a Source Data file.

Since antitumor immunity depends on tumor antigen-specific T cell responses, tumor antigens are critical for the initiation of antitumor immunity^52,53^. In order to explore the universal application of R848@M2pep-MPs_Ag_ (Ag represents specific antigen), RAW264.7 cells overexpressing the model antigen OVA were constructed (Supplementary Fig. 39a) and R848-loading M2pep-modified MPs derived from RAW264.7_OVA_ cells (Supplementary Fig. 39b) were collected. Consistently, compared with R848, R848@MPs, R848@M2pep-MPs and R848@MPs_OVA_, R848@M2pep-MPs_OVA_ exhibited the strongest capacity to reprogram M2-like macrophages into M1-like phenotype, as indicated by the enhanced protein expressions of M1-related markers including CD80 (Fig. 8a), CD86 (Fig. 8b) and MHC II (Fig. 8c), and mRNA expressions including *CD80* (Supplementary Fig. 40a), *CD86* (Supplementary Fig. 40b) and *iNOS* (Supplementary Fig. 40c), and decreased protein expressions of M2-related markers including CD206 (Fig. 8d) and mRNA expressions including *Mgl1* (Supplementary Fig. 40d) and *Mrc1* (Supplementary Fig. 40e). Besides, the supernatants of R848@M2pep-MPs_OVA_-treated M2-like macrophages exhibited the strongest cytotoxicity against B16-OVA cells (Supplementary Fig. 40f), further confirming the excellent M2-like macrophage repolarization capacity. In addition, R848@M2pep-MPs_OVA_-treated M2-like macrophages exhibited the highest percentage of CD86^+^SIINFEKL-H-2Kb^+^ cells (Fig. 8e), revealing the efficient antigen process and presentation of OVA by R848@M2pep-MPs_OVA_-treated M2-like macrophages. Consistently, R848@M2pep-MPs_OVA_-treated M2-like macrophages showed the strongest capacity to increase the percentages of OVA-specific CD8^+^ T cells (Fig. 8f) and CD8^+^IFNγ^+^ T cells (Fig. 8g) and the strongest cytotoxicity against B16-OVA cells (Fig. 8h and Supplementary Fig. 41a). However, no significant difference in the cytotoxicity against Hepa1-6 cells was observed in CD8^+^ T cells activated by R848@M2pep-MPs- and R848@M2pep-MPs_OVA_-treated M2-like macrophages (Supplementary Fig. 41b, c). These results demonstrated that R848@M2pep-MPs_OVA_, like R848@M2pep-MPs_AFP_, efficiently reprogrammed M2-like macrophages into M1-like phenotype and presented OVA to induce antigen-specific CD8^+^ T cell activation.

The antitumor activity elicited by the combination of R848@M2pep-MPs_OVA_ and anti-PD-1 antibody was further investigated in B16-OVA tumor-bearing mice (Fig. 8i). As expected, the combination of R848@M2pep-MPs_OVA_ and anti-PD-1 antibody significantly inhibited the tumor growth compared with R848@M2pep-MPs_OVA_ or anti-PD-1 antibody (Supplementary Fig. 42a–d and Fig. 8j), exhibiting synergistic anticancer effects. Kaplan–Meier survival analysis revealed the longest survival time in the R848@M2pep-MPs_OVA_ and anti-PD-1 antibody-treated group, with 75% of mice being alive at 40 days after tumor inoculation (Fig. 8k). Moreover, the immune microenvironment analysis in tumor tissues showed that the combination of R848@M2pep-MPs_OVA_ and anti-PD-1 antibody efficiently enhanced the numbers (Fig. 8l and Supplementary Fig. 43a–c) and OVA antigen presentation (Fig. 8l and Supplementary Fig. 43d) of M1-like TAMs compared with R848@M2pep-MPs_OVA_ or anti-PD-1 antibody. Meanwhile, the numbers of CD8^+^ T cells (Fig. 8l and Supplementary Fig. 43e), proliferative CD8^+^ T cells (Fig. 8l and Supplementary Fig. 43f), activated CD8^+^ T cells (Fig. 8l and Supplementary Fig. 43g–i), stem-like CD8^+^ T cells (Fig. 8l and Supplementary Fig. 43j), terminally exhausted CD8^+^ T cells (Fig. 8l and Supplementary Fig. 43k) and terminally exhausted CD8^+^ T cells secreting GzmB (Fig. 8l and Supplementary Fig. 43l) in tumor tissues were the highest in R848@M2pep-MPs_OVA_ and anti-PD-1 antibody-treated group. More importantly, a significant increase in the numbers of OVA-specific CD8^+^ T cells (Fig. 8m, n), OVA-specific stem-like CD8^+^ T cells (Fig. 8o), OVA-specific terminally exhausted CD8^+^ T cells (Fig. 8p) and OVA-specific terminally exhausted CD8^+^ T cells secreting GzmB (Fig. 8q) was detected in the tumors of R848@M2pep-MPs_OVA_ and anti-PD-1 antibody-treated group. The improved systemic antitumor immunity and immune memory was further verified in spleens (Supplementary Fig. 44a–k) and tumor-draining lymph nodes (Supplementary Fig. 45a–l) of the R848@M2pep-MPs_OVA_ and anti-PD-1 antibody-treated group. These results reveal that R848@M2pep-MPs_Ag_ might function as a platform to boost anti-PD-1 therapy against tumors expressing alloantigen.

## Discussion

M2-like TAMs, representing the major compartment of infiltrated immune cells, play an important role in promoting angiogenesis, tumor invasion and metastasis, drug resistance and immunosuppression in HCC^17,54^. M2-like TAMs were highly related to poor prognosis, short survival time, and poor responsiveness to anti-PD-1 antibody therapy^18,55^. Recent work has shown that lymphocyte-depleted subtypes of HCC displayed a more prominent macrophage signature with suppression of Th1 immune reaction and a high M2 response^16,56^, revealing that M2-like TAMs reversely regulated the antitumor immunity in HCC. Here, our work also confirmed that the responsiveness of HCC to anti-PD-1 antibody was associated with the number and activation of M1-like TAMs and CD8^+^ T cells including stem-like CD8^+^ T cells, indicating that the reprogramming of M2-like TAMs to M1-like phenotype might help to improve the therapeutic efficacy of anti-PD-1 antibody. However, only activating innate immunity by regulating TAMs was difficult to achieve ideal therapeutic efficacy. Developing effective TAM-directed adaptive immunotherapeutic strategies would further boost anti-PD-1 therapy.

It was reported that stem-like CD8^+^ T cells that express TCF1 remained responsive to checkpoint blockade therapy, suggesting that the state of T cells, in addition to the number and spatial distribution of T cells, was critical for the induction of effective tumor immunity^9,10,13,57^. In this work, the anti-PD-1 antibody-responsive tumors of orthotopic Hepa1-6 tumor-bearing mice had more stem-like CD8^+^PD-1^+^TCF-1^+^ T cells and terminally exhausted CD8^+^PD-1^+^TCF-1^-^ T cells, revealing that increasing the number of stem-like CD8^+^ T cells was vital for the enhanced anti-PD-1 therapy in HCC. Tumor therapeutic vaccination had been shown to generate stem-like CD8^+^ T cells^10,13^. For example, vaccination of tumor antigen gp33 peptide plus adjuvant (TLR3 ligand [poly(I:C)]) expanded CD8^+^PD-1^+^TCF-1^+^ T cells (176-fold) 1 week later and these cells remained present in elevated numbers at endpoint in mice bearing B16 cells expressing gp33 (B16-gp33 cells). Moreover, combination of the vaccination and wild-type CD8^+^ T cells specific for tumor antigen gp33 (P14) exhibited longer protective effects than the combination of vaccination and *Tcf7* ^−/−^ P14 cells^13^, suggesting that extended tumor control depended on TCF1. In addition, intravenous injection of a self-assembling nanoparticle vaccine platform co-delivering tumor antigen and TLR7/8 agonist (SNP-7/8a) resulted in a higher frequency of CD8^+^PD-1^+^TCF-1^+^ cells and a significant tumor growth inhibition^9^. Thus, developing tumor vaccine-like drug might provide a promising strategy to increase stem-like CD8^+^ T cells.

Stem-like T cells were reported to reside in dense APC niches within the tumors, and tumors that failed to form these structures were not extensively infiltrated by T cells^15^. Considering that TAMs are highly infiltrated in the tumor tissues and M1-like TAMs serve as APCs^23,25^, developing TAM-directed adaptive immunity by reprogramming M2-like TAMs to M1-like phenotype and then presenting tumor-associated antigens to promote and activate antigen-specific CD8^+^ T cells, including stem-like CD8^+^ T cells might achieve comprehensive cancer immunotherapy for improved anti-PD-1 therapy. In this work, we successfully developed M2pep-conjugated MPs derived from AFP-overexpressing macrophages to load R848 (R848@M2pep-MPs_AFP_) for improved anti-PD-1 therapy in HCC. M2pep-MPs_AFP_ efficiently delivered R848 to M2-like TAMs and reprogrammed them to M1-like phenotype in virtue of the nature tumor tropism of macrophage-derived MPs and further M2pep modification. The R848@M2pep-MPs_AFP_-reprogrammed M2-like TAMs not only relieved tumor immunosuppressive microenvironment, but also functioned as APCs to present AFP antigen with the help of the adjuvant R848 to drive CD8^+^ T cell-mediated antigen-specific antitumor immunity. Here, there were some evidences to prove the importance of AFP antigen presentation in this system: (1) The activated CD8^+^ T cells by R848@M2pep-MPs_AFP_-reprogrammed M2-like TAMs possessed the strongest cytotoxicity against Hepa1-6 cells which expressed AFP, but not B16-OVA cells. (2) TAMs isolated from R848@M2pep-MPs_AFP_-treated orthotopic Hepa1-6 tumor-bearing mice could directly activate CD8^+^ T cells in vitro. (3) R848@M2pep-MPs_AFP_-treated group had the strongest ability to kill AFP_212_-labeled target cells using an antigen-specific in vivo killing assay. All these data supported that R848@M2pep-MPs_AFP_-reprogrammed M2-like TAMs could efficiently present AFP to activate antigen-specific CD8^+^ T cell antitumor immunity. Furthermore, more TCF1^+^ cells were localized in M1-like TAMs in R848@M2pep-MPs_AFP_-treated group, revealing that the R848@M2pep-MPs_AFP_-reprogrammed M2-like TAMs might provide an intra-tumoral niche to maintain and differentiate stem-like CD8^+^ T cells. Thus, R848@M2pep-MPs_AFP_ remarkably boosted anti-PD-1 therapy in HCC, inducing stem-like CD8^+^ T cell proliferation and differentiation into terminally exhausted CD8^+^ T cells to exert long-term antitumor effects. In addition, combination of R848@M2pep-MPs_AFP_ and anti-PD-1 antibody generated strong antitumor immune memory to exert long-term antitumor effects, as evidenced by the facts that (1) a significant increase in the numbers of CD8^+^ Tem cells was detected in the spleens of R848@M2pep-MPs_AFP_ and anti-PD-1 antibody co-treated orthotopic Hepa1-6 tumor-bearing mice and DEN-induced autochthonous HCC models; (2) the highest frequency of IFNγ spot-forming splenocytes was detected in splenocytes isolated from the R848@M2pep-MPs_AFP_ and anti-PD-1 antibody-treated mice after stimulation with AFP_212_ peptide by ELISPOT assay, and these splenocytes exhibited the strongest cytotoxicity against Hepa1-6 cells; (3) the almost cured subcutaneous Hepa1-6 tumor-bearing mice after R848@M2pep-MPs_AFP_ and anti-PD-1 antibody combination treatment resisted the re-challenge of Hepa1-6 cells, but not B16-OVA cells. Thus, this work challenged the general routine of activating antitumor immunity using DCs as APCs^58,59^, and efficiently overcame major limitations of anti-PD-1 therapy by reprogramming TAMs highly enriched in tumor tissues.

Cancer immunotherapies, which target neoantigens, could lead to a precise treatment for cancer patients^60,61^. In addition to the fabrication of R848@M2pep-MPs_AFP_ for enhanced anti-PD-1 therapy in HCC, R848@M2pep-MPs_OVA_ derived from a model antigen OVA-overexpressed macrophages were developed to significantly improve the therapeutic efficacy of anti-PD-1 antibody in B16-OVA tumor-bearing mice by reprogramming M2-like TAMs into M1-like phenotype, followed by presenting OVA to activate antigen-specific CD8^+^ T cell response and promoting the proliferation and differentiation of stem-like CD8^+^ T cells. This work revealed that the constructed platform could achieve personalized cancer immunotherapy with anti-PD-1 antibody by integrating personalized tumor antigens to macrophage-derived MPs. For example, R848@M2pep-MPs_Ag_ derived from RAW264.7 cells stably overexpressing the lung cancer-associated antigen melanoma-associated antigen (MAGE) A3 or pancreatic cancer-associated antigen MUCIN-4 (MUC4) might be used to treat lung cancer or pancreatic tumors, respectively.

Despite the encouraging efficacy of the combination of R848@M2pep-MP_Ag_ and anti-PD-1 antibody for cancer treatment, there remain some concerns about translating this platform from the bench to human clinical trials. Firstly, the precise prediction of tumor-specific neoantigens that can induce cytotoxic T cells in individual patients is essential. With the significant advances in cancer genomics using next-generation sequencing and bioinformatic technologies, the rapid identification and screening of tumor neoantigens were expected to accelerate the development of this personalized cancer immunotherapy for a broader range of cancer patients. Secondly, the macrophage resource for R848@M2pep-MPs_Ag_ construction is another critical issue. It would be better to use human peripheral blood monocyte-derived macrophages (MDMs) as the donor cells to prepare R848@M2pep-MPs_Ag_ in order to avoid the recognition by immune cells. However, the limited expansion and low efficiency of gene transfection of MDMs, which are the primary cells, restricts the large-scale production of MPs_Ag_. Thus, macrophages derived from human monocytic leukemia THP-1 cells might be instead used to produce MPs_Ag_. Thirdly, M2pep primarily serves as a proof-of-concept for targeting M2-like macrophages rather than a strategy that can be used to target human M2-like macrophages because of the differences in gene expression between human and mouse macrophages^42^. For translating this platform to human clinical trials, targeting ligands for human M2-like macrophages need to be further identified. Finally, the scale-up process, quality control standards for MP content, and drug loading efficiency in the GMP production department are required to expedite the clinical translation.

In summary, the data in this study show that R848@M2pep-MPs_AFP_ efficiently target and reprogram M2-like TAMs into M1-like phenotype. The R848@M2pep-MPs_AFP_-reprogrammed M2-like TAMs not only present AFP to activate antigen-specific CD8^+^ T cell antitumor immunity, but also provide an intra-tumoral niche to proliferate and differentiate stem-like CD8^+^ T cells to terminally exhausted CD8^+^ T cells to exert long-term antitumor effects in combination with anti-PD-1 antibody. Our work demonstrates a strong and personalized strategy to boost anti-PD-1 therapy.

## Methods

### Materials

RPMI 1640 medium, Dulbecco’s Modified Eagles Medium (DMEM) medium, phosphate-buffered saline (PBS), and trypsin were purchased from HyClone (GE Healthcare, South Logan, UT, USA). Fetal bovine serum (FBS), collagenase I, and penicillin/streptomycin were purchased from Gibco BRL/Life Technologies (Grand Island, NY, USA). Cytokines like recombinant mouse IL-4, macrophage colony stimulating factor (M-CSF), TNF-α, and IFNγ were obtained from PeproTech (Rocky Hill, NJ, USA). PKH26, IR780, LPS, FTY720, and DEN were obtained from Sigma-Aldrich (St Louis, MO, USA). R848 was purchased from MedChemExpress (Monmouth Junction, NJ, USA). 1,2-distearoyl-sn-glycero-3-phosphoethanolamine -*N*-[maleimide (polyethyleneglycol)] (DSPE-PEG-Mal) was provided by Ponsure (Shanghai, China). The anti-CD8, anti-CD4, anti-NK1.1, and anti-PD-1 antibodies were purchased from BioXcell (West Lebanon, NH, USA). Clodronate liposome was obtained from Yeasen (Shanghai, China). The peptides M2pep (CYEQDPWGVKWWYK), AFP_212_ (GSMLNEHVM), and OVA_257-264_ (SIINFEKL) were synthesized by Bankpeptide Biological Technology Co., Ltd. (Hefei, China). Matrigel was purchased from Corning (New York, NY, USA). The SIINFEKL-H-2Kb tetramer was obtained from HelixGen (Guangzhou, China). Antibodies used for flow cytometric analysis were purchased from BioLegend (San Diego, CA, USA).

### Cells and animals

H22 and RAW264.7 cells were provided from the Type Culture Collection of the Chinese Academy of Sciences (Shanghai, China). B16-OVA cells were kindly provided by Prof. Bo Huang (Institute of Basic Medical Sciences, Chinese Academy of Medical Sciences, Beijing, China). Hepa1-6 cells were purchased from Boster Biological Technology Ltd (Wuhan, China). RAW264.7 and Hepa1–6 cells were cultured in DMEM medium, and H22 and B16-OVA cells were cultured in RPMI 1640 medium containing 10% FBS and 1% penicillin/streptomycin in a 5% CO_2_ incubator at 37 °C. Murine BMDMs were obtained as previously described^19^. Briefly, the C57BL/6 male mice (6–8 weeks old) were anesthetized and sacrificed, and bone marrow cells were isolated from femurs and tibia and then cultured in RPMI 1640 complete growth medium containing 20 ng mL^−1^ recombinant mouse M-CSF for 5 days. RAW264.7 cells or BMDMs were treated with 20 ng mL^−1^ IFNγ and 100 ng mL^−1^ LPS for 24 h to acquire M1-like macrophages, and M2-like macrophages were acquired by treating RAW264.7 cells or BMDMs with 20 ng mL^−1^ IL-4 for 24 h.

RAW264.7_AFP_ cells were constructed by infecting RAW264.7 cells with lentivirus expressing murine AFP gene. Briefly, 293 T cells were transfected with the plasmids including psPAX2, pM2.GVSVG and pCDH-CMV-MCS-EF1-Puro-AFP at the ratio of 3:1:4 (total 16 μg) using 20 μL polyethyleneimine (PEI). The viruses were harvested through 0.45 μm filters and titred 48-72 h later. RAW264.7 cells were seeded on 12-well plates and infected with 5 × 10^6^ plaque formation unit (PFU) of AFP-expressing lentivirus for 24 h, followed by selection with 2 µg mL^−1^ puromycin to obtain RAW264.7_AFP_ cells. RAW264.7_OVA_ cells were constructed in a similar way using the plasmids psPAX2, pM2.GVSVG and pCDH-OVA-Zeocin.

Male or female C57BL/6 mice, male BALB/c mice and male C3H/HeN mice (6–8 weeks old) were purchased from Beijing Vital River Laboratory Animal Technology Co., Ltd. (Beijing, China). Mice were housed in groups of 6 mice per individually ventilated cage in a 12 h light-dark cycle, with constant room temperature 21  ±  1 °C and relative humidity 40–70%. All mice had free access to food and water. Subcutaneous H22 or B16-OVA tumor-bearing mice were constructed by subcutaneously injecting 2 × 10^6^ H22 cells or 5 × 10^5^ B16-OVA cells per mouse into the right flanks of male BALB/c mice or male C57BL/6 mice. Subcutaneous Hepa1-6 tumor-bearing mice were constructed by subcutaneously injecting 3 × 10^6^ Hepa1-6 cells in 100 μL PBS/Matrigel (1:1) per mouse into the right flanks of male C57BL/6 mice. To establish orthotopic HCC mice, subcutaneous Heap1-6 tumor tissues reaching 500 mm^3^ were peeled and cut into about 2 mm × 2 mm × 2 mm pieces. Male C57BL/6 mice were anesthetized, and the tumor pieces were implanted into the left lobe of liver. To establish DEN-induced autochthonous HCC mice^39,41^, female C57BL/6 mice and male C3H/HeN mice were cross-bred to obtain B6C3 F_1_ mice. Then fifteen-day-old neonatal B6C3 F_1_ male mice were intraperitoneally injected with 50 mg kg^−1^ DEN. All animal experiments were carried out under the guidance of the Institutional Animal Care and Use Committee at Tongji Medical College, Huazhong University of Science and Technology (Wuhan, China). Maximum tumor volume is 1500 mm^3^ according to the Institutional Animal Care and Use Committee approved protocol, and mice were euthanized when tumor reached this volume.

### RNA-seq analysis

Mice bearing orthotopic Hepa1-6 tumors were intraperitoneally injected with PBS or anti-PD-1 antibody (clone RMP1-14, BioXcell) on day 10, 14, 18, 22, and 26. On day 27, the whole tumors were collected and the surrounding tissue was removed. The tumors that had an intermediate response were excluded, and three largest tumors (the nonresponsive tumors) and three smallest tumors (the responsive tumors) compared with PBS-treated group were frozen in liquid nitrogen immediately. The RNA extraction, library preparation and sequencing were performed by Novogene (Beijing, China).

### Preparation and characterization of R848@M2pep-MPs_AFP_

DSPE-PEG-M2pep was first synthesized by conjugating M2pep with DSPE-PEG-Mal via Michael addition reaction at room temperature for 4 h. RAW264.7 or RAW264.7_AFP_ cells were irradiated with ultraviolet (300 J m^−2^) for 1 h, followed by treatment with 0.2 mg mL^−1^ R848. After 12 h treatment, the cell supernatants were collected and centrifuged at 600 *g* for 10 min to remove the cells and cell debris, then centrifuged at 18,000 *g* for 1 h to collect R848@MPs or R848@MPs_AFP_. R848@MPs or R848@MPs_AFP_ were further incubated with DSPE-PEG-M2pep at a mass ratio of 50:1 at 4 °C for 24 h to prepare R848@M2pep-MPs or R848@M2pep-MPs_AFP_. The blank MPs, M2pep-MPs, MPs_AFP_ and M2pep-MPs_AFP_ were constructed in the same way without adding R848. The concentration of R848 in R848@M2pep-MPs_AFP_ was determined by HPLC system (Agilent 1100, USA). The chromatography was performed as follows: column, C18 column (5 × 250 mm, particle size 5 μm); mobile phase, acetonitrile-water containing 10% glacial acetic acid (2: 98, v/v, 0–2 min; 100: 0, v/v, 2–12 min; 2: 98, v/v, 12–25 min); flow rate, 0.5 mL min^−1^; detection wavelength, 320 nm. The hydrodynamic diameter and zeta potential of R848@M2pep-MPs_AFP_ were determined by Zetasizer Nano ZS 90 (Malvern Instruments Ltd., Worcestershire, UK). Their morphology was observed by AFM (Multi-Mode 8, Bruker, Santa Barbara, USA). In vitro R848 release from R848@M2pep-MPs and R848@M2pep-MPs_AFP_ was determined by dialysis method. Briefly, R848@M2pep-MPs or R848@M2pep-MPs_AFP_ (10 μg R848 content) were put into the dialysis bags with the molecular weight cut-off of 2000 Da, submerged into 10 mL of PBS at different pH values, and then stirred with 300 rpm at 37 °C. 0.1 mL of sample solution was collected and replaced with equal volume of fresh PBS at the indicated time intervals. R848 content in sample solution was detected by HPLC.

### Cellular uptake and intracellular trafficking

For the cellular uptake analysis, M0, M1, M2-like macrophages and Hepa1-6 cells were incubated with PKH26-labeled MPs, M2pep-MPs, MPs_AFP_ or M2pep-MPs_AFP_ at the concentration of 10 μg protein mL^−1^ in the presence or absence of 5 μg mL^−1^ M2pep for 4 h. The cells were washed with PBS for three times and intracellular PKH26 fluorescence was detected using flow cytometry (CytoFlex S, Beckman Coulter, Fullerton, CA, USA). For the intracellular trafficking analysis, M2-like macrophages were treated with 10 μg protein mL^−1^ of RhB-loaded DiO-labeled MPs_AFP_ or M2pep-MPs_AFP_. At the designated time intervals, the cells were labeled with 75 nM LysoTracker® Deep Red (ThermoFisher Scientific, USA) for lysosomes, and 1 µg mL^−1^ DAPI (Yeason, Shanghai, China) for nuclei after fixing with 4% paraformaldehyde. The fluorescence images of cells were observed by confocal microscopy (FV3000, Olympus, Tokyo, Japan).

### Bio-distribution in vivo

Orthotopic Hepa1-6 tumor-bearing mice at 15 days after tumor inoculation were intravenously administrated with IR-780-labeled MPs, M2pep-MPs, MPs_AFP_ or M2pep-MPs_AFP_ at the dosage of 15 mg protein kg^−1^. At 24 h after injection, the mice, and the stripped major tissues (heart, normal liver, spleen, lung and kidney) and tumors were imaged by a Caliper IVIS Lumina II in vivo imaging system (PerkinElmer, Waltham, MA, USA).

### Cellular uptake in vivo

Orthotopic Hepa1-6 tumor-bearing mice at 15 days after tumor inoculation were intravenously administrated with PKH26-labeled MPs, M2pep-MPs, MPs_AFP_ or M2pep-MPs_AFP_ at the dosage of 15 mg protein kg^−1^. At 24 h post-injection, the mice were sacrificed, and the livers, spleens, lungs, kidneys and tumors were harvested. The tissues were minced with scissors and then incubated with RPMI 1640 medium containing 0.8 mg mL^−1^ collagenase I and 5 μg mL^−1^ DNase I at 37 °C for 30 min. The homogenates were passed through a 200-mesh cell strainer, treated with red blood cells lysis buffer and then washed with PBS to acquire single cell suspensions. For PKH26 fluorescence analysis in macrophages (TAMs, Kupffer cells, splenic macrophages, pulmonary macrophages and renal macrophages), the cells were stained with PerCP/Cyanine5.5 anti-CD11b (Biolegend, cat. No 101228, clone M1/70, 1/80 dilution) and Brilliant Violet 421^TM^ anti-F4/80 (Biolegend, cat. No 123137, clone BM8, 1/100 dilution). For PKH26 fluorescence analysis in M1/M2-like TAMs, the cells were stained with PerCP/Cyanine5.5 anti-CD11b, Brilliant Violet 421^TM^ anti-F4/80, PE/Cyanine7 anti-CD80 (Biolegend, cat. No 104734, clone 16-10A1, 1/50 dilution) or APC anti-CD206 (Biolegend, cat. No 141708, clone C068C2, 1/50 dilution). For PKH26 fluorescence analysis in T cells, the cells were stained with Brilliant Violet 510^TM^ anti-CD45 (Biolegend, cat. No 103137, clone 30-F11, 1/20 dilution) and PE/Cyanine7 anti-CD3 (Biolegend, cat. No 100220, clone 17A2, 1/100 dilution). For PKH26 fluorescence analysis in DCs, the cells were stained with APC anti-CD45 (Biolegend, cat. No 103112, clone 30-F11, 1/100 dilution), Brilliant Violet 421^TM^ anti-F4/80 and PE/Cyanine7 anti-CD11c (Biolegend, cat. No 117317, clone N418, 1/80 dilution). For PKH26 fluorescence analysis in MDSCs, the cells were stained with APC anti-CD45, PerCP/Cyanine5.5 anti-CD11b and Brilliant Violet 421 anti-Ly-6G/Ly-6C (Gr-1) (Biolegend, cat. No 108433, clone RB6-8C5, 1/20 dilution). For PKH26 fluorescence analysis in Tregs, cells were firstly stained with Brilliant Violet 510^TM^ anti-CD45, PE/Cyanine7 anti-CD3, PerCP/Cyanine5.5 anti-CD4 (Biolegend, cat. No 100539, clone RM4-5, 1/80 dilution) and APC anti-CD25 (Biolegend, cat. No 102011, clone PC61, 1/100 dilution). After surface staining, the cells were further treated with transcription factor buffer set (BD pharmingen, cat. No 562574) and re-stained with Brilliant Violet 421^TM^ anti-FoxP3 (Biolegend, cat. No 126419, clone MF-14, 1/50 dilution). All antibodies were used in accordance with the manufacturer’s instructions and incubated with cells for 30 min in dark at room temperature. The samples were analyzed by flow cytometry.

### Reprogramming of M2-like macrophages

IL-4-stimulated RAW264.7 cells or BMDMs were treated with PBS, MPs, M2pep-MPs, MPs_AFP_, M2pep-MPs_AFP_, R848, R848@MPs, R848@M2pep-MPs, R848@MPs_AFP_ or R848@M2pep-MPs_AFP_ at the R848 concentration of 2 nM for 24 h. The cells were collected and washed three times with PBS. For protein expression analysis of M1- and M2-releated markers, the cells were stained with PE anti-CD80 (Biolegend, cat. No 104708, clone 16-10A1, 1/50 dilution), PE/Cyanine7 anti-CD86 (Biolegend, cat. No 105116, clone PO3, 1/50 dilution), APC anti-I-A/I-E (MHC II) (Biolegend, cat. No 107613, clone M5/114.15.2, 1/100 dilution) or FITC anti-CD206 (MMR) (Biolegend, cat. No 141703, clone C068C2, 1/500 dilution) for flow cytometric analysis. For the mRNA expression analysis of M1-related (*CD80*, *CD86*, *TNF-α*, *iNOS*, *CXCL9* and *CXCL10*) and M2-releated markers (*Mgl1* and *Mrc1*), the total RNA from cells collected as described above was extracted using the TRIzol (Takara, Frankfurt, Germany), and complementary DNA (cDNA) was synthesized using a PrimeScript RT reagent kit (Takara, Frankfurt, Germany). RT-qPCR was performed using QuantStudio 3 real-time PCR system (ThermoFisher Scientific, USA). The used primer sequences were as follows: mouse *GAPDH* (F: 5′-GTTCCTACCCCCAATGTGTCC-3′, R: 5′-TAGCCCAAGATGCCCTTCAGT-3′); mouse *CD80* (F: 5′- TGCTGCTGATTC GTCTTTCAC-3′, R: 5′- GAGGAGAGTTGTAACGGCAAG-3′); mouse *CD86* (F: 5′-TTGTGTGTGTTCTGGAAACGGAG-3′, R: 5′-AACTTAGAGGCTGTGTTGCT GGG-3′); mouse *TNF-α* (F: 5′- GACGTGGAACTGGCAGAAGAG-3′, R: 5′-TTGG TGGTTTGTGAGTGTGAG-3′); mouse *iNOS* (F: 5′-GATGTTGAACTATGTCCTAT CTCC-3′, S: 5′-GAACACCACTTTCACCAAG AC-3′); mouse *Mgl1* (F: 5′-AGAA AACCCAAGAGCCTGGT-3′, R: 5′-GAGGCC CAGGGAGAACAG-3′); mouse *Mrc1* (F: 5′-ATGGGCAACATCGAGCAGAA-3′, R: 5′-AAACCAATGCAACCCA GTGC-3′); mouse *CXCL9* (F: 5′-TCCTTTTGGGCAT CATCTTCC-3′, R: 5′-TTTGT AGTGGATCGTGCCTCG′); mouse *CXCL10* (F: 5′-CCAAGTGCTGCCGTCAT TTTC-3′, R: 5′-GGCTCGCAGGGATGATTTCAA -3′), which were synthesized by Tsingke Biotechnology Co., Ltd. (Beijing, China).

### Cytotoxicity of reprogrammed M2-like macrophages against tumor cells

IL-4-stimulated RAW264.7 cells were treated with PBS, MPs, M2pep-MPs, MPs_AFP_, M2pep-MPs_AFP_, R848, R848@MPs, R848@M2pep-MPs, R848@MPs_AFP_ or R848@M2pep-MPs_AFP_ at the R848 concentration of 2 nM for 24 h and the supernatants were then collected as conditional media. The conditional media was used to treat Hepa1-6 cells in the presence or absence of Etan (0.5 μg mL^−1^) for 24 h. The cell viability of Hepa1-6 cells was measured by CCK-8 assay according to the manufacturer’s instructions.

### Phagocytosis of tumor cells by reprogrammed M2-like macrophages

IL-4-stimulated RAW264.7 cells were treated with PBS, MPs, M2pep-MPs, MPs_AFP_, M2pep-MPs_AFP_, R848, R848@MPs, R848@M2pep-MPs, R848@MPs_AFP_ or R848@M2pep-MPs_AFP_ at the R848 concentration of 2 nM for 24 h. The treated M2-like macrophages labeled with DiD were co-cultured with DiO-labeled Hepa1-6 cells at a ratio of 1:1 at 37 °C for 4 h. The phagocytosis of Hepa1-6 cells by macrophages was determined as the ratio of the numbers of DiD^+^DiO^+^ cells to DiD^+^ cells by flow cytometry.

### T cell proliferation, activation and cytotoxicity against tumor cells in vitro

Naïve CD8^+^ T cells were isolated from the spleens of healthy C57BL/6 mice using MojoSort™ Mouse CD8 T Cell Isolation Kit (Biolegend, San Diego, CA, USA) according to the manufacturer’s instructions, and then cultured in complete RPMI 1640 medium containing 20 ng mL^−1^ IL-2 for further use. To assess T cell proliferation, fresh CD8^+^ T cells were pre-labeled with 2 μM CFSE. M2-like macrophage were pretreated with PBS, MPs, M2pep-MPs, MPs_AFP_, M2pep-MPs_AFP_, R848, R848@MPs, R848@M2pep-MPs, R848@MPs_AFP_ or R848@M2pep-MPs_AFP_ at the R848 concentration of 2 nM for 24 h. The CFSE-labeled CD8^+^ T cells were co-cultured with the pretreated M2-like macrophages at a ratio of 1:1 for 3 days^60,62^. The cells were collected and then stained with PE/Cyanine7 anti-CD8a (Biolegend, cat. No 100722, clone 53-6.7, 1/80 dilution) for flow cytometric analysis. The CD8^+^ T cell proliferation was indicated by CFSE dilution.

To detect CD8^+^ T cell activation, CD8^+^ T cells were co-cultured with the above pretreated M2-like macrophages at a ratio of 1:1 for 5 days^63^. The cells were collected, treated with 1 mL cell activation Cocktail with brefeldin A (Biolegend, cat. No 423403) at 37 °C for 2 h, stained with PE/Cyanine7 anti-CD8a and then fixed in 0.5 mL Fixation Buffer (Biolegend, cat. No 420801). The cells were resuspended in Intracellular Staining Perm Wash Buffer (Biolegend, cat. No 421002) and then stained with APC anti-IFNγ (Biolegend, cat. No 505810, clone XMG1.2, 1/20 dilution) or PE anti-GzmB (Biolegend, cat. No 372208, clone QA16A02, 1/20 dilution) for flow cytometric analysis. To assess the antigen specific CD8^+^ T cell cytotoxicity against tumor cells, 1 × 10^4^ Hepa1-6 cells or B16-OVA cells (target cells) were cultured in 96-well plates. The above treated CD8^+^ T cells (effector cells) were added at the different effector cells: target cells ratio. After 6 h incubation, the cytotoxic effect of CD8^+^ T cells against tumor cells were determined by LDH assay (Dojindo, Kumamoto, Japan).

### T cell proliferation and activation ex vivo

The orthotopic Hepa1-6 tumor-bearing mice at 10 days after inoculation were intravenously administrated with PBS, MPs, M2pep-MPs, MPs_AFP_, M2pep-MPs_AFP_, R848, R848@MPs, R848@M2pep-MPs, R848@MPs_AFP_ or R848@M2pep-MPs_AFP_ at the R848 concentration of 0.5 mg kg^−1^ every three days for six times. At 24 h after the last administration, single cell suspension of tumor tissues was obtained and stained with FITC anti-CD11b (Biolegend, cat. No 101206, clone M1/70, 1/200 dilution) and APC anti-F4/80 (Biolegend, cat. No 123116, clone BM8, 1/100 dilution). The CD11b^+^F4/80^+^ TAMs were sorted by flow cytometers MoFlo XDP (Beckman Coulter, Fullerton, CA, USA). To assess the T cell proliferation and activation, the CFSE-labeled CD8^+^ T cells isolated from health C57BL/6 mice were co-cultured with the above sorted TAMs at a ratio of 1:1 for five days. The cells were collected and then stained with PE/Cyanine7 anti-CD8a, APC anti-IFNγ or PE anti-GzmB for flow cytometric analysis.

### AFP-specific CD8^+^ T cell cytotoxicity against tumor cells in vivo

The orthotopic Hepa1-6 tumor-bearing mice were intravenously administrated with PBS, R848@M2pep-MPs or R848@M2pep-MPs_AFP_ at the R848 concentration of 0.5 mg kg^−1^ every three days for six times. On day 3 after the last administration, the naive splenocytes from healthy C57BL/6 mice were incubated with 5 μg mL^−1^ AFP_212_ peptide (GSMLNEHVM) for 1.5 h and labeled with high concentration of CFSE (5 μM), or incubated with 5 μg mL^−1^ OVA_257-264_ peptide (SIINFEKL) for 1.5 h and labeled with low concentration of CFSE (0.5 μM). Then 1 × 10^7^ CFSE^high^- and CFSE^low^-labeled splenocytes at a ratio of 1:1 were intravenously injected into Hepa1-6 tumor-bearing mice. After 24 h, the CFSE^high^- and CFSE^low^-labeled splenocytes in spleens were analyzed by flow cytometry. The percentage of antigen-specific lysis was calculated as follows: antigen-specific lysis (%)= [1-(non-transferred control ratio/experimental ratio)] × 100, ratio = %CFSE^low^ (peak): %CFSE^high^ (peak)^60^.

### In vivo antitumor activity

When tumor volume of subcutaneous Hepa1-6 tumor-bearing mice reached about 150–200 mm^3^, or orthotopic Hepa1-6 tumor-bearing mice were inoculated for 10 days, PBS, MPs, M2pep-MPs, MPs_AFP_, M2pep-MPs_AFP_, R848, R848@MPs, R848@M2pep-MPs, R848@MPs_AFP_ or R848@M2pep-MPs_AFP_ at the R848 concentration of 0.5 mg kg^−1^ were intravenously administrated into the mice every three days for six times with or without intraperitoneal injection of anti-PD-1 antibody (5 mg kg^−1^) every 4 days for four times. For the anticancer activity experiment in subcutaneous B16-OVA tumor-bearing mice, PBS, MPs, M2pep-MPs, MPs_OVA_, M2pep-MPs_OVA_, R848, R848@MPs, R848@M2pep-MPs, R848@MPs_OVA_ or R848@M2pep-MPs_OVA_ at the R848 concentration of 0.5 mg kg^−1^ were intravenously administrated into the mice every two days for six times with or without intraperitoneal injection of anti-PD-1 antibody (5 mg kg^−1^) every 4 days for four times when the tumor volume reached about 50 mm^3^. The tumor volume was measured in subcutaneous Hepa1-6 and B16-OVA tumor-bearing mice according to the formula: width^2 ^× length × 0.5. At day 80 after tumor inoculation, the R848@M2pep-MPs_AFP_ and anti-PD-1 antibody-cured subcutaneous Hepa1-6 tumor-bearing mice or naïve mice were re-challenged with 3 × 10^6^ Hepa1-6 cells in the left flank and 5 × 10^5^ B16-OVA cells in the right flank. The tumor volume was calculated every other day and the effect of long-term antitumor immune memory was evaluated. For orthotopic Hepa1-6 tumor-bearing mice, part of the mice were sacrificed at 26 days after tumor inoculation. The tumor-bearing livers were isolated and photographed, and tumors were separated and weighed. The rest of the mice were used for long-term survival analysis.

To evaluate anticancer activity in DEN-induced HCC, DEN-treated B6C3 F_1_ mice were intravenously injected PBS or R848@M2pep-MPs_AFP_ at the R848 concentration of 0.5 mg kg^−1^ every Monday and Thursday for four weeks with or without intraperitoneal injection of anti-PD-1 antibody (5 mg kg^−1^) every Wednesday for four times. At 30 weeks, part of the mice were sacrificed, and liver tissue was collected to count tumor nodule numbers and weight. Some of liver tissues were fixed with 4% paraformaldehyde, sectioned, and stained with H&E to analyze tumor nodules. The rest of the mice were used to monitor long-term survival.

### Tumor immune microenvironment analysis

When Hepa1-6 tumor-bearing mice, DEN-induced autochthonous HCC mice or B16-OVA-bearing mice were sacrificed after treatment, the tumors, spleens or tumor-draining lymph nodes were collected to acquire single cell suspensions. For M1/M2-like TAM analysis, the cells were stained with PerCP/Cyanine5.5 anti-CD11b, Brilliant Violet 421^TM^ anti-F4/80, PE anti-CD80, PE/Cyanine7 anti-CD86, APC anti-I-A/I-E (MHC II) or FITC anti-CD206. For CD8^+^ T cell proliferation analysis, the cells were stained with FITC anti-CD45, PE anti-CD3 (Biolegend, cat. No 100206, clone 17A2, 1/100 dilution), PE/Cyanine7 anti-CD8a and APC anti-Ki-67 (Biolegend, cat. No 652405, clone 16A8, 1/100 dilution). For CD8^+^ T cell activation analysis, the cells were treated with 1 mL cell activation Cocktail with brefeldin A at 37 °C for 2 h, stained with FITC anti-CD45, PerCP/Cyanine5.5 anti-CD3 (Biolegend, cat. No 100218, clone 17A2, 1/20 dilution) and PE/Cyanine7 anti-CD8a, and then fixed in Fixation Buffer and resuspended in Intracellular Staining Perm Wash Buffer for staining with APC anti-IFNγ or PE anti-GzmB. For memory CD8^+^ T cell analysis, the cells were stained with PerCP/Cyanine5.5 anti-CD45 (Biolegend, cat. No 103132, clone 30-F11, 1/80 dilution), APC anti-CD3 (Biolegend, cat. No 100236, clone 17A2, 1/50 dilution), PE/Cyanine7 anti-CD8a, FITC anti-CD44 (Biolegend, cat. No 103005, clone IM7, 1/200 dilution) and PE anti-CD62L (Biolegend, cat. No 104407, clone MEL-14, 1/100 dilution). For exhausted CD8^+^ T cell or OVA-specific exhausted CD8^+^ T cell analysis, the cells were stained with Brilliant Violet 510^TM^ anti-CD45, FITC anti-CD3 (Biolegend, cat. No 100204, clone 17A2, 1/50 dilution), PE/Cyanine7 anti-CD8a, PE-labeled SIINFEKL-H-2Kb tetramer and Brilliant Violet 421^TM^ anti-CD279 (PD-1) (Biolegend, cat. No 135221, clone 29 F.1A12, 1/200 dilution). The cells were then treated with transcription factor buffer set and re-stained with Alexa Fluor® 647 anti-TCF1 (Biolegend, cat. No 655204, clone 7F11A10, 1/20 dilution) and Alexa Fluor® 700 anti-GzmB (Biolegend, cat. No 372222, clone QA16A02, 1/20 dilution). For MDSC analysis, the cells were stained with FITC anti-CD45, PerCP/Cyanine5.5 anti-CD11b and APC anti-Ly-6G/Ly-6C (Gr-1) (Biolegend, cat. No 108412, clone RB6-8C5, 1/100 dilution). For Treg analysis, the cells were firstly stained with FITC anti-CD45, PE/Cyanine7 anti-CD3, PerCP/Cyanine5.5 anti-CD4 and APC anti-CD25. The cells were then treated with transcription factor buffer set and re-stained with Brilliant Violet 421^TM^ anti-FoxP3. All antibodies were used according to the manufacturer’s instructions and incubated with the cells for 30 min in dark at room temperature for flow cytometric analysis. Gating strategies of macrophages and immune cell populations have been described as indicated in Supplementary Fig. 46. Meanwhile, the peripheral blood of orthotopic Hepa1-6 tumor-bearing mice after treatment was collected, centrifuged at 1,500 *g* at 4 °C for 10 min and the contents of TNF-α and IFNγ were determined using ELISA kits according to manufacturer’s instruction (DAKEWE, China).

### T cell recruitment

T cell recruitment assay was performed using 5-μm transwell filter (Corning Costar). IL-4-stimulated RAW264.7 cells were treated with PBS, MPs, M2pep-MPs, MPs_AFP_, M2pep-MPs_AFP_, R848, R848@MPs, R848@M2pep-MPs, R848@MPs_AFP_ or R848@M2pep-MPs_AFP_ at the R848 concentration of 2 nM for 24 h. 600 μL of the supernatants of the above cells in the presence or absence of Etan (0.5 μg mL^−1^) was added into the bottom chambers and T lymphocytes isolated from the spleens of healthy C57BL/6 were seeded in the top chambers. After 6 h incubation, the CD45^+^CD3^+^CD8^+^ T cells in bottom chambers were detected by flow cytometry.

### Antigen-specific immunological memory

For ELISPOT evaluation, 5 × 10^5^ cells splenocytes isolated from the orthotopic Hepa1-6 tumor bearing mice after treatment were seeded into the wells of ELISPOT plates (Biolegend, San Diego, CA, USA) and incubated with 10 µg mL^−1^ AFP_212_ or OVA_257-264_ peptide at 37 °C in 5% CO_2_ for 24 h. The cells were lysed and the amounts of IFNγ spot-forming cells were determined by an automated ELISPOT Plate Reader (ASTORTM, Mabtech, Sweden).

To evaluate the antigen-specific cytotoxicity, 1 × 10^7^ splenocytes isolated from the orthotopic Hepa1-6 tumor bearing mice after treatment were seeded in 6-well plates, incubated with 10 µg mL^−1^ AFP_212_ or OVA_257-264_ at 37 °C in 5% CO_2_ for 5 days and then isolated CD8^+^ T cells using MojoSort™ mouse CD8 T cell isolation kit (Biolegend, San Diego, CA, USA). 1 × 10^4^ Hepa1-6 cells and 1 × 10^5^ isolated CD8^+^ T cells were co-incubated for 6 h and the cytotoxicity of CD8^+^ T cells against Hepa1-6 cells was determined by LDH assay.

### Statistical analysis

Data are presented as means ± sd except that tumor volumes are presented as means ± sem. Comparison between two groups was performed using unpaired two-tailed Student’s *t*-test. For comparison of multiple groups, one-way ANOVA or two-way ANOVA was used followed by Tukey’s honest significant difference (HSD) post-hoc test or Bonferroni’s multiple comparisons post-test, except where otherwise noted. Statistical analysis was performed using GraphPad Prism 7.0 (GraphPad Software, CA) software. *P* < 0.05 was considered statistically significant.

### Reporting summary

Further information on research design is available in the Nature Portfolio Reporting Summary linked to this article.

### Supplementary information

Supplementary Information

Reporting Summary

### Source data

Source Data

## Data Availability

The tumor RNA-seq data generated in this study have been deposited in the GEO database under accession code GSE224237. The remaining data supporting the results of this study are available within the Article, Supplementary Information, or Source Data File. Source data are provided in this paper.
