# Supplementary material for: Cell microparticles loaded with tumor antigen and resiquimod reprogram tumor-associated macrophages and promote stem-like CD8+ T cells to boost anti-PD-1 therapy
Source: Nat Commun. 2023 Sep 13;14:5653. doi: 10.1038/s41467-023-41438-9 (PMC10499806; doi:10.1038/s41467-023-41438-9)
Supplement: Supplementary file 1 — Supplementary Information [file 41467_2023_41438_MOESM1_ESM.pdf]

Supplementary Information

Cell microparticles loaded with tumor antigen and resiquimod reprogram tumor-associated macrophages and promote stem-like CD8$^+$ T cells to boost anti-PD-1 therapy

Xiaoqiong Zhang[1,#], Zhaohan Wei[1,#], Tuying Yong[1,2,3,4,#], Shiyu Li[1], Nana Bie[1], Jianye Li[1], Xin Li[1], Haojie Liu[1], Hang Xu[2], Yuchen Yan[1], Bixiang Zhang[5], Xiaoping Chen[5], Xiangliang Yang[1,2,3,4,*] and Lu Gan[1,2,3,4,*]

[1]National Engineering Research Center for Nanomedicine, College of Life Science and Technology, Huazhong University of Science and Technology, Wuhan 430074, China

[2]Key Laboratory of Molecular Biophysics of the Ministry of Education, College of Life Science and Technology, Huazhong University of Science and Technology, Wuhan 430074, China

[3]Hubei Key Laboratory of Bioinorganic Chemistry and Materia Medica, Huazhong University of Science and Technology, Wuhan 430074, China

[4]Hubei Bioinformatics and Molecular Imaging Key Laboratory, Huazhong University of Science and Technology, Wuhan 430074, China

[5]Hepatic Surgery Center, Tongji Hospital, Tongji Medical College, Huazhong University of Science and Technology, Wuhan 430030, China.

[#]Xiaoqiong Zhang, Zhaohan Wei and Tuying Yong contributed equally to this work.

*Correspondence should be addressed to: lugan@mail.hust.edu.cn, yangxl@mail.hust.edu.cn.

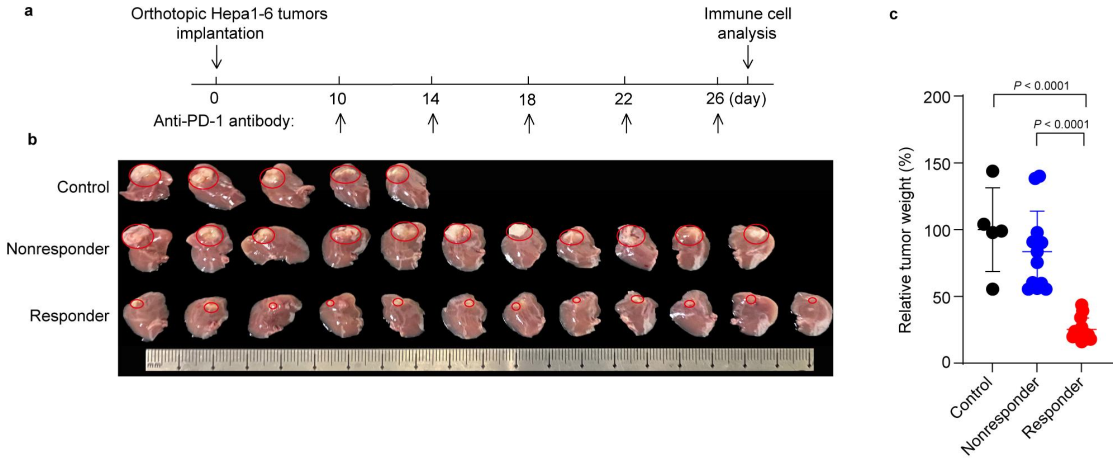

Supplementary Fig. 1 Anticancer activity of anti-PD-1 antibody in orthotopic Hepa1-6 tumor-bearing mice. (**a**) Schematic schedule for tumor inhibition and immune cell analysis experiment in orthotopic Hepa1-6 tumor-bearing mice after intraperitoneal injection of anti-PD-1 antibody at the dosage of 5 mg kg^{-1}. (**b,c**) Tumor image (**b**) and tumor weight (**c**) of PBS-, anti-PD-1 antibody-responsive and anti-PD-1 antibody-nonresponsive mice after treatment indicated in (**a**)**.** Data are presented as means ± s.d. (n = 5 mice for PBS-treated group, n = 11 mice for nonresponsive group, n = 12 mice for responsive group; one-way ANOVA followed by Tukey's HSD post-hoc test). Source data are provided as a Source Data file.

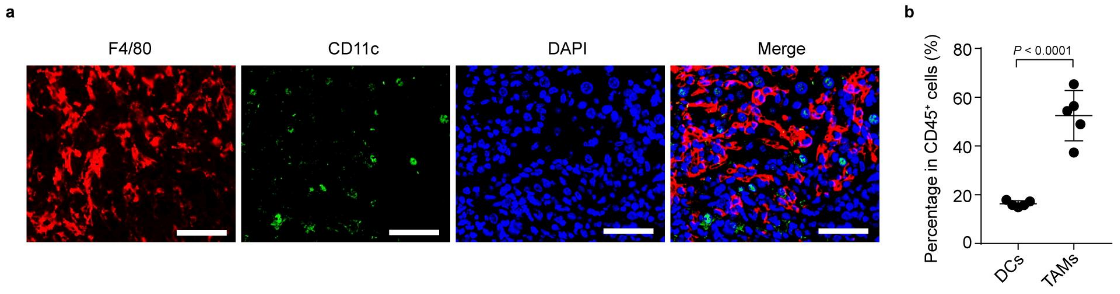

Supplementary Fig. 2 Distribution of DCs and TAMs in tumors of orthotopic Hepa1-6 tumor-bearing mice. (**a**) Immunofluorescence images of F4/80 (TAMs marker) and CD11c (DCs marker) in the tumor tissues of orthotopic Hepa1-6 tumor-bearing mice at 27 days after tumor inoculation. Scale bars: 50 μm. Images are representative of three independent samples. (**b**) The percentages of DCs (gated as $CD45^{+}F4/80^{-}CD11c^{+}$) and TAMs (gated as $CD45^{+}CD11b^{+}F4/80^{+}$) in $CD45^{+}$ cells in the tumor tissues of orthotopic Hepa1-6 tumor-bearing mice at 27 days after tumor inoculation by flow cytometry. Data are presented as means ± s.d. (n = 5 mice per group; unpaired two-tailed Student's *t*-test). Source data are provided as a Source Data file.

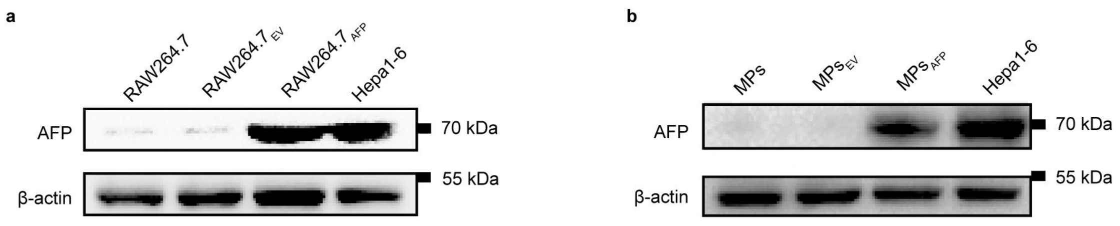

Supplementary Fig. 3 AFP expression in RAW264.7$_{AFP}$ cells and MPs$_{AFP}$. (a) AFP expression in RAW264.7 cells, RAW264.7 cells stably transfected with empty vector (RAW264.7$_{EV}$) and RAW264.7$_{AFP}$ cells by western blotting. (**b)** AFP expression in MPs, MPs$_{EV}$ and MPs$_{AFP}$ by western blotting. Hepa1-6 cells were used as a positive control. Images are representative of three independent samples.

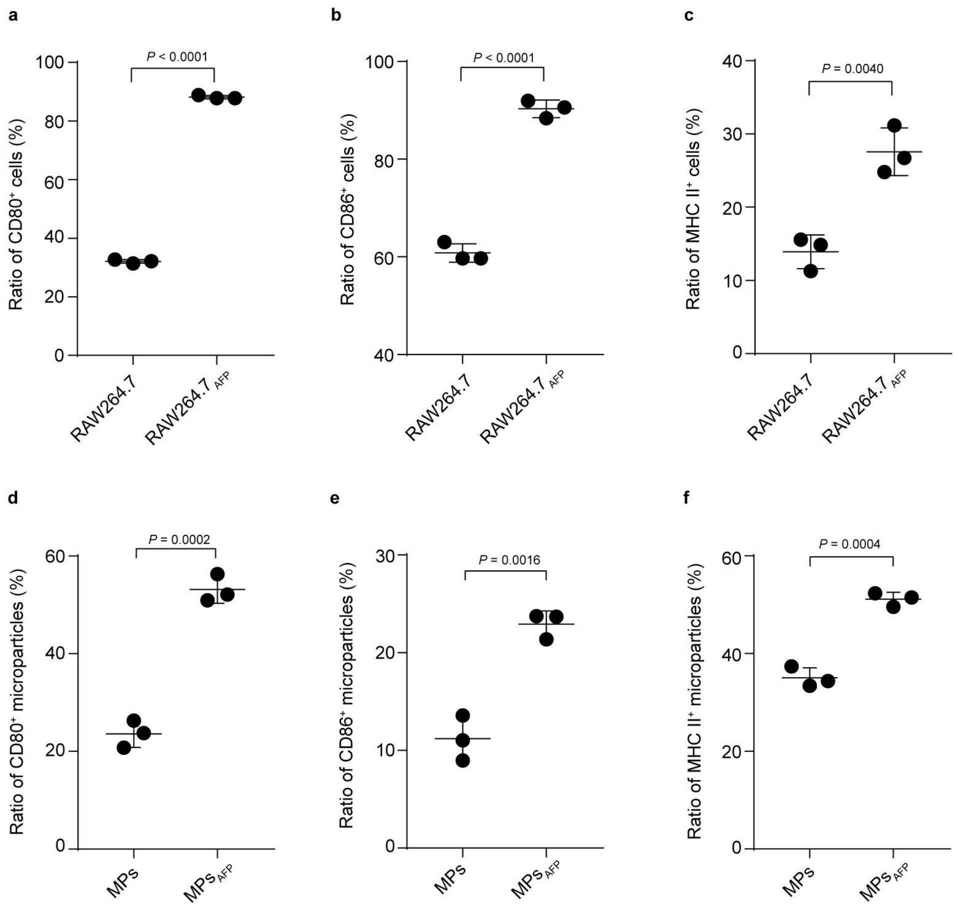

Supplementary Fig. 4 Effects of AFP overexpression on RAW264.7 cells. (**a-c**) Percentages of $CD80^+$ (**a**), $CD86^+$ (**b**) and MHC II^+ (**c**) cells in RAW264.7 and RAW264.7$_{AFP}$ cells. Data are presented as means ± s.d. (n = 3 biological independent samples; unpaired two-tailed Student's *t*-test). (**d-f**) Percentages of $CD80^+$ (**d**), $CD86^+$ (**e**) and MHC II^+ (**f**) MPs derived from RAW264.7 and RAW264.7$_{AFP}$ cells. Data are presented as means ± s.d. (n = 3 biological independent samples; unpaired two-tailed Student's *t*-test). Source data are provided as a Source Data file.

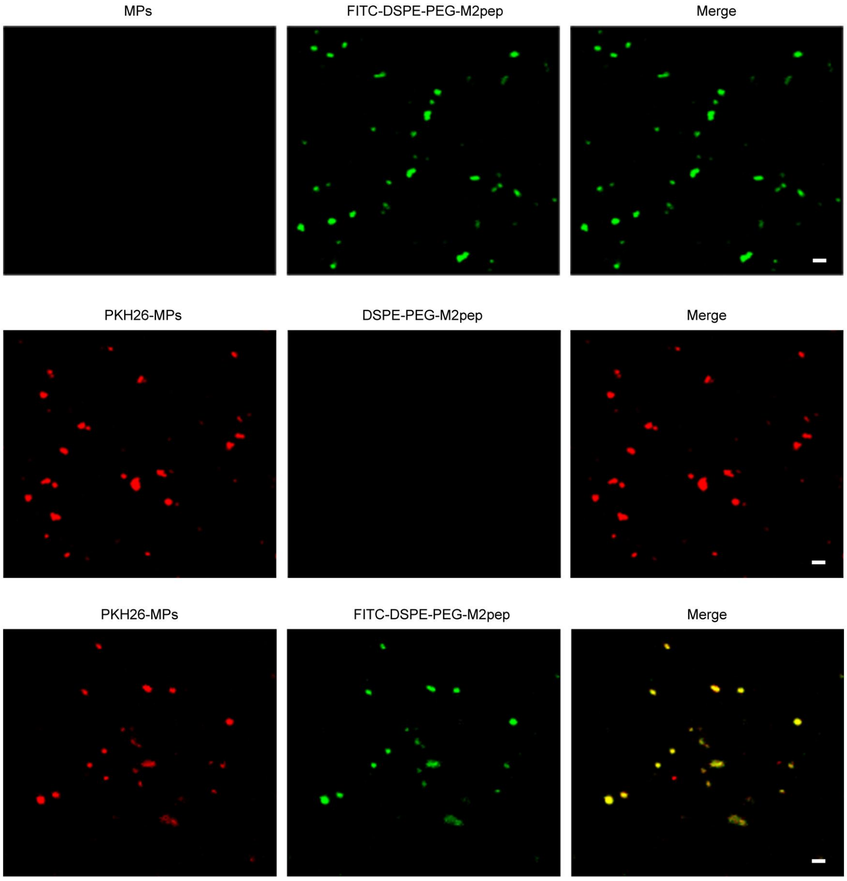

Supplementary Fig. 5 Colocalization of M2pep and MPs in M2pep-MPs by confocal microscopy. M2pep-MPs were prepared by incubating PKH26-labeled MPs with FITC-labeled DSPE-PEG-M2pep at a mass ratio of 50:1 at 4 °C for 24 h and then observed by confocal microcopy. Scale bars: 1 μm. Images are representative of three independent samples.

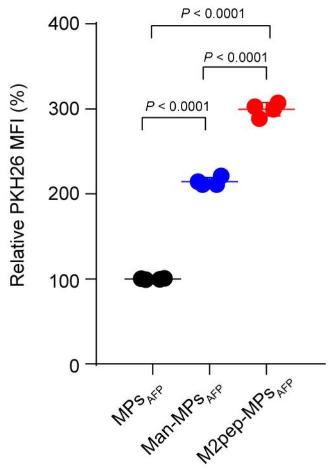

Supplementary Fig. 6 Targeting capacity of Man-MPs$_{AFP}$ and M2pep-MPs$_{AFP}$ to M2-like macrophages. IL-4-stimulated RAW264.7 cells (M2-like macrophages) were treated with PKH26-labeled MPs$_{AFP}$, Man-MPs$_{AFP}$ or M2pep-MPs$_{AFP}$ at the concentration of 10 μg protein mL^{-1} for 4 h, and PKH26 MFI was determined by flow cytometry. Data are presented as means ± s.d. (n = 4 biological independent samples; one-way ANOVA followed by Tukey's HSD post-hoc test). Source data are provided as a Source Data file.

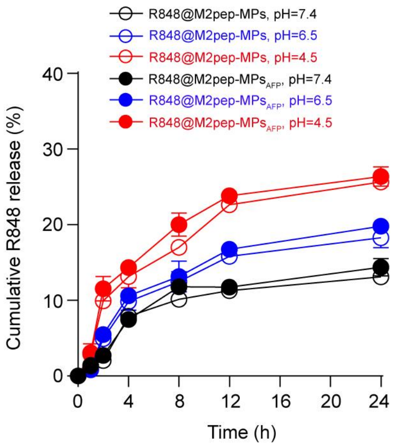

Supplementary Fig. 7 In vitro pH-dependent drug release of R848@M2pep-MPs$_{AFP}$. R848@M2pep-MPs and R848@M2pep-MPs$_{AFP}$ were put in PBS at different pH values for different time intervals, and the released R848 content was determined by HPLC. Data are presented as means ± s.d. (n = 3 independent samples). Source data are provided as a Source Data file.

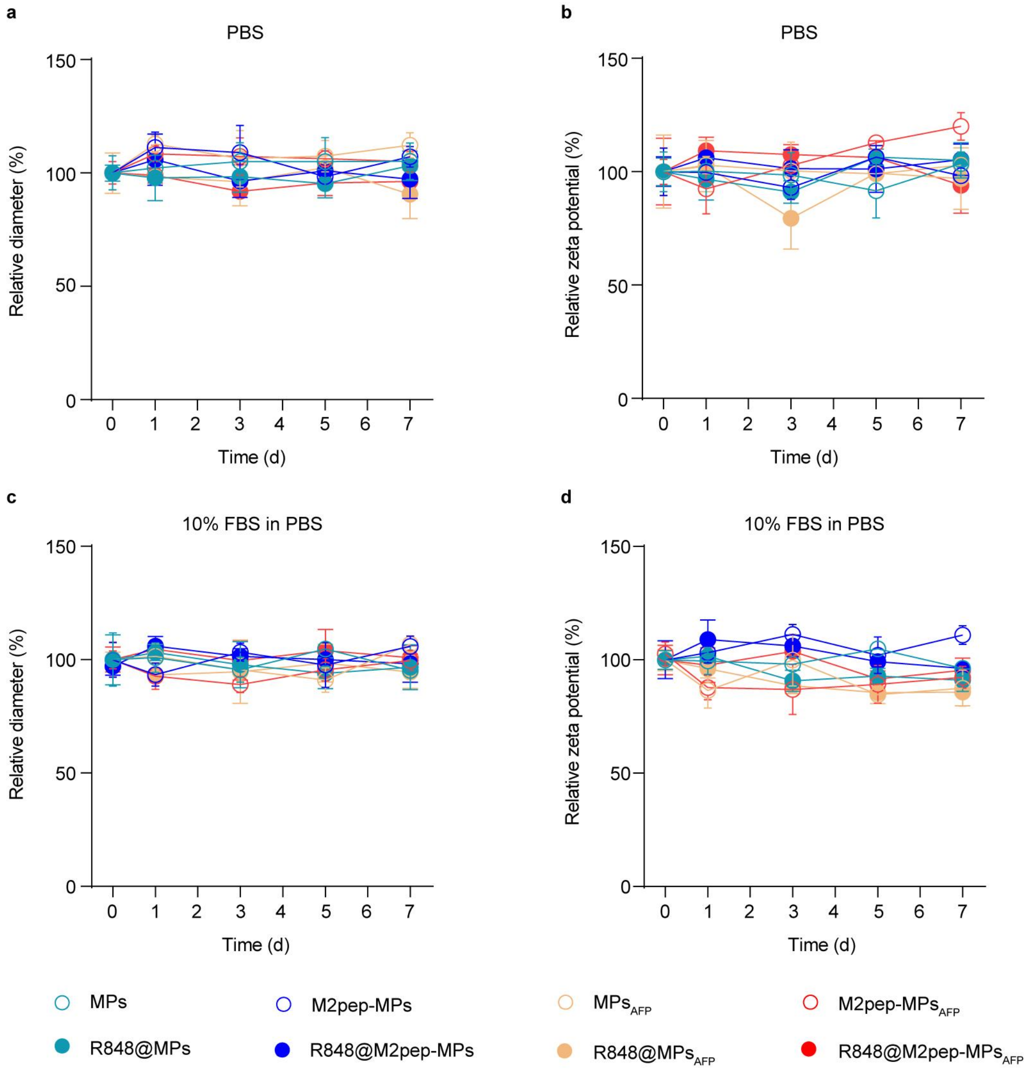

Supplementary Fig. 8 Stability of R848@M2pep-MPs$_{AFP}$. (a,b) Relative diameter (**a**) and zeta potential (**b**) of MPs, M2pep-MPs, MPs$_{AFP}$ and M2pep-MPs$_{AFP}$ with or without R848 after incubation in PBS for different time intervals. Data are presented as means ± s.d. (n = 3 independent samples). **(c,d)** Relative diameter (**c**) and zeta potential (**d**) of MPs, M2pep-MPs, MPs$_{AFP}$ or M2pep-MPs$_{AFP}$ with or without R848 after incubation in PBS containing 10% FBS for different time intervals. Data are presented as means ± s.d. (n = 3 independent samples). Source data are provided as a Source Data file.

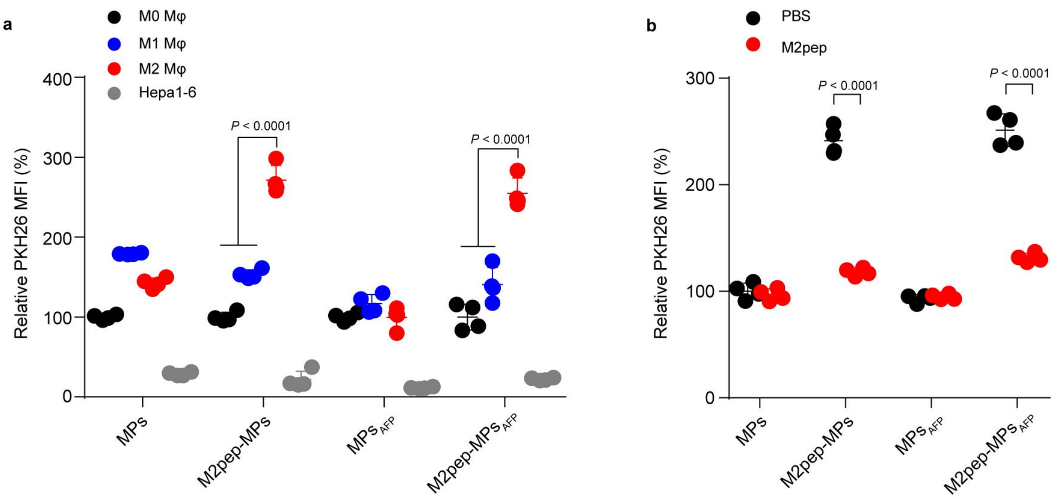

Supplementary Fig. 9 M2-like macrophage-targeting capacity of M2pep-MPs$_{AFP}$. (**a**) Relative PKH26 MFI in RAW264.7 cells (M0 macrophages), LPS- and IFNγ-stimulated RAW264.7 cells (M1-like macrophages), IL-4-stimulated RAW264.7 cells (M2-like macrophages) and Hepa1-6 cells after treatment with PKH26-labeled MPs, M2pep-MPs, MPs$_{AFP}$ or M2pep-MPs$_{AFP}$ at the concentration of 10 μg protein mL^{-1} for 4 h by flow cytometry. Data are presented as means ± s.d. (n = 4 biological independent samples; one-way ANOVA followed by Tukey's HSD post-hoc test). (**b**) Relative PKH26 MFI in M2-like macrophages after treatment with PKH26-labeled MPs, M2pep-MPs, MPs$_{AFP}$ or M2pep-MPs$_{AFP}$ at the concentration of 10 μg protein mL^{-1} in the presence or absence of 5 μg mL^{-1} M2pep for 4 h. Data are presented as means ± s.d. (n = 4 biological independent samples; unpaired two-tailed Student's *t*-test). Source data are provided as a Source Data file.

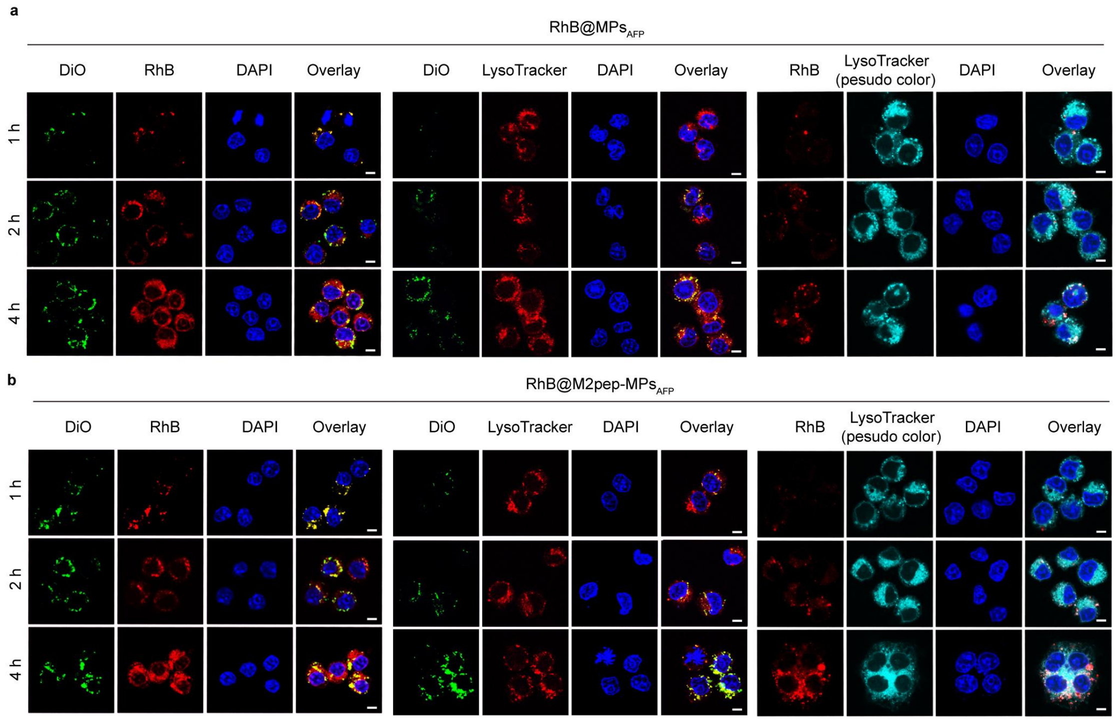

Supplementary Fig. 10 Intracellular trafficking of RhB@MPs$_{AFP}$ and RhB@M2pep-MPs$_{AFP}$ in M2-like macrophages. (a,b) Confocal microscopic images of M2-like macrophages after treatment with RhB-loaded DiO-labeled MPs$_{AFP}$ (**a**) or M2pep-MPs$_{AFP}$ (**b**) at the concentration of 10 μg protein mL^{-1} for different time intervals and then labeling with 75 nM LysoTracker® Deep Red (lysosomes labeling dye) or 1 μg mL^{-1} DAPI (nucleus labeling dye), respectively. Scale bars: 5 μm. Images are representative of three independent samples.

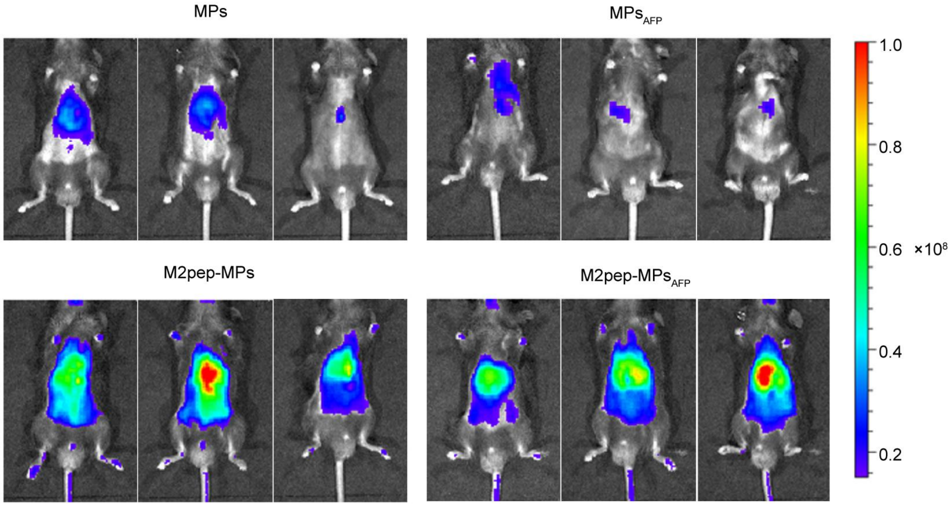

Supplementary Fig. 11 In vivo biodistribution of M2pep-MPs$_{AFP}$. Hepa1-6 tumor-bearing mice were intravenously injected with IR780-labeled MPs, M2pep-MPs, MPs$_{AFP}$ or M2pep-MPs$_{AFP}$ at the dosage of 15 mg protein kg^{-1}. At 24 h after injection, the in vivo imaging of mice was performed. (n = 3 mice per group).

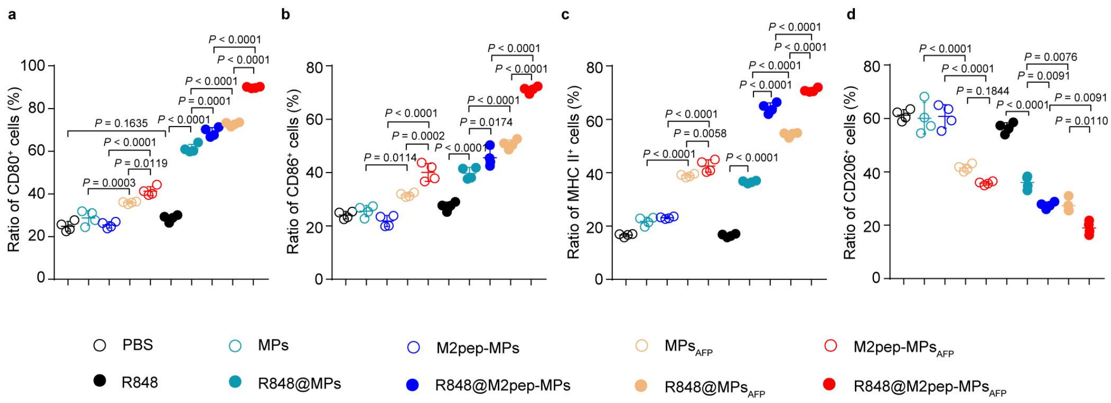

Supplementary Fig. 12 Reprogramming M2-like macrophages into M1-like phenotype by R848@M2pep-MPs$_{AFP}$. (**a-d**) Ratios of CD80$^+$ (**a**), CD86$^+$ (**b**), MHC II$^+$ (**c**) and CD206$^+$ (**d**) cells in IL-4-stimulated RAW264.7 cells after treatment with PBS, MPs, M2pep-MPs, MPs$_{AFP}$, M2pep-MPs$_{AFP}$, R848, R848@MPs, R848@M2pep-MPs, R848@MPs$_{AFP}$ or R848@M2pep-MPs$_{AFP}$ at the R848 concentration of 2 nM for 24 h by flow cytometry. Data are presented as means ± s.d. (n = 4 biological independent samples; one-way ANOVA followed by Tukey's HSD post-hoc test). Source data are provided as a Source Data file.

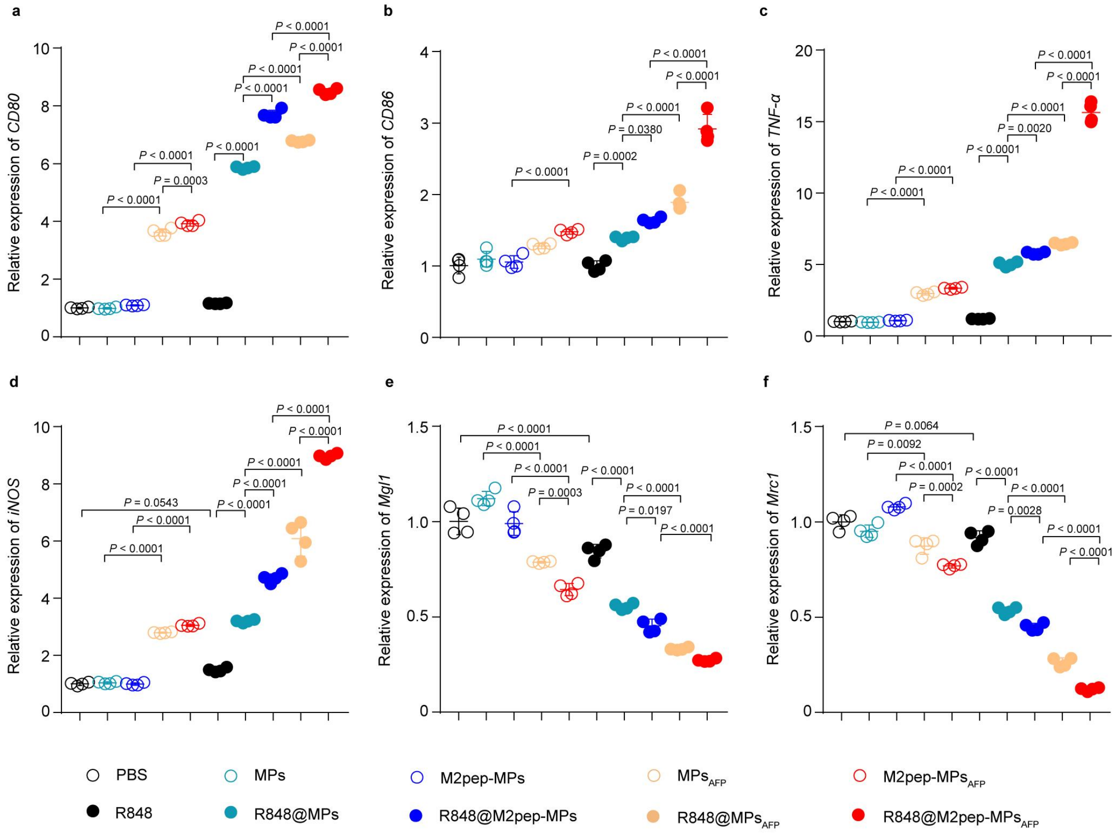

Supplementary Fig. 13 Reprogramming of M2-like macrophages to M1-like phenotype by R848@M2pep-MPs$_{AFP}$. **(a-f)** The mRNA expression levels of *CD80* (**a**), *CD86* (**b**), *TNF-α* (**c**), *iNOS* (**d**), *Mgl1* (**e**) and *Mrc1* (**f**) in IL-4-stimulated RAW264.7 cells after treatment with PBS, MPs, M2pep-MPs, MPs$_{AFP}$, M2pep-MPs$_{AFP}$, R848, R848@MPs, R848@M2pep-MPs, R848@MPs$_{AFP}$ or R848@M2pep-MPs$_{AFP}$ at the R848 concentration of 2 nM for 24 h by RT-qPCR. Data are presented as means ± s.d. (n = 4 biological independent samples; one-way ANOVA followed by Tukey's HSD post-hoc test). Source data are provided as a Source Data file.

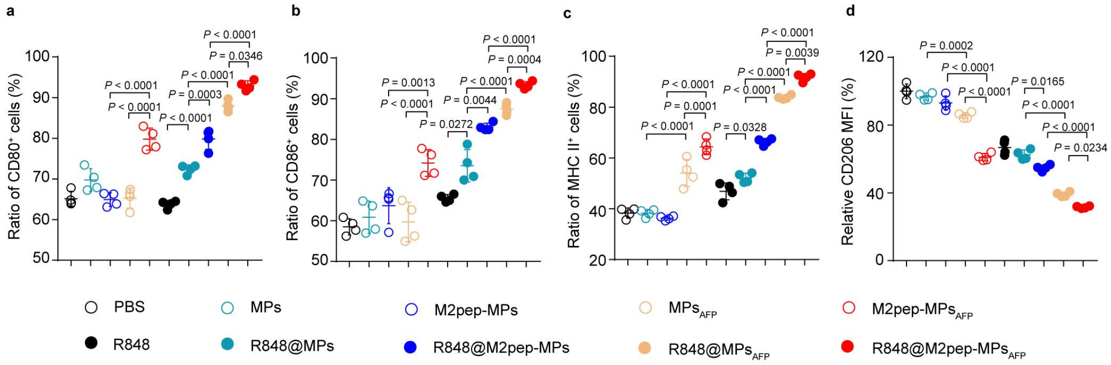

Supplementary Fig. 14 Reprogramming of M2-like BMDMs to M1-like phenotype by R848@M2pep-MPs$_{AFP}$. (**a-d**) Percentage of CD80^{+} (**a**), CD86^{+} (**b**) and MHC II^{+} (**c**) cells and relative CD206 MFI (**d**) in IL-4-stimulated BMDMs after treatment with PBS, MPs, M2pep-MPs, MPs$_{AFP}$, M2pep-MPs$_{AFP}$, R848, R848@MPs, R848@M2pep-MPs, R848@MPs$_{AFP}$ or R848@M2pep-MPs$_{AFP}$ derived from RAW264.7 or RAW264.7$_{AFP}$ cells at the R848 concentration of 2 nM for 24 h by flow cytometry. Data are presented as means ± s.d. (n = 4 biological independent samples; one-way ANOVA followed by Tukey's HSD post-hoc test). Source data are provided as a Source Data file.

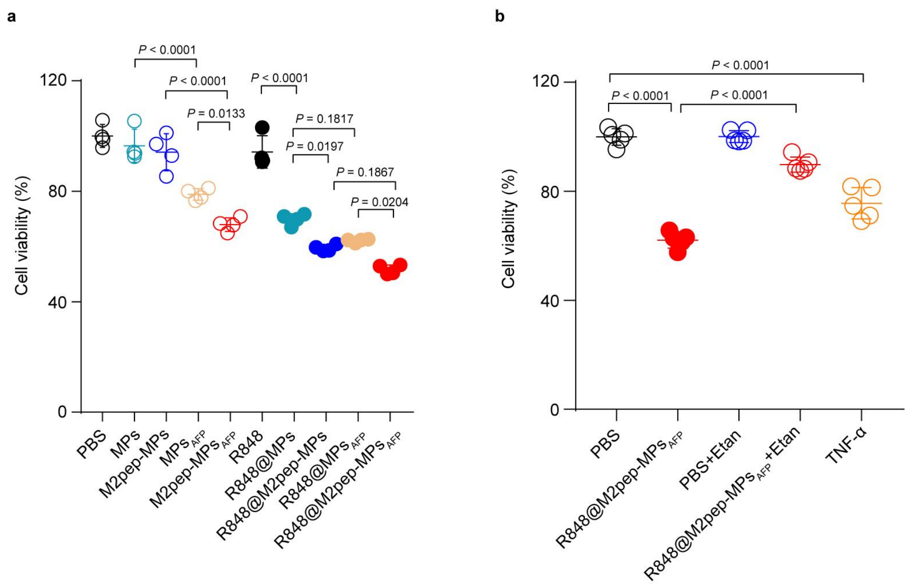

Supplementary Fig. 15 TNF-α involved in the cytotoxicity of R848@M2pep-MPs$_{AFP}$-reprogrammed M2-like macrophages against Hepa1-6 cells. (**a**) Cell viability of Hepa1-6 cells at 24 h after incubation with the supernatants of IL-4-stimulated RAW264.7 cells pretreated with PBS, MPs, M2pep-MPs, MPs$_{AFP}$, M2pep-MPs$_{AFP}$, R848, R848@MPs, R848@M2pep-MPs, R848@MPs$_{AFP}$ or R848@M2pep-MPs$_{AFP}$ at the R848 concentration of 2 nM for 24 h by CCK-8 assay. Data are presented as means ± s.d. (n = 4 biological independent samples; one-way ANOVA followed by Tukey's HSD post-hoc test). (**b**) Cell viability of Hepa1-6 cells at 24 h after incubation with the supernatants of IL-4-stimulated RAW264.7 cells pretreated with PBS or R848@M2pep-MPs$_{AFP}$ at the R848 concentration of 2 nM for 24 h in the presence or absence of 0.5 μg mL^{-1} Etan. TNF-α at 0.5 μg mL^{-1} was used as a positive control. Data are presented as means ± s.d. (n = 5 biological independent samples; one-way ANOVA followed by Tukey's HSD post-hoc test). Source data are provided as a Source Data file.

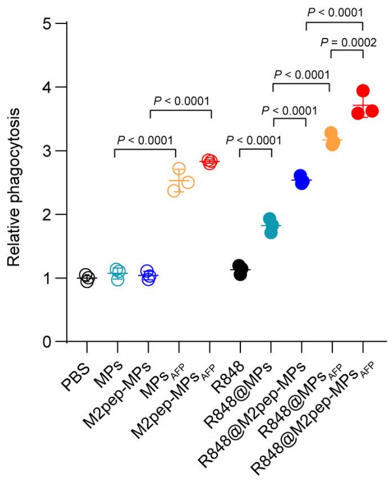

Supplementary Fig. 16 Phagocytosis of Hepa1-6 cells by R848@M2pep-MPs$_{AFP}$-reprogrammed M2-like macrophages. IL-4-stimulated DiD-labeled RAW264.7 cells were treated with PBS, MPs, M2pep-MPs, MPs$_{AFP}$, M2pep-MPs$_{AFP}$, R848, R848@MPs, R848@M2pep-MPs, R848@MPs$_{AFP}$ or R848@M2pep-MPs$_{AFP}$ at the R848 concentration of 2 nM for 24 h, and then co-cultured with DiO-labeled Hepa1-6 cells at a ratio of 1:1 at 37 °C for 4 h. The phagocytosis of tumor cells by macrophages was determined by flow cytometry. Data are presented as means ± s.d. (n = 3 biological independent samples; one-way ANOVA followed by Tukey's HSD post-hoc test). Source data are provided as a Source Data file.

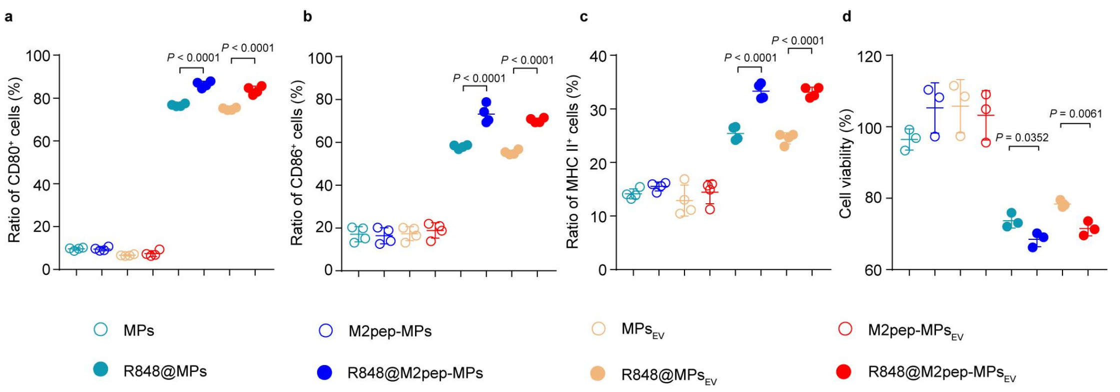

Supplementary Fig. 17 Effects of lentivirus transfection on the reprogramming of M2-like macrophages by R848@M2pep-MPs$_{AFP}$. (**a-c**) Percentage of CD80$^+$ (**a**), CD86$^+$ (**b**) and MHC II$^+$ (**c**) cells in IL-4-stimulated RAW264.7 cells after treatment with MPs, M2pep-MPs, MPs$_{EV}$, M2pep-MPs$_{EV}$, R848@MPs, R848@M2pep-MPs, R848@MPs$_{EV}$ or R848@M2pep-MPs$_{EV}$ at the R848 concentration of 2 nM for 24 h by flow cytometry. Data are presented as means ± s.d. (n = 4 biological independent samples; one-way ANOVA followed by Tukey's HSD post-hoc test). (**d**) Cell viability of Hepa1-6 cells at 24 h after treatment with the supernatants of IL-4-stimulated RAW264.7 cells pretreated with MPs, M2pep-MPs, MPs$_{EV}$, M2pep-MPs$_{EV}$, R848@MPs, R848@M2pep-MPs, R848@MPs$_{EV}$ or R848@M2pep-MPs$_{EV}$ at the R848 concentration of 2 nM for 24 h. Data are presented as means ± s.d. (n = 3 biological independent samples; one-way ANOVA followed by Tukey's HSD post-hoc test). Source data are provided as a Source Data file.

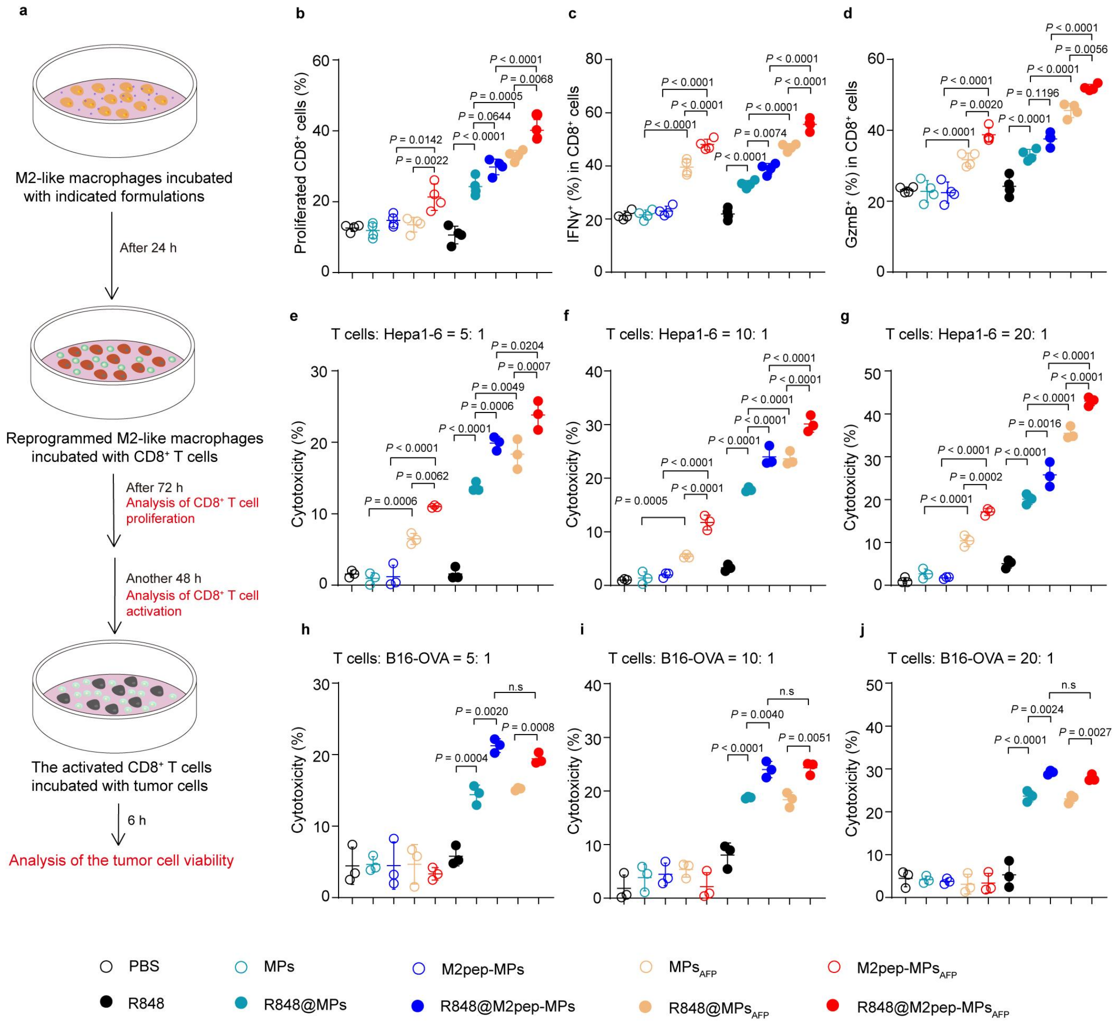

Supplementary Fig. 18 Efficient activation of CD8⁺ T cells by R848@M2pep-MPs$_{AFP}$-reprogrammed M2-like macrophages. (a) Schematic schedule for activating CD8⁺ T cells by R848@M2pep-MPs$_{AFP}$-reprogrammed M2-like macrophages. (**b**) Ratios of proliferated CD8⁺ T cells indicated by CFSE dilution at 3 days after co-culture with the PBS-, MPs-, M2pep-MPs-, MPs$_{AFP}$-, M2pep-MPs$_{AFP}$-, R848-, R848@MPs-, R848@M2pep-MPs-, R848@MPs$_{AFP}$- or R848@M2pep-MPs$_{AFP}$-pretreated IL-4-stimulated RAW264.7 cells (R848 concentration at 2 nM for 24 h) as indicated in (**a**). Data are presented as means ± s.d. (n = 4 biological independent samples; one-way ANOVA followed by Tukey's HSD post-hoc test). (**c,d**) Ratios of IFNγ⁺ (**c**) and GzmB⁺ (**d**) cells in CD8⁺ T cells at 5 days after co-culture with the above-treated IL-4-stimulated RAW264.7 cells as indicated

in (**a**). Data are presented as means ± s.d. (n = 4 biological independent samples; one-way ANOVA followed by Tukey's HSD post-hoc test). (**e-j**) Cytotoxicity of $CD8^+$ T cells against Hepa1-6 or B16-OVA cells when the $CD8^+$ T cells (effector cells) co-cultured with the above-treated IL-4-stimulated RAW264.7 cells for 5 days were incubated with Hepa1-6 or B16-OVA cells (target cells) at the effector/target ratio of 5:1 (**e,h**), 10:1 (**f,i**) and 20:1 (**g,j**) for 6 h as indicated in (**a**) by LDH assay. Data are presented as means ± s.d. (n = 3 biological independent samples; one-way ANOVA followed by Tukey's HSD post-hoc test). Source data are provided as a Source Data file.

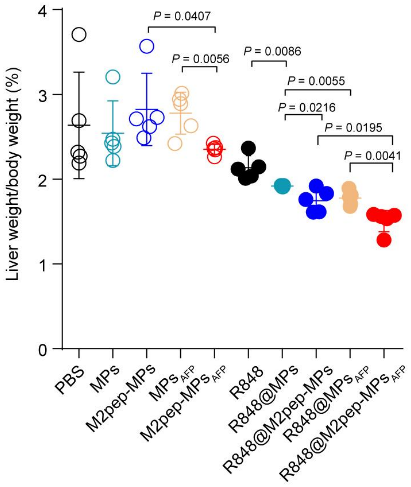

Supplementary Fig. 19 Efficient inhibition in the ratio of liver to body weight after treatment with R848@M2pep-MPs$_{AFP}$ in orthotopic Hepa1-6 tumor-bearing mice. Orthotopic Hepa1-6 tumor-bearing mice were intravenously injected with PBS, MPs, M2pep-MPs, MPs$_{AFP}$, M2pep-MPs$_{AFP}$, R848, R848@MPs, R848@M2pep-MPs, R848@MPs$_{AFP}$ or R848@M2pep-MPs$_{AFP}$ at the R848 dosage of 0.5 mg kg^{-1} every three days for six times. At 16 days after intravenous injection, the ratio of liver to body weight was determined. Data are presented as means ± s.d. (n = 5 mice per group; one-way ANOVA followed by Tukey's HSD post-hoc test). Source data are provided as a Source Data file.

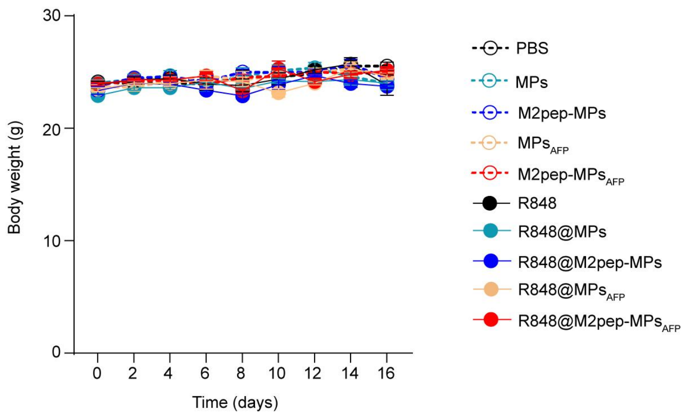

Supplementary Fig. 20 Body weight of orthotopic Hepa1-6 tumor-bearing mice after treatment with R848@M2pep-MPs$_{AFP}$. Orthotopic Hepa1-6 tumor-bearing mice were intravenously injected with PBS, MPs, M2pep-MPs, MPs$_{AFP}$, M2pep-MPs$_{AFP}$, R848, R848@MPs, R848@M2pep-MPs, R848@MPs$_{AFP}$ or R848@M2pep-MPs$_{AFP}$ at the R848 dosage of 0.5 mg kg^{-1} every three days for six times, and the body weight was measured every other day. Data are presented as means ± s.d. (n = 5 mice per group). Source data are provided as a Source Data file.

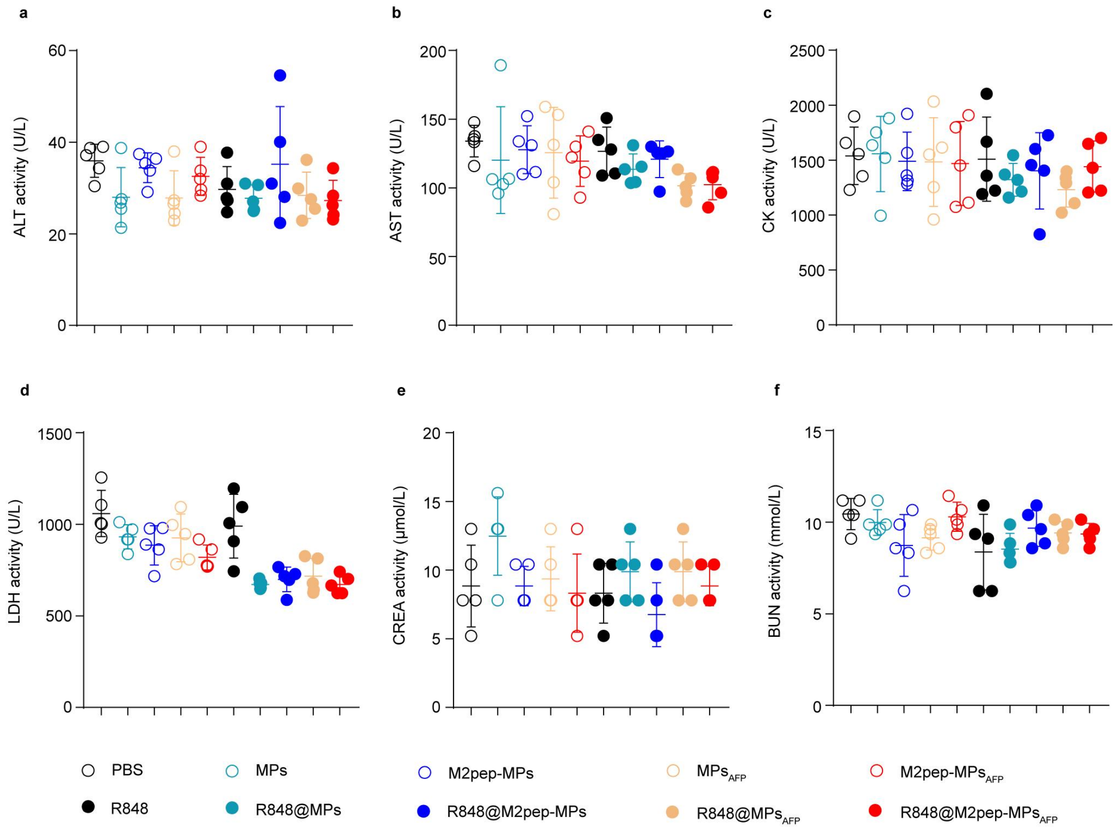

Supplementary Fig. 21 Serological analysis of orthotopic Hepa1-6 tumor-bearing mice after treatment with R848@M2pep-MPs$_{\mathbf{AFP}}$. (**a-e**) The serological analysis of alanine aminotransferase (ALT, **a**), aspartate aminotransferase (AST, **b**), creatine kinase (CK, **c**), lactate dehydrogenase (LDH, **d**), creatinine (CREA, **e**) and blood urea nitrogen (BUN, **f**) in orthotopic Hepa1-6 tumor-bearing mice after intravenous injection of PBS, MPs, M2pep-MPs, MPs$_{AFP}$, M2pep-MPs$_{AFP}$, R848, R848@MPs, R848@M2pep-MPs, R848@MPs$_{AFP}$ or R848@M2pep-MPs$_{AFP}$ at the R848 dosage of 0.5 mg kg^{-1} every three days for six times. Data are presented as means ± s.d. (n = 5 mice per group). Source data are provided as a Source Data file.

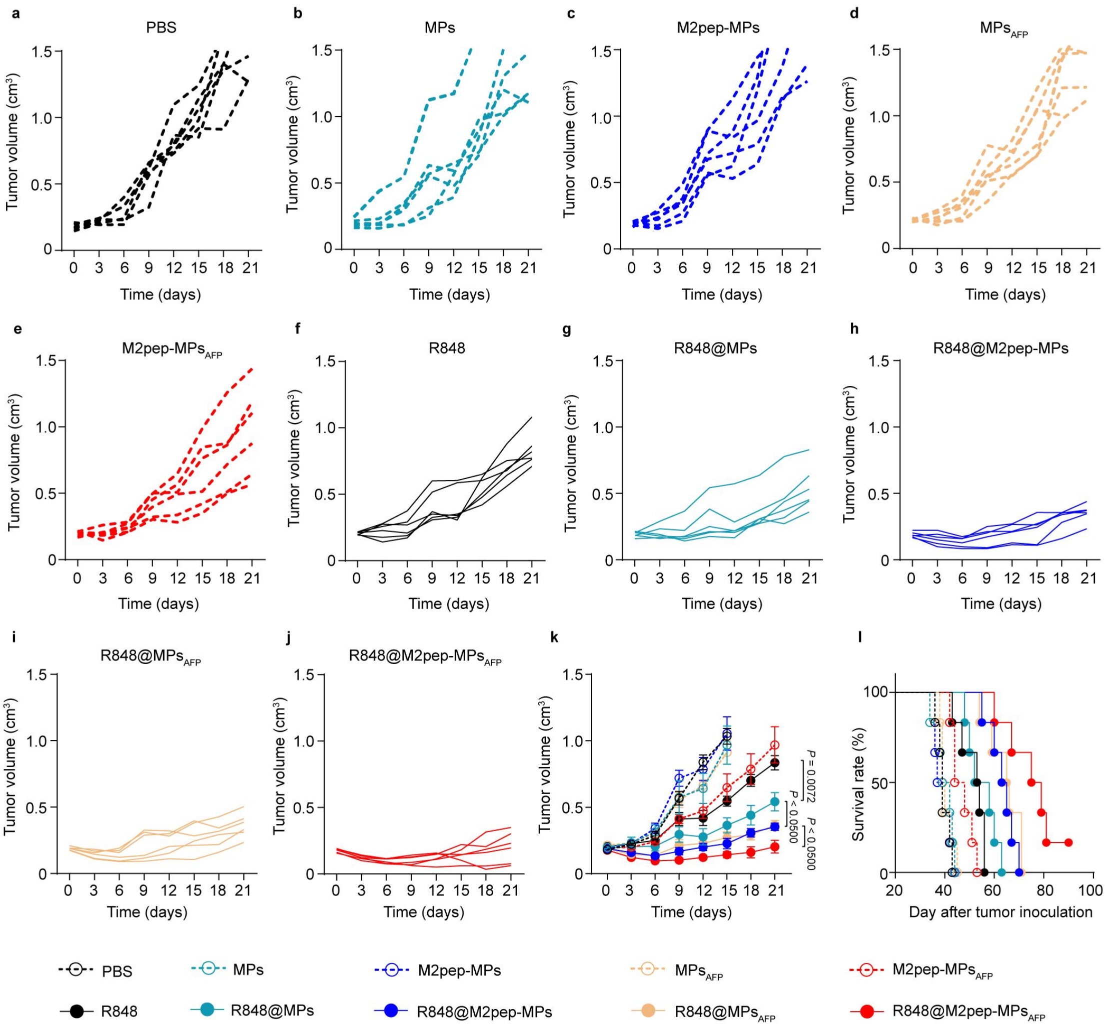

Supplementary Fig. 22 Anticancer activity of R848@M2pep-MPs$_{AFP}$ in subcutaneous Hepa1-6 tumor-bearing mice. (**a-j**) Individual tumor growth curves of subcutaneous Hepa1-6 tumor-bearing mice after intravenous injection of PBS (**a**), MPs (**b**), M2pep-MPs (**c**), MPs$_{AFP}$ (**d**), M2pep-MPs$_{AFP}$ (**e**), R848 (**f**), R848@MPs (**g**), R848@M2pep-MPs (**h**), R848@MPs$_{AFP}$ (**i**) or R848@M2pep-MPs$_{AFP}$ (**j**) at the R848 dosage of 0.5 mg kg^{-1} every three days for six times. (**k**) Average tumor growth curves of subcutaneous Hepa1-6 tumor-bearing mice after treatment as above. Data are presented as means ± s.e.m. (n = 6 mice per group; one-way ANOVA followed by Tukey's HSD post-hoc test). (**l**) Kaplan–Meier survival plot of subcutaneous Hepa1-6 tumor-bearing mice after treatment as above. (n = 6 mice per group). Source data are provided as a Source Data file.

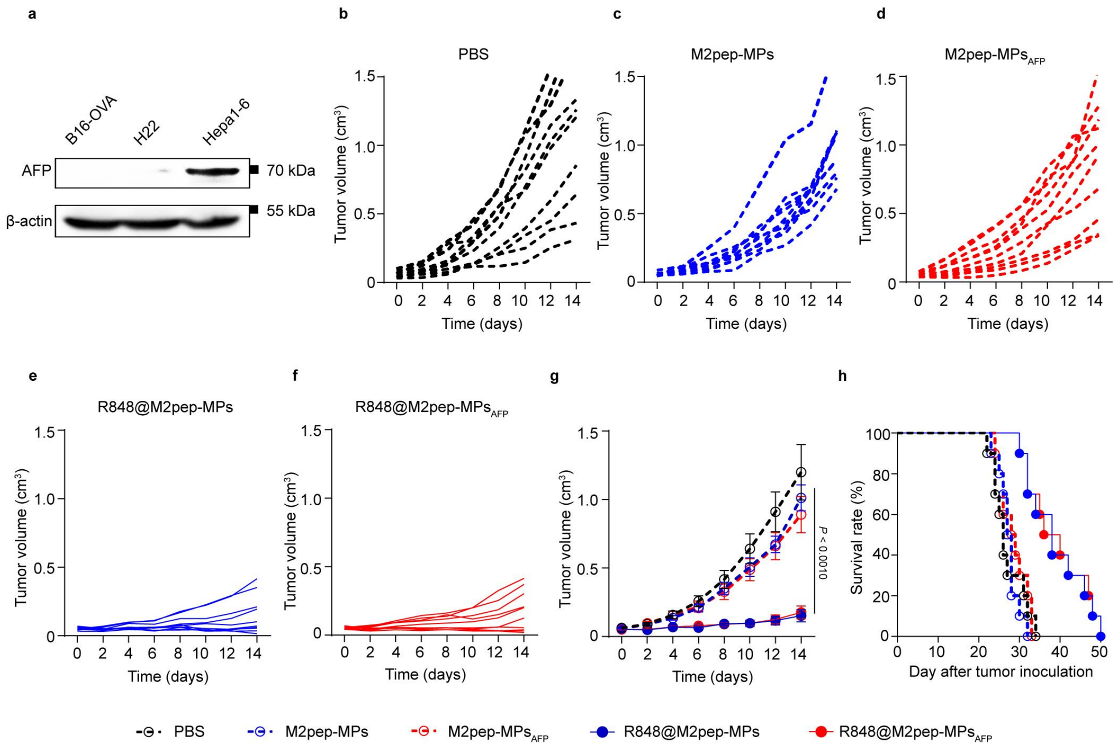

Supplementary Fig. 23 Anticancer activity of R848@M2pep-MPs$_{AFP}$ in subcutaneous H22 tumor-bearing mice. (**a**) AFP expression in B16-OVA, H22 and Hepa1-6 cells by western blotting. Images are representative of three independent samples. (**b-f**) Individual tumor growth curves of H22 tumor-bearing mice after intravenous injection of PBS (**b**), M2pep-MPs (**c**), M2pep-MPs$_{AFP}$ (**d**), R848@M2pep-MPs (**e**) or R848@M2pep-MPs$_{AFP}$ (**f**) every three days for six times at the R848 dosage of 0.5 mg kg^{-1}. (**g**) Average tumor growth curves of H22 tumor-bearing mice after treatment as above. Data are presented as means ± s.e.m. (n = 10 mice per group; one-way ANOVA followed by Tukey's HSD post-hoc test). (**h**) Kaplan–Meier survival plot of H22 tumor-bearing mice after treatment as above. (n = 10 mice per group). Source data are provided as a Source Data file.

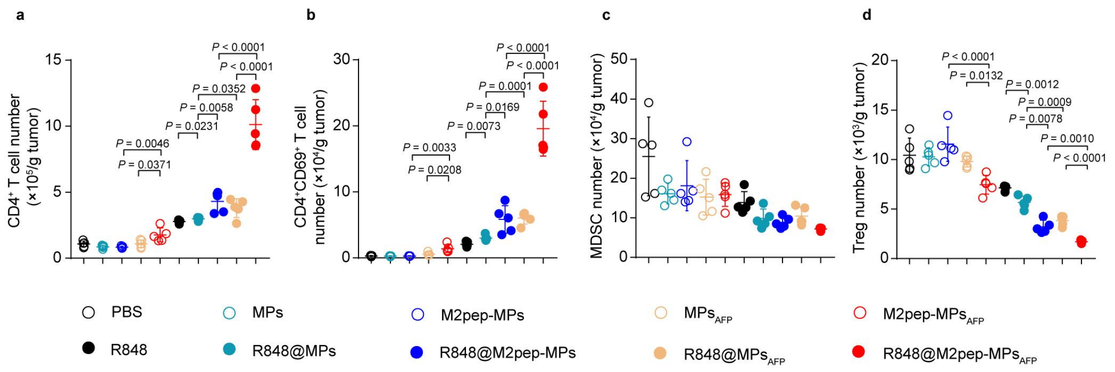

Supplementary Fig. 24 Improved tumor immune microenvironment by R848@M2pep-MPs$_{AFP}$ in orthotopic Hepa1-6 tumor-bearing mice. (**a-d**) The numbers of $CD4^+$ T cells (**a**), $CD4^+CD69^+$ T cells (**b**), MDSCs (**c**) and Tregs (**d**) in tumor tissues of orthotopic Hepa1-6 tumor-bearing mice at 16 days after intravenous injection of PBS, MPs, M2pep-MPs, MPs$_{AFP}$, M2pep-MPs$_{AFP}$, R848, R848@MPs, R848@M2pep-MPs, R848@MPs$_{AFP}$ or R848@M2pep-MPs$_{AFP}$ at the R848 dosage of 0.5 mg kg^{-1} every three days for six times. Data are presented as means ± s.d. (n = 5 mice per group; one-way ANOVA followed by Tukey's HSD post-hoc test). Source data are provided as a Source Data file.

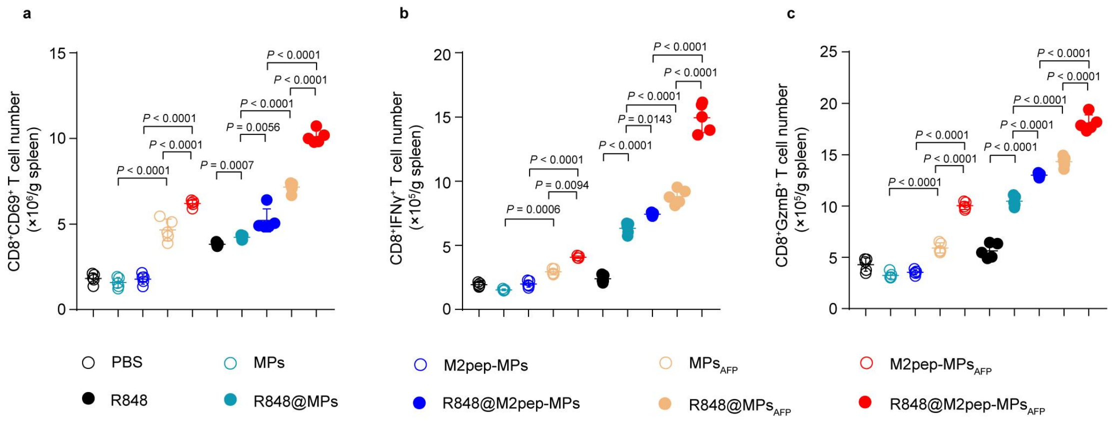

Supplementary Fig. 25 Improved systemic antitumor immunity by R848@M2pep-MPs$_{AFP}$ in orthotopic Hepa1-6 tumor-bearing mice. (**a-c**) The numbers of $CD8^+CD69^+$ T (**a**), $CD8^+IFN\gamma^+$ T (**b**), and $CD8^+GzmB^+$ T (**c**) cells in spleens of orthotopic Hepa1-6 tumor-bearing mice at 16 days after intravenous injection of PBS, MPs, M2pep-MPs, MPs$_{AFP}$, M2pep-MPs$_{AFP}$, R848, R848@MPs, R848@M2pep-MPs, R848@MPs$_{AFP}$ or R848@M2pep-MPs$_{AFP}$ at the R848 dosage of 0.5 mg kg^{-1} every three days for six times. Data are presented as means ± s.d. (n = 5 mice per group; one-way ANOVA followed by Tukey's HSD post-hoc test). Source data are provided as a Source Data file.

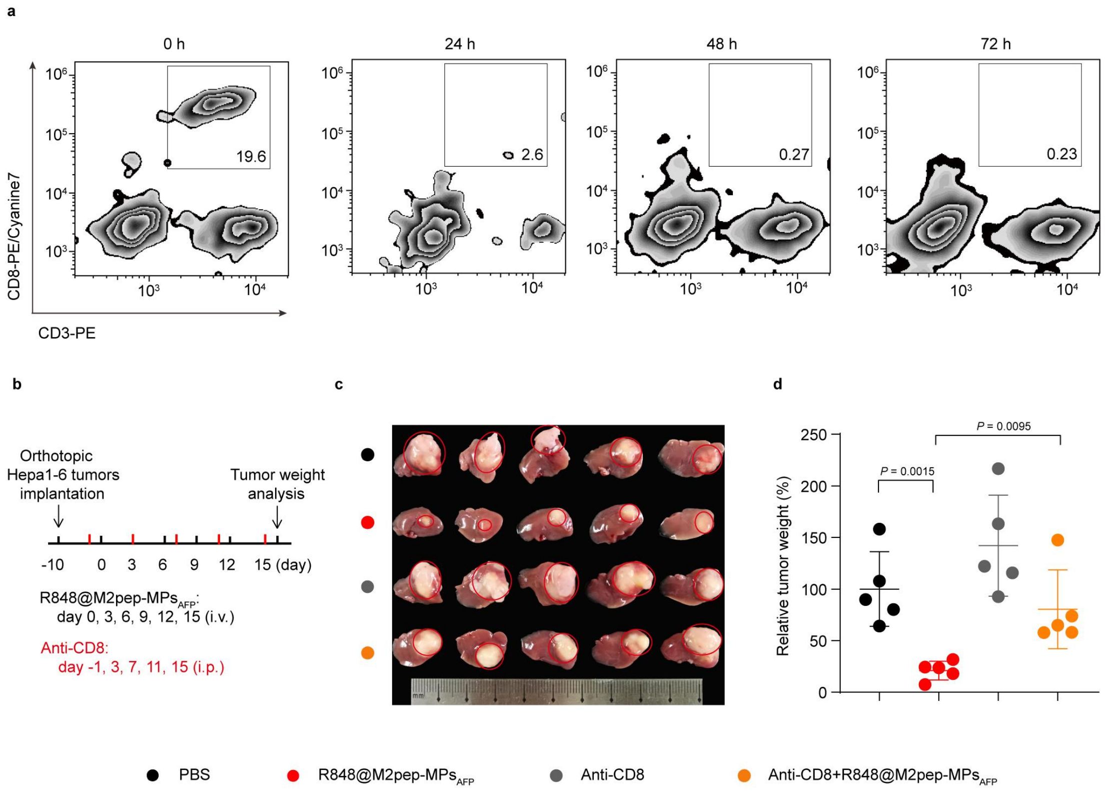

Supplementary Fig. 26 CD8$^+$ T cell-involved in R848@M2pep-MPs$_{AFP}$-induced anticancer effects in orthotopic Hepa1-6 tumor-bearing mice. (**a**) Percentages of CD3$^+$CD8$^+$ T cells in peripheral blood of orthotopic Hepa1-6 tumor-bearing mice at different time intervals after intraperitoneal injection of anti-CD8 antibody at the dosage of 100 μg per mice once. Images are representative of five independent samples. (**b**) Schematic schedule for the anticancer experiments in orthotopic Hepa1-6 tumor-bearing mice after CD8$^+$ T cell depletion. (**c**,**d**) Tumor images (**c**) and tumor weight (**d**) of orthotopic Hepa1-6 tumor-bearing mice after intravenous injection of R848@M2pep-MPs$_{AFP}$ at the R848 dosage of 0.5 mg kg^{-1} every three days for 6 times in the presence or absence of intraperitoneal injection of anti-CD8 antibody at the dosage of 100 μg per mice every four days for 5 times as indicated in (**b**). Data are presented as means ± s.d. (n = 5 mice per group; one-way ANOVA followed by Tukey's HSD post-hoc test). Source data are provided as a Source Data file.

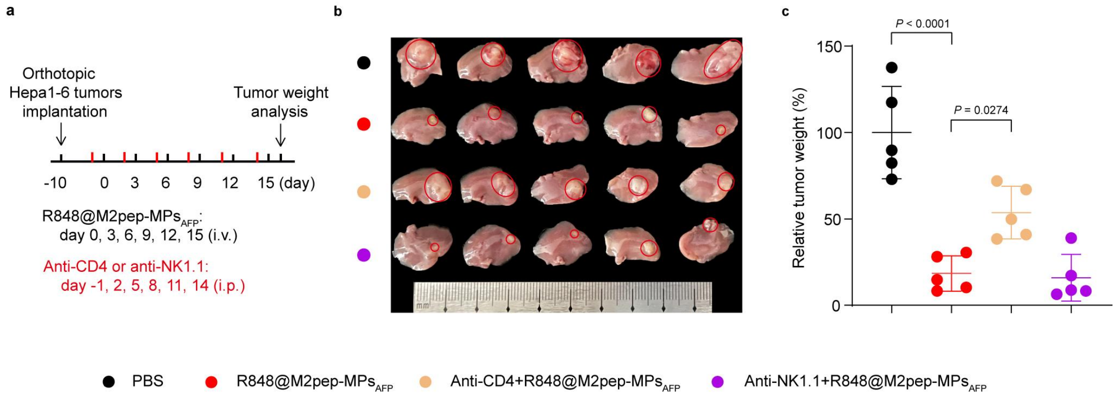

Supplementary Fig. 27 CD4^{+} T cell-involved in R848@M2pep-MPs$_{AFP}$-induced anticancer effects in orthotopic Hepa1-6 tumor-bearing mice. (a) Schematic schedule for the anticancer experiments in orthotopic Hepa1-6 tumor-bearing mice after CD4^{+} T or NK cell depletion. (**b,c**) Tumor images (**b**) and tumor weight (**c**) in orthotopic Hepa1-6 tumor-bearing mice after intravenous injection of R848@M2pep-MPs$_{AFP}$ at the R848 dosage of 0.5 mg kg^{-1} every three days for 6 times in the presence or absence of intraperitoneal injection of anti-CD4 or anti-NK1.1 antibody at the dosage of 100 μg per mice every three days for 6 times as indicated in (**a**). Data are presented as means ± s.d. (n = 5 mice per group; one-way ANOVA followed by Tukey's HSD post-hoc test). Source data are provided as a Source Data file.

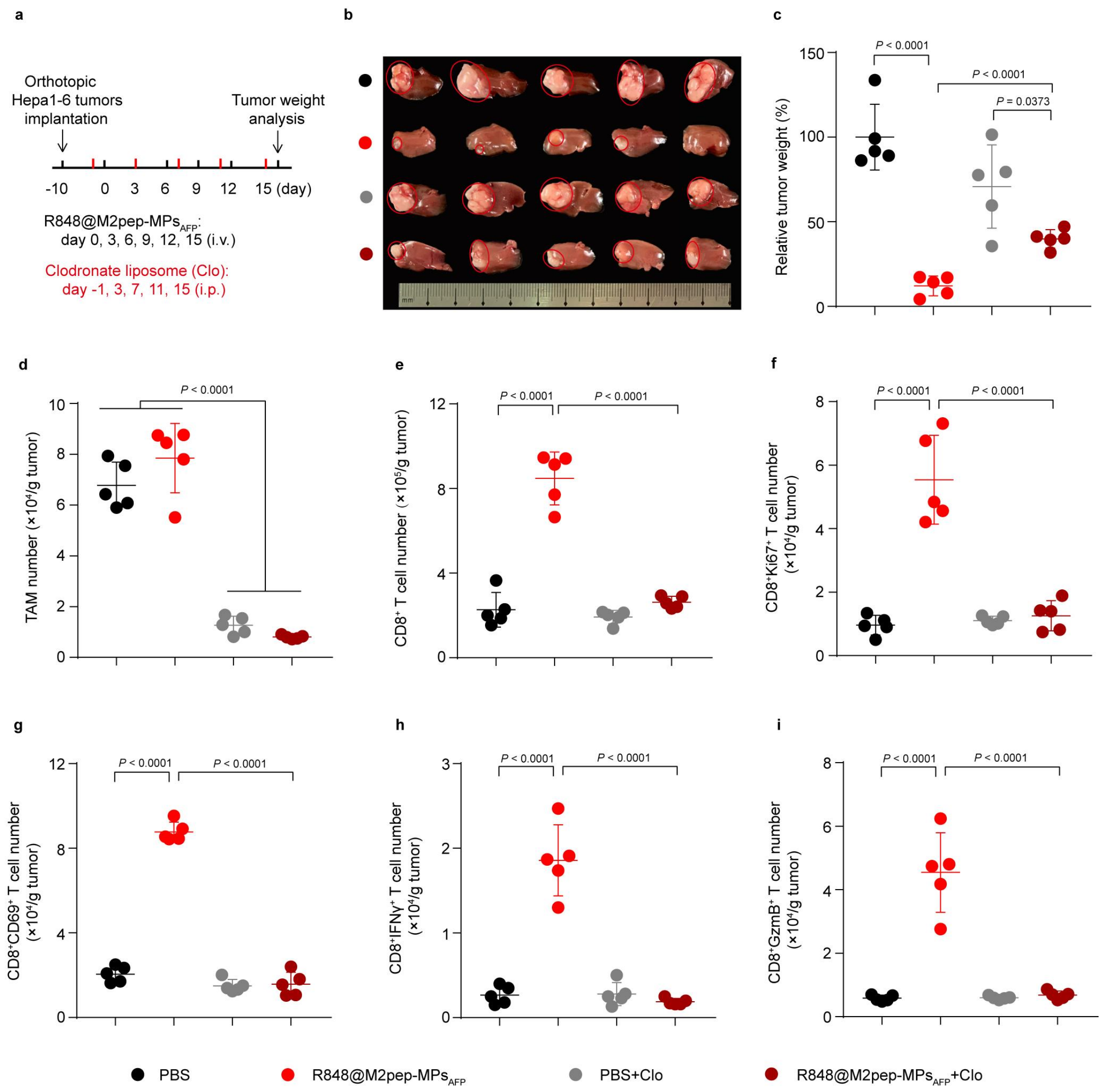

Supplementary Fig. 28 Macrophage-involved in R848@M2pep-MPs$_{AFP}$-induced anticancer effects and antitumor immunity in orthotopic Hepa1-6 tumor-bearing mice. (a) Schematic schedule for the anticancer experiments in orthotopic Hepa1-6 tumor-bearing mice after macrophage depletion. (**b**,**c**) Tumor images (**b**) and tumor weight (**c**) of orthotopic Hepa1-6 tumor-bearing mice after intravenous injection of R848@M2pep-MPs$_{AFP}$ at the R848 dosage of 0.5 mg kg^{-1} every three days for 6 times in the presence or absence of intraperitoneal injection of clodronate liposomes (Clo) at the dosage of 3.75 mg kg^{-1} every four days for 5 times as indicated in (**a**). Data are

presented as means ± s.d. for (**c**) (n = 5 mice per group; one-way ANOVA followed by Tukey's HSD post-hoc test). (**d-i**) The numbers of TAMs (**d**), $CD8^+$ T cells (**e**), $CD8^+Ki67^+$ T cells (**f**), $CD8^+CD69^+$ T cells (**g**), $CD8^+IFN\gamma^+$ T cells (**h**) and $CD8^+GzmB^+$ T cells (**i**) in tumor tissues of orthotopic Hepa1-6 tumor-bearing mice after treatment as indicated in (**a**). Data are presented as means ± s.d. (n = 5 mice per group; one-way ANOVA followed by Tukey's HSD post-hoc test). Source data are provided as a Source Data file.

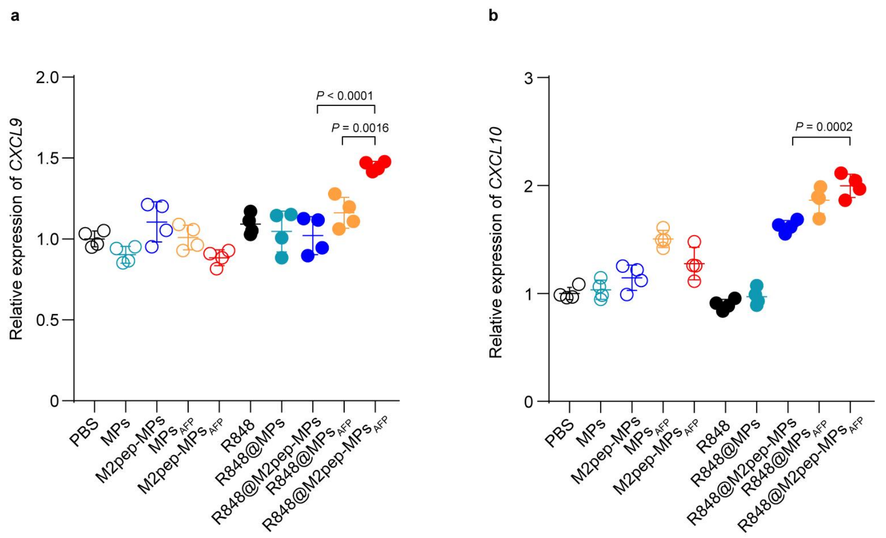

Supplementary Fig. 29 CXCL9 and CXCL10 expression in R848@M2pep-MPs$_{AFP}$-reprogrammed M2-like RAW264.7. (**a**,**b**) The mRNA expression levels of *CXCL9* (**a**) and *CXCL10* (**b**) in IL-4-stimulated RAW264.7 cells after treatment with PBS, MPs, M2pep-MPs, MPs$_{AFP}$, M2pep-MPs$_{AFP}$, R848, R848@MPs, R848@M2pep-MPs, R848@MPs$_{AFP}$ or R848@M2pep-MPs$_{AFP}$ at the R848 concentration of 2 nM for 24 h by RT-qPCR. Data are presented as means ± s.d. (n = 4 biological independent samples; one-way ANOVA followed by Tukey's HSD post-hoc test). Source data are provided as a Source Data file.

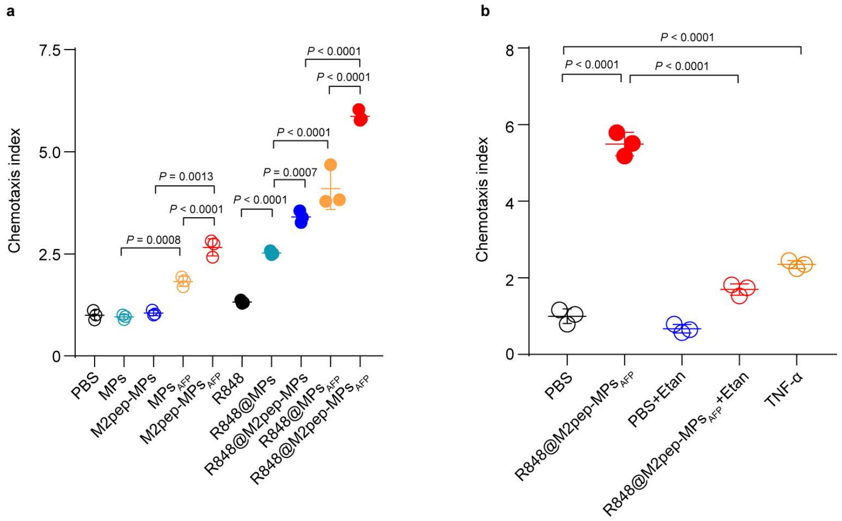

Supplementary Fig. 30 TNF-α-invovled in the recruitment of CD8⁺ T cells by R848@M2pep-MPs$_{AFP}$-reprogrammed macrophages. (a) Chemotaxis index of T lymphocytes seeded in the top chambers at 6 h after co-culture with the supernatants of IL-4-stimulated RAW264.7 cells pretreated with PBS, MPs, M2pep-MPs, MPs$_{AFP}$, M2pep-MPs$_{AFP}$, R848, R848@MPs, R848@M2pep-MPs, R848@MPs$_{AFP}$ or R848@M2pep-MPs$_{AFP}$ at the R848 concentration of 2 nM for 24 h in the bottom chambers. Data are presented as means ± s.d. (n = 3 biological independent samples; one-way ANOVA followed by Tukey's HSD post-hoc test). (**b**) Chemotaxis index of T lymphocytes seeded in the top chambers at 6 h after co-culture with the supernatants of IL-4-stimulated RAW264.7 cells pretreated with PBS or R848@M2pep-MPs$_{AFP}$ at the R848 concentration of 2 nM for 24 h in the presence or absence of Etan at concentration of 0.5 µg mL^{-1} in the bottom chambers by flow cytometry. Data are presented as means ± s.d. (n = 3 biological independent samples; one-way ANOVA followed by Tukey's HSD post-hoc test). Source data are provided as a Source Data file.

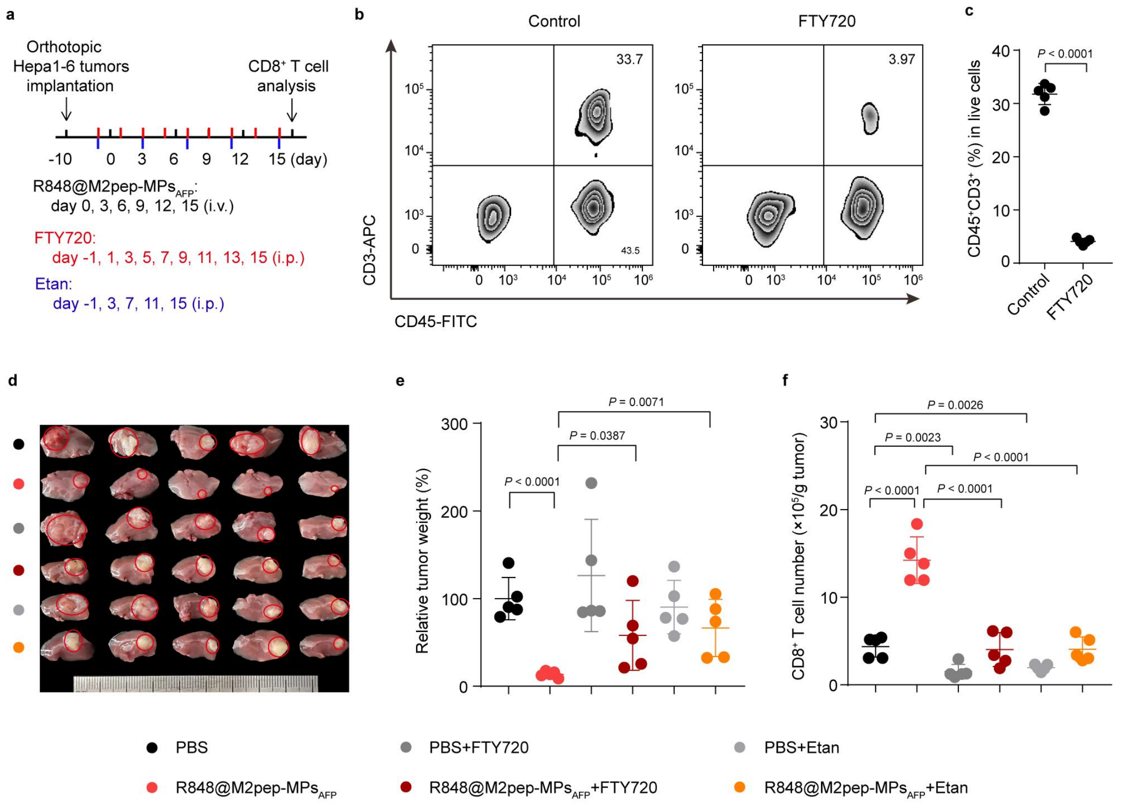

Supplementary Fig. 31 R848@M2pep-MPs$_{AFP}$-induced CD8$^+$ T cell recruitment in orthotopic Hepa1-6 tumor-bearing mice. (**a**) Schematic schedule for R848@M2pep-MPs$_{AFP}$-induced CD8$^+$ T cell recruitment experiment in orthotopic Hepa1-6 tumor-bearing mice. (**b,c**) Representative flow cytometric plots (**b**) and the percentages of CD45$^+$CD3$^+$ T cells in peripheral blood (**c**) of orthotopic Hepa1-6 tumor-bearing mice at 24 h after intraperitoneal injection of FTY720. Data are presented as means ± s.d. (n = 5 mice per group; unpaired two-tailed Student's *t*-test). (**d-f**) Tumor images (**d**), tumor weight (**e**) and CD8$^+$ T cell numbers in tumor tissues (**f**) of orthotopic Hepa1-6 tumor-bearing mice after intravenous injection of PBS or R848@M2pep-MPs$_{AFP}$ at the R848 dosage of 0.5 mg kg^{-1} every three days for 6 times in the presence or absence of intraperitoneal injection of FTY720 (1 mg kg^{-1}) or Etan (5 mg kg^{-1}) as indicated in (**a**). Data are presented as means ± s.d. (n = 5 mice per group; one-way ANOVA followed by Tukey's HSD post-hoc test). Source data are provided as a Source Data file.

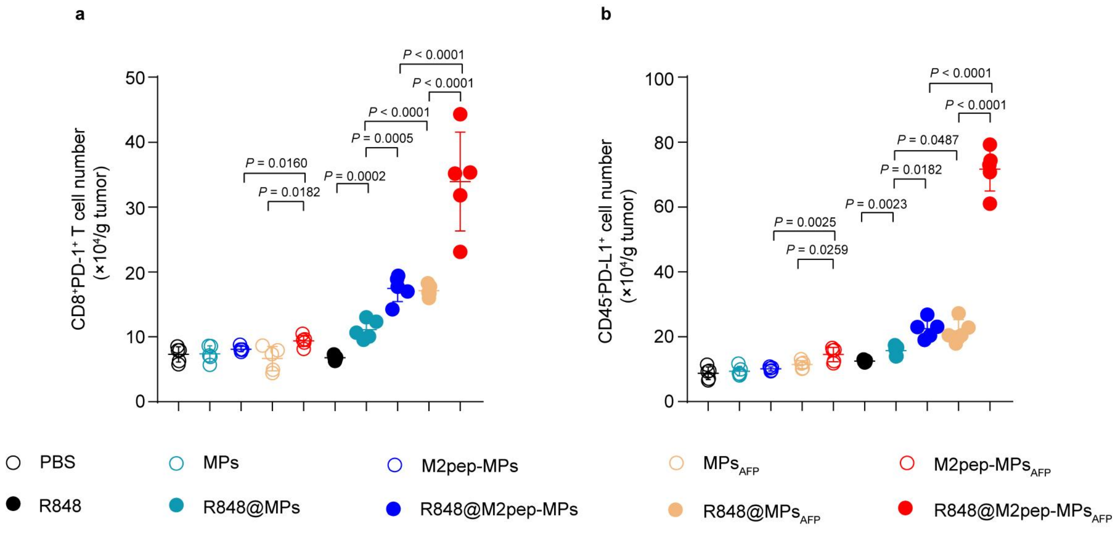

Supplementary Fig. 32 Enhanced expression of PD-1 and PD-L1 by R848@M2pep-MPs$_{AFP}$ in tumors tissues of orthotopic Hepa1-6 tumor-bearing mice. (**a**,**b**) The numbers of CD8^{+}PD-1^{+} T cells (**a**) and CD45^{-}PD-L1^{+} tumor cells (**b**) in tumor tissues of orthotopic Hepa1-6 tumor-bearing mice after intravenous injection of PBS, MPs, M2pep-MPs, MPs$_{AFP}$, M2pep-MPs$_{AFP}$, R848, R848@MPs, R848@M2pep-MPs, R848@MPs$_{AFP}$ or R848@M2pep-MPs$_{AFP}$ at the R848 dosage of 0.5 mg kg^{-1} every three days for six times. Data are presented as means ± s.d. (n = 5 mice per group; one-way ANOVA followed by Tukey's HSD post-hoc test). Source data are provided as a Source Data file.

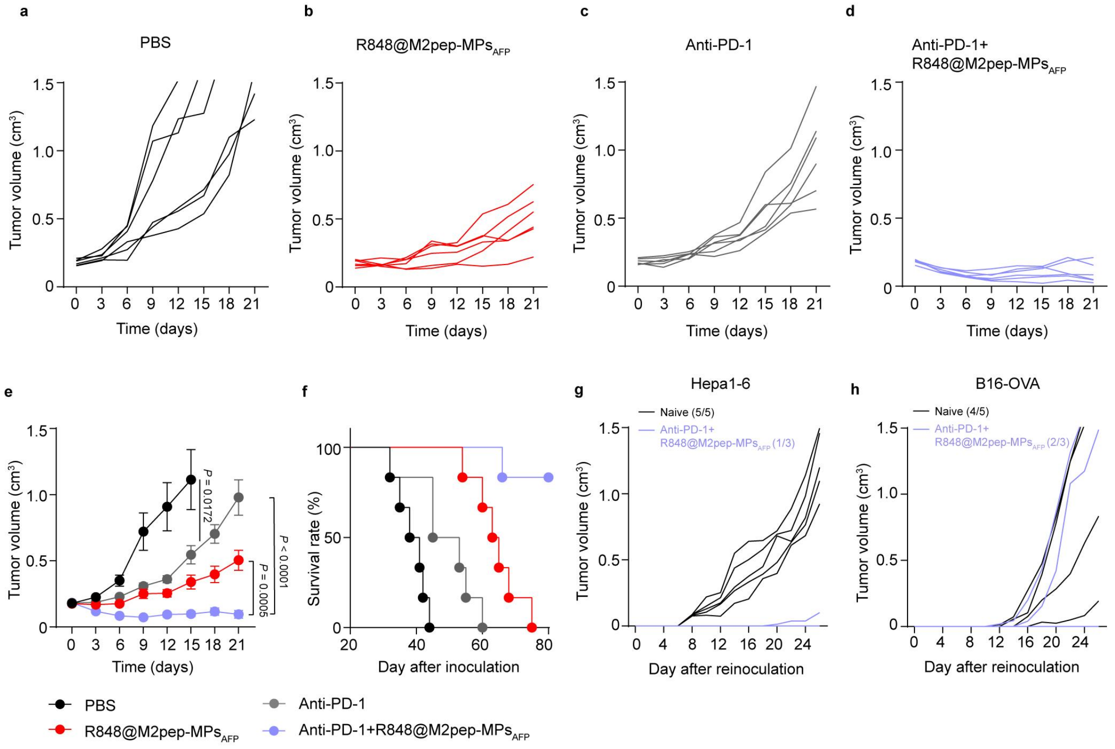

Supplementary Fig. 33 Potent anticancer activity of combination of anti-PD-1 antibody and R848@M2pep-MPs$_{AFP}$ in subcutaneous Hepa1-6 tumor-bearing mice. (**a-d**) Individual tumor growth curves of subcutaneous Hepa1-6 tumor-bearing mice after treatment with PBS (**a**), R848@M2pep-MPs$_{AFP}$ (**b**), anti-PD-1 antibody (**c**) or the combination of R848@M2pep-MPs$_{AFP}$ and anti-PD-1 antibody (**d**) at the anti-PD-1 antibody dosage of 5 mg kg^{-1} and R848 dosage of 0.5 mg kg^{-1} indicated in Fig. **6a**. (**e**) Average tumor growth curves of subcutaneous Hepa1-6 tumor-bearing mice after treatment as above. Data are presented as means ± s.e.m. (n = 6 mice per group; one-way ANOVA followed by Tukey's HSD post-hoc test). (**f**) Kaplan–Meier survival plot of subcutaneous Hepa1-6 tumor-bearing mice after treatment as above. (n = 6 mice per group). (**g,h**) Individual tumor growth curves after rechallenge with Hepa1-6 cells (3 × 10^{6} cells, **g**) and B16-OVA cells (1 × 10^{6} cells, **h**) in naïve mice (n = 5 mice) or combination of anti-PD-1 antibody and R848@M2pep-MPs$_{AFP}$-cured mice (n = 3 mice). Source data are provided as a Source Data file.

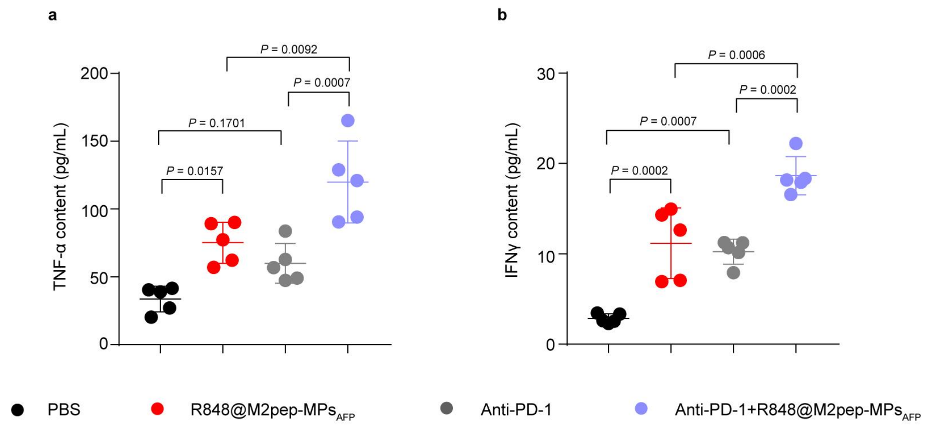

Supplementary Fig. 34 Cytokine contents in serum of orthotopic Hepa1-6 tumor-bearing mice after treatment with R848@M2pep-MPs$_{AFP}$ and anti-PD-1 antibody. (**a**,**b**) The contents of TNF-α (**a**) and IFNγ (**b**) in serum of orthotopic Hepa1-6 tumor-bearing mice after treatment with PBS, R848@M2pep-MPs$_{AFP}$, anti-PD-1 antibody or a combination of R848@M2pep-MPs$_{AFP}$ and anti-PD-1 antibody at the anti-PD-1 antibody dosage of 5 mg kg^{-1} and R848 dosage of 0.5 mg kg^{-1} indicated in Fig. **6a**. Data are presented as means ± s.d. (n = 5 mice per group; one-way ANOVA followed by Tukey's HSD post-hoc test). Source data are provided as a Source Data file.

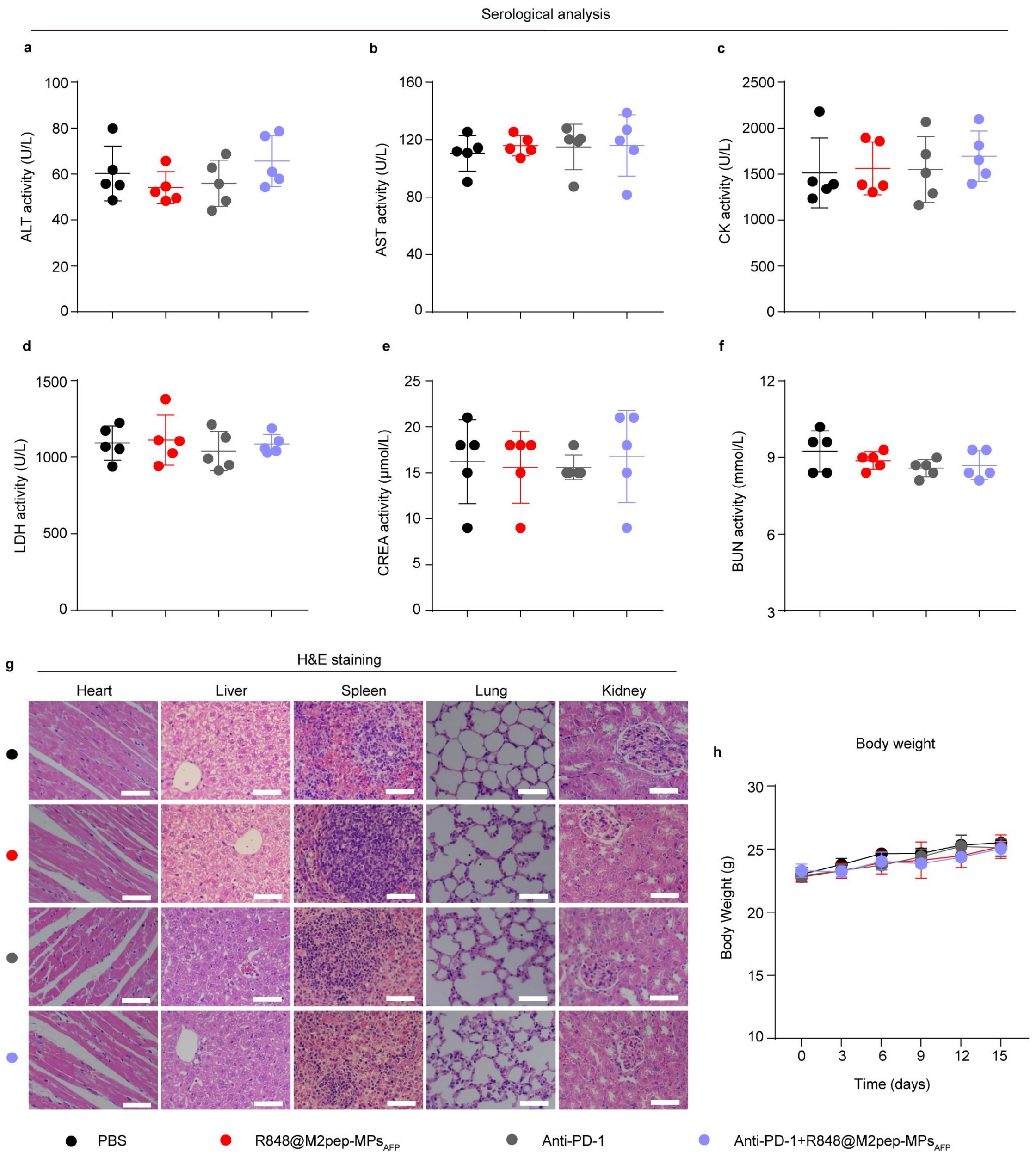

Supplementary Fig. 35 Biosafety of combination of R848@M2pep-MPs$_{AFP}$ and anti-PD-1 antibody in orthotopic Hepa1-6 tumor-bearing mice. (a-f) The activity of ALT (**a**), AST (**b**), CK (**c**), LDH (**d**), CREA (**e**) and BUN (**f**) in serum of orthotopic Hepa1-6 tumor-bearing mice after treatment with PBS, R848@M2pep-MPs$_{AFP}$, anti-PD-1 antibody or a combination of R848@M2pep-MPs$_{AFP}$ and anti-PD-1 antibody at the anti-PD-1 antibody dosage of 5 mg kg^{-1} and R848 dosage of 0.5 mg kg^{-1} indicated in Fig. **6a**. Data are presented as means ± s.d. (n = 5 mice per group;

one-way ANOVA followed by Tukey's HSD post-hoc test). (**g**) H&E staining of major organs in orthotopic Hepa1-6 tumor-bearing mice after treatment indicated in Fig. **6a**. Images are representative of 3 biologically independent mice. Scale bars: 20 μm. (**h**) Body weight of orthotopic Hepa1-6 tumor-bearing mice during the treatment as indicated in Fig. **6a**. Data are presented as means ± s.d. (n = 5 mice per group). Source data are provided as a Source Data file.

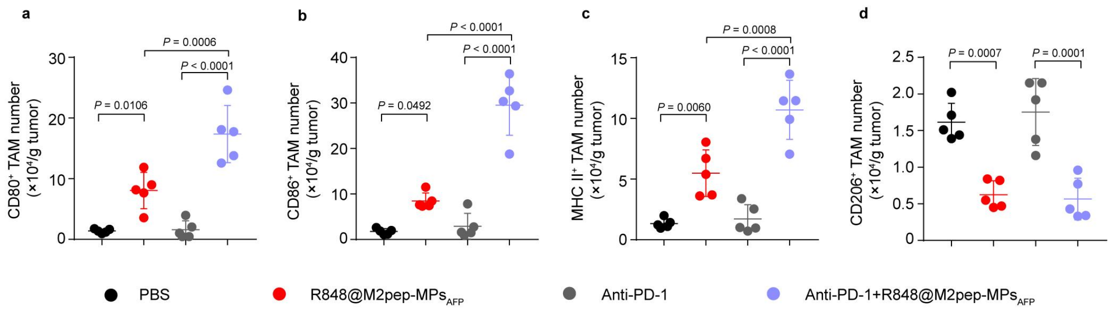

Supplementary Fig. 36 Improved tumor immune microenvironment by combination of anti-PD-1 antibody and R848@M2pep-MPs$_{AFP}$ in orthotopic Hepa1-6 tumor-bearing mice. (**a-d**) The numbers of CD80^{+} TAMs (**a**), CD86^{+} TAMs (**b**), MHC II^{+} TAMs (**c**) and CD206^{+} TAMs (**d**) in tumor tissues of orthotopic Hepa1-6 tumor-bearing mice after treatment with PBS, R848@M2pep-MPs$_{AFP}$, anti-PD-1 antibody or a combination of R848@M2pep-MPs$_{AFP}$ and anti-PD-1 antibody at the anti-PD-1 antibody dosage of 5 mg kg^{-1} and R848 dosage of 0.5 mg kg^{-1} indicated in Fig. **6a**. Data are presented as means ± s.d. (n = 5 mice per group; one-way ANOVA followed by Tukey's HSD post-hoc test). Source data are provided as a Source Data file.

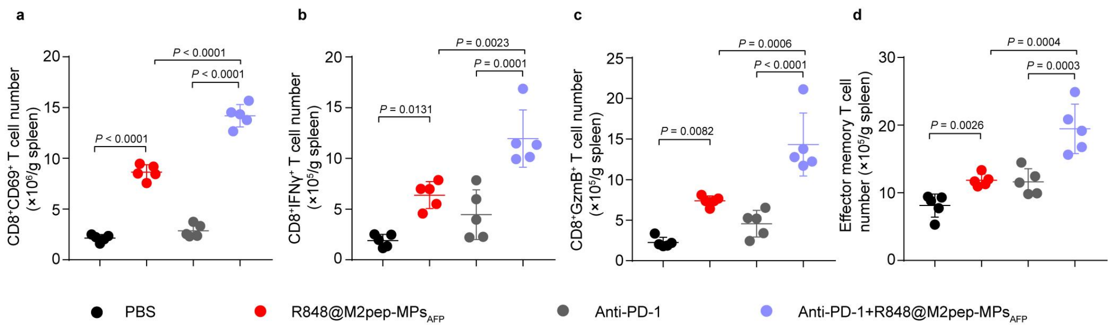

Supplementary Fig. 37 Improved systemic antitumor immunity by combination of anti-PD-1 antibody and R848@M2pep-MPs$_{AFP}$ in orthotopic Hepa1-6 tumor-bearing mice. (**a-d**) The numbers of $CD8^+CD69^+$ T cells (**a**), $CD8^+IFN\gamma^+$ T cells (**b**), $CD8^+GzmB^+$ T cells (**c**) and effector memory T cells (gated as $CD45^+CD3^+CD8^+CD44^+CD62L^-$, **d**) in spleens of orthotopic Hepa1-6 tumor-bearing mice after treatment with PBS, R848@M2pep-MPs$_{AFP}$, anti-PD-1 antibody or a combination of R848@M2pep-MPs$_{AFP}$ and anti-PD-1 antibody at the anti-PD-1 antibody dosage of 5 mg kg^{-1} and R848 dosage of 0.5 mg kg^{-1} indicated in Fig. **6a**. Data are presented as means ± s.d. (n = 5 mice per group; one-way ANOVA followed by Tukey's HSD post-hoc test). Source data are provided as a Source Data file.

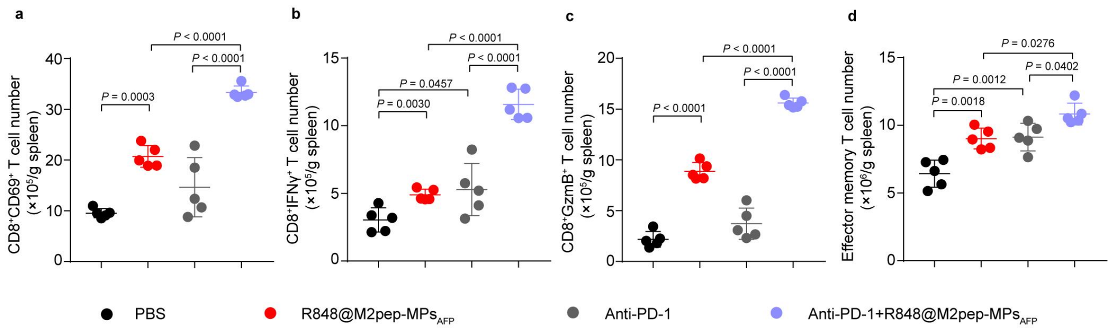

Supplementary Fig. 38 Improved systemic antitumor immunity by combination of anti-PD-1 antibody and R848@M2pep-MPs$_{AFP}$ in DEN-induced autochthonous HCC mice. (**a-d**) The numbers of CD8$^+$CD69$^+$ T cells (**a**), CD8$^+$IFNγ$^+$ T cells (**b**), CD8$^+$GzmB$^+$ T cells (**c**) and effector memory T cells (**d**) in spleens of DEN-induced autochthonous HCC mice after treatment with PBS, R848@M2pep-MPs$_{AFP}$, anti-PD-1 antibody or a combination of R848@M2pep-MPs$_{AFP}$ and anti-PD-1 antibody at the anti-PD-1 antibody dosage of 5 mg kg^{-1} and R848 dosage of 0.5 mg kg^{-1} indicated in Fig. **7a**. Data are presented as means ± s.d. (n = 5 mice per group; one-way ANOVA followed by Tukey's HSD post-hoc test). Source data are provided as a Source Data file.

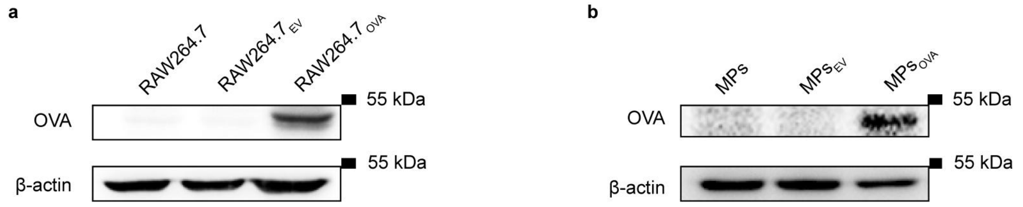

Supplementary Fig. 39 OVA expression in RAW264.7$_{OVA}$ cells and MPs$_{OVA}$. (**a**) OVA expression in RAW264.7, RAW264.7$_{EV}$ and RAW264.7$_{OVA}$ cells by western blotting. (**b**) OVA expression in MPs, MPs$_{EV}$ and MPs$_{OVA}$ by western blotting. Images are representative of three independent samples.

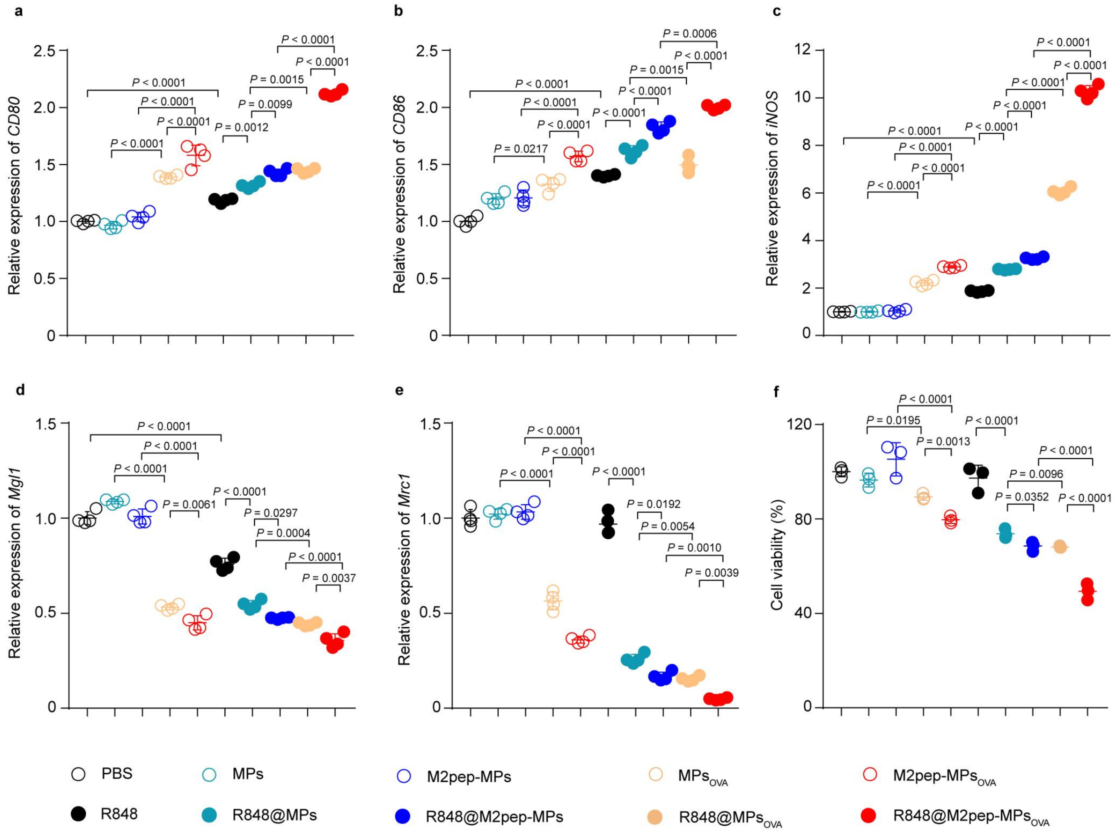

Supplementary Fig. 40 Reprogramming of M2-like macrophages to M1-like phenotype by R848@M2pep-MPs$_{OVA}$. (**a-e**) The mRNA expression levels of *CD80* (**a**), *CD86* (**b**), *iNOS* (**c**), *Mgl1* (**d**) and *Mrc1* (**e**) in IL-4-stimulated RAW264.7 cells after treatment with PBS, MPs, M2pep-MPs, MPs$_{OVA}$, M2pep-MPs$_{OVA}$, R848, R848@MPs, R848@M2pep-MPs, R848@MPs$_{OVA}$ or R848@M2pep-MPs$_{OVA}$ at the R848 concentration of 2 nM for 24 h by RT-qPCR. Data are presented as means ± s.d. (n = 4 biological independent samples; one-way ANOVA followed by Tukey's HSD post-hoc test). (**f**) Cell viability of B16-OVA cells at 24 h after treatment with the supernatants of the above treated IL-4-stimulated RAW264.7 cells by CCK-8 assay. Data are presented as means ± s.d. (n = 3 biological independent samples; one-way ANOVA followed by Tukey's HSD post-hoc test). Source data are provided as a Source Data file.

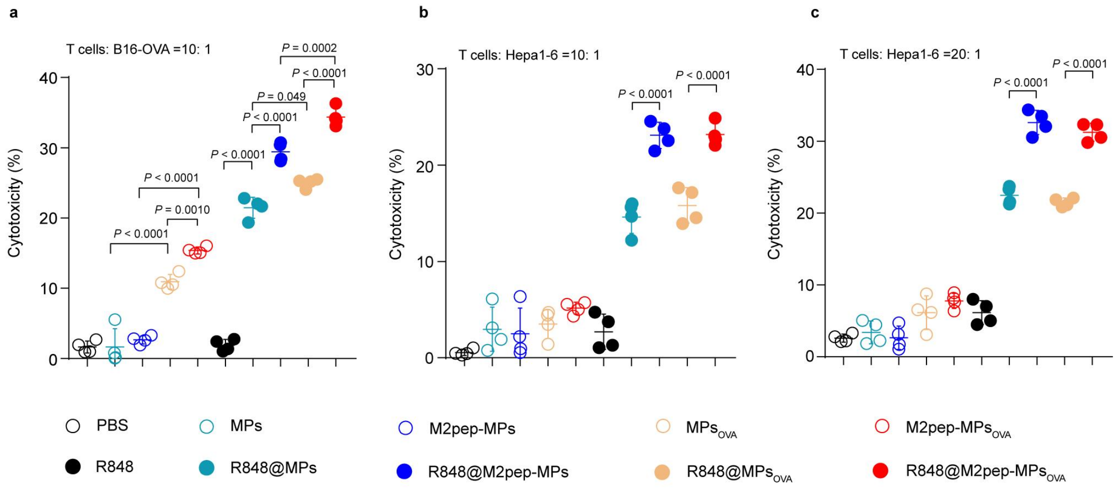

Supplementary Fig. 41 Cytotoxicity of activated CD8$^+$ T cells by R848@M2pep-MPs$_{OVA}$-reprogrammed M2-like macrophages against B16-OVA or Hepa1-6 cells. (**a-c**) Cytotoxicity of CD8$^+$ T cells against B16-OVA or Hepa1-6 cells when the CD8$^+$ T cells cocultured with PBS-, MPs-, M2pep-MPs-, MPs$_{OVA}$-, M2pep-MPs$_{OVA}$-, R848-, R848@MPs-, R848@M2pep-MPs-, R848@MPs$_{OVA}$- or R848@M2pep-MPs$_{OVA}$-pretreated IL-4-stimulated RAW264.7 cells (R848 concentration at 2 nM for 24 h) for 5 days were incubated with B16-OVA or Hepa1-6 cells at the effector/target ratio of 10:1 (**a,b**) and 20:1 (**c**) for 6 h by LDH assay. Data are presented as means ± s.d. (n = 4 biological independent samples; one-way ANOVA followed by Tukey's HSD post-hoc test). Source data are provided as a Source Data file.

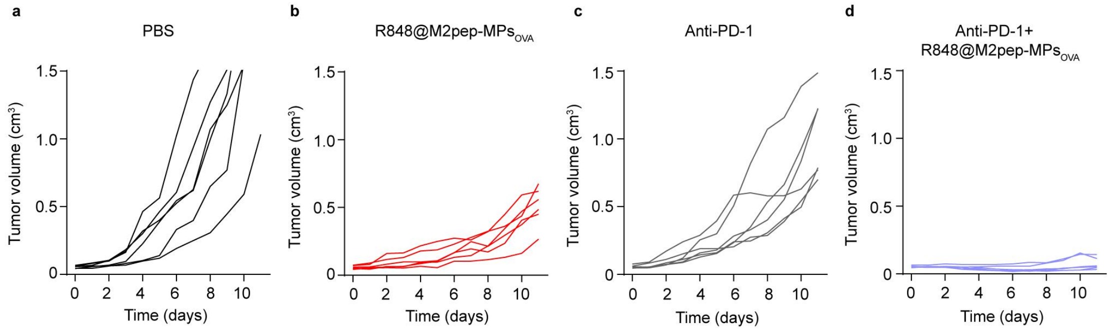

Supplementary Fig. 42 Antitumor effects of combination of R848@M2pep-MPs$_{OVA}$ and anti-PD-1 antibody in B16-OVA tumor-bearing mice. (**a-d**) Individual tumor growth curves of B16-OVA tumor-bearing mice after treatment with PBS (**a**), R848@M2pep-MPs$_{OVA}$ (**b**), anti-PD-1 antibody (**c**) or a combination of R848@M2pep-MPs$_{OVA}$ and anti-PD-1 antibody (**d**) at the anti-PD-1 antibody dosage of 5 mg kg^{-1} and R848 dosage of 0.5 mg kg^{-1} indicated in Fig. **8i**. (n = 6 mice per group). Source data are provided as a Source Data file.

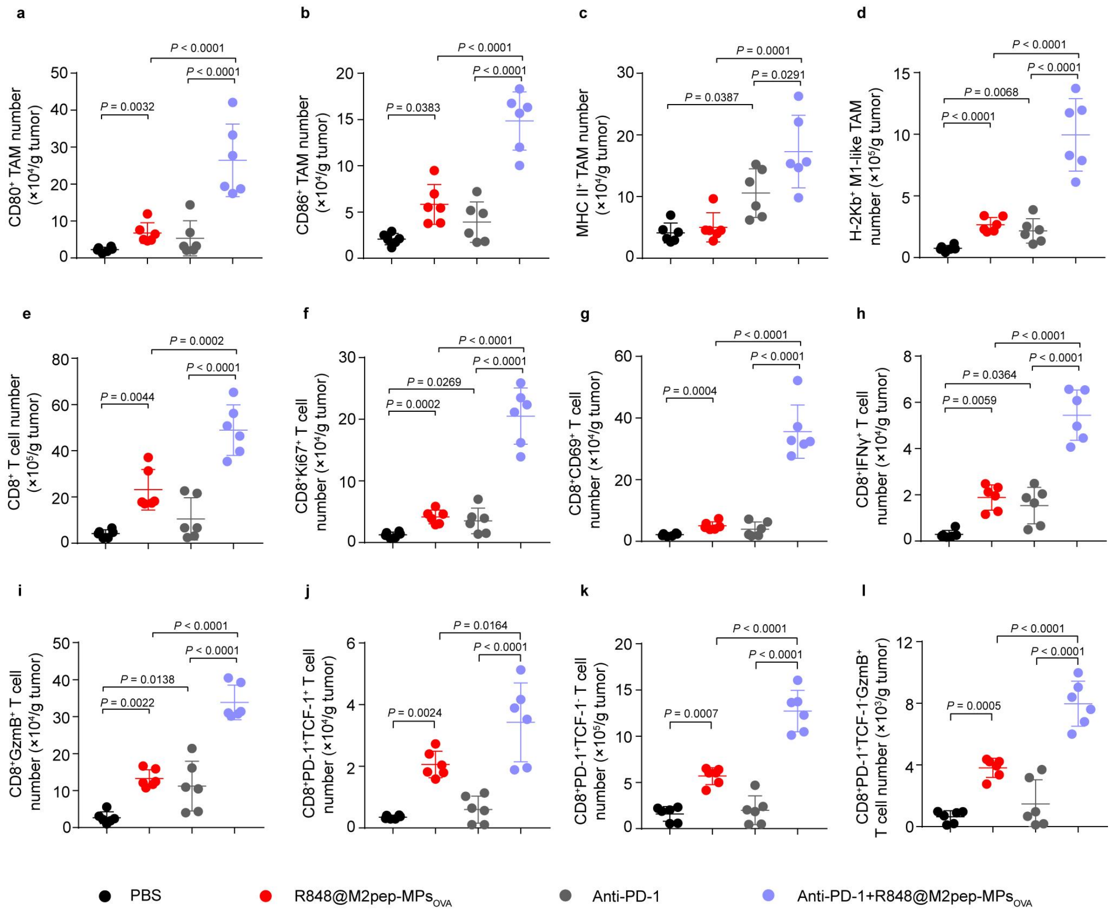

Supplementary Fig. 43 Improved tumor immune microenvironment induced by combination of R848@M2pep-MPs$_{OVA}$ and anti-PD-1 antibody in B16-OVA tumor-bearing mice. (**a-l**) The numbers of CD80$^+$ TAMs (**a**), CD86$^+$ TAMs (**b**), MHC II$^+$ TAMs (**c**), H-2Kb$^+$ M1-like TAMs (**d**), CD8$^+$ T cells (**e**), CD8$^+$Ki67$^+$ T cells (**f**), CD8$^+$CD69$^+$ T cells (**g**), CD8$^+$IFNγ$^+$ T cells (**h**), CD8$^+$GzmB$^+$ T cells (**i**), CD8$^+$PD-1$^+$TCF-1$^+$ T cells (**j**), CD8$^+$PD-1$^+$TCF-1$^-$ T cells (**k**) and CD8$^+$PD-1$^+$TCF-1$^-$GzmB$^+$ T cells (**l**) in tumor tissues of B16-OVA tumor-bearing mice at 11 days after treatment with PBS, R848@M2pep-MPs$_{OVA}$, anti-PD-1 antibody or a combination of R848@M2pep-MPs$_{OVA}$ and anti-PD-1 antibody at the anti-PD-1 antibody dosage of 5 mg kg^{-1} and R848 dosage of 0.5 mg kg^{-1} indicated in Fig. **8i**. Data are presented as means ± s.d. (n = 6 mice per group; one-way ANOVA followed by Tukey's HSD post-hoc test). Source data are provided as a Source Data file.

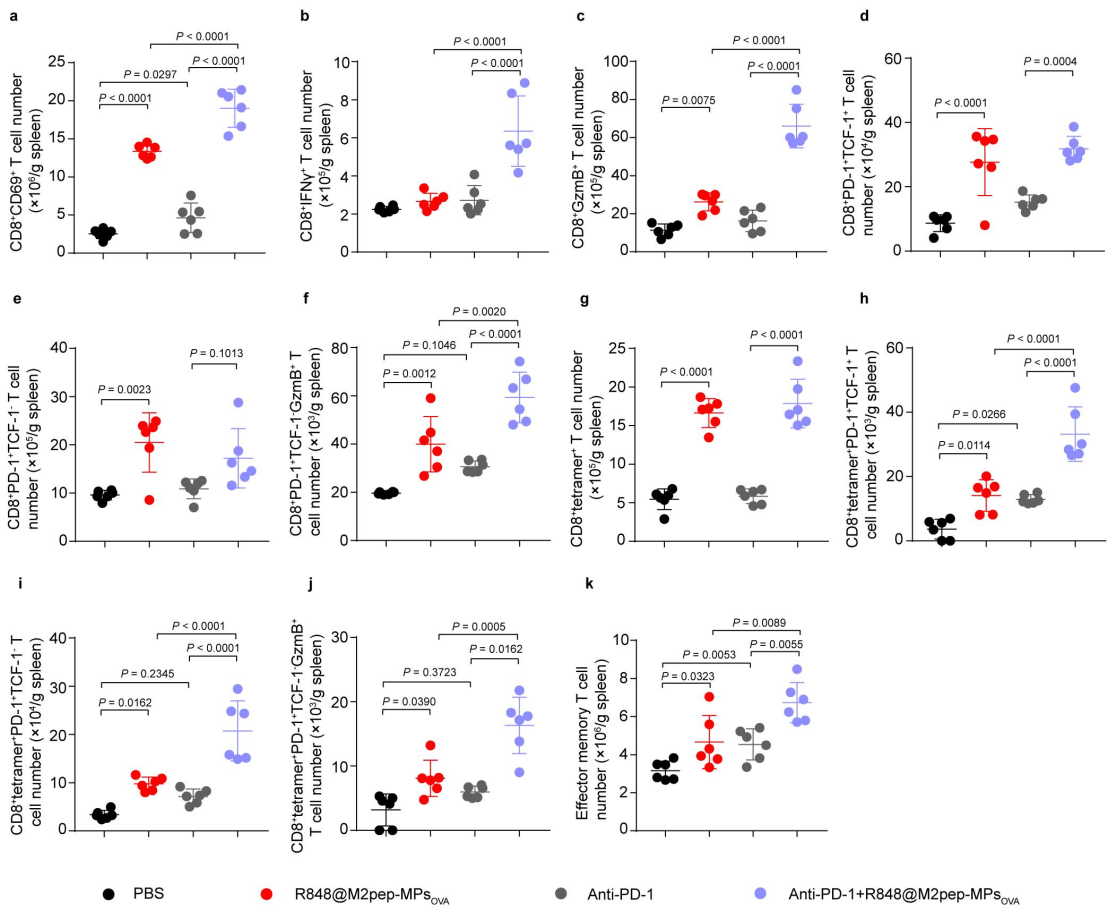

Supplementary Fig. 44 Improved immune microenvironment in spleens of B16-OVA tumor-bearing mice after treatment with R848@M2pep-MPs$_{OVA}$ and anti-PD-1 antibody. (**a-k**) The numbers of $CD8^+CD69^+$ T cells (**a**), $CD8^+IFN\gamma^+$ T cells (**b**), $CD8^+GzmB^+$ T cells (**c**), $CD8^+PD\text{-}1^+TCF\text{-}1^+$ T cells (**d**), $CD8^+PD\text{-}1^+TCF\text{-}1^-$ T cells (**e**), $CD8^+PD\text{-}1^+TCF\text{-}1^-GzmB^+$ T cells (**f**), $CD8^+tetramer^+$ T cells (**g**), $CD8^+tetramer^+PD\text{-}1^+TCF\text{-}1^+$ T cells (**h**), $CD8^+tetramer^+PD\text{-}1^+TCF\text{-}1^-$ T cells (**i**), $CD8^+tetramer^+PD\text{-}1^+TCF\text{-}1^-GzmB^+$ T cells (**j**) and effector memory T cells (**k**) in spleens of B16-OVA tumor-bearing mice at 11 days after treatment with PBS, R848@M2pep-MPs$_{OVA}$, anti-PD-1 antibody, or a combination of R848@M2pep-MPs$_{OVA}$ and anti-PD-1 antibody at the anti-PD-1 antibody dosage of 5 mg kg^{-1} and R848 dosage of 0.5 mg kg^{-1} indicated in Fig. **8i**. Data are presented as means ± s.d. (n = 6 mice per group; one-way ANOVA followed by Tukey's HSD post-hoc test). Source data are provided as a Source Data file.

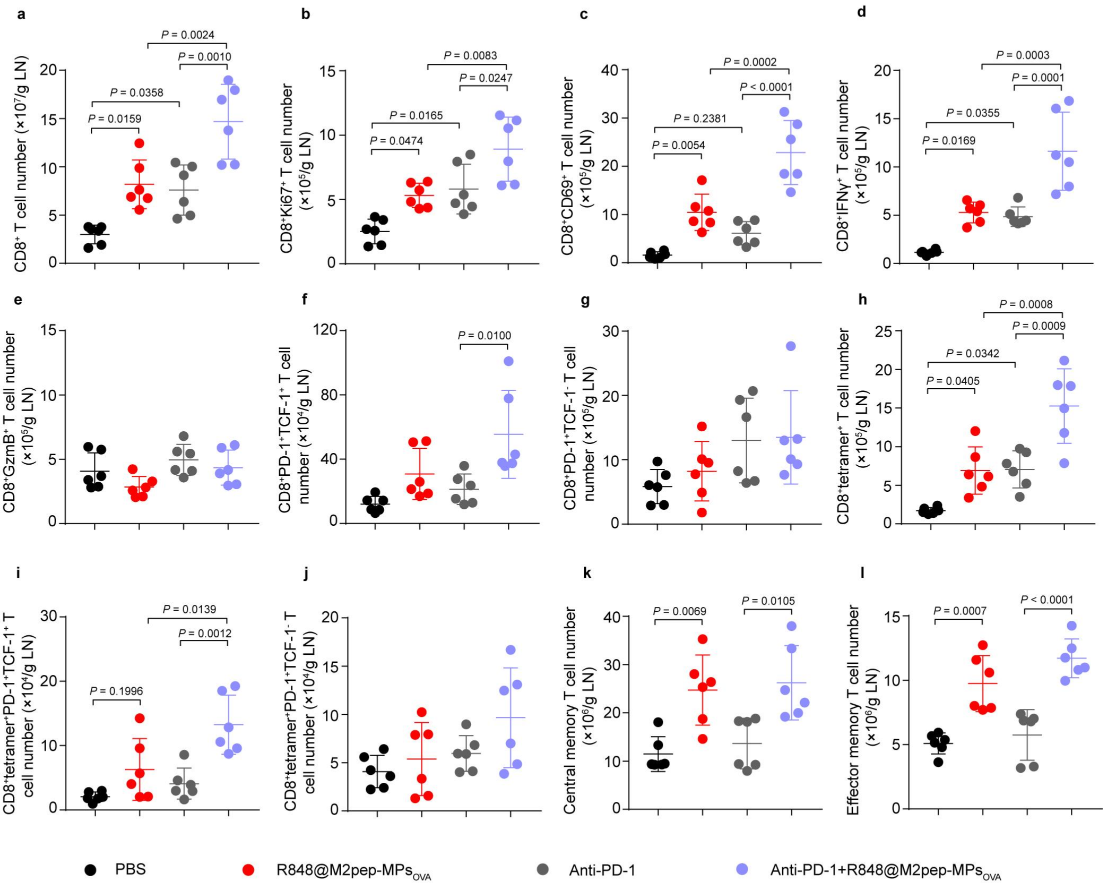

Supplementary Fig. 45 Improved tumor immune microenvironment in tumor-draining lymph nodes by combination of R848@M2pep-MPs$_{OVA}$ and anti-PD-1 antibody in B16-OVA tumor-bearing mice. (**a-l**) The numbers of CD8$^+$ T cells (**a**), CD8$^+$Ki67$^+$ T cells (**b**), CD8$^+$CD69$^+$ T cells (**c**), CD8$^+$IFNγ$^+$ T cells (**d**), CD8$^+$GzmB$^+$ T cells (**e**), CD8$^+$PD-1$^+$TCF-1$^+$ T cells (**f**), CD8$^+$PD-1$^+$TCF-1$^-$ T cells (**g**), CD8$^+$tetramer$^+$ T cells (**h**), CD8$^+$tetramer$^+$PD-1$^+$TCF-1$^+$ T cells (**i**), CD8$^+$tetramer$^+$PD-1$^+$TCF-1$^-$ T cells (**j**), Tcm cells (**k**) and Tem cells (**l**) in tumor-draining lymph nodes of B16-OVA tumor-bearing mice at 11 days after treatment with PBS, R848@M2pep-MPs$_{OVA}$, anti-PD-1 antibody, or a combination of R848@M2pep-MPs$_{OVA}$ and anti-PD-1 antibody at the anti-PD-1 antibody dosage of 5 mg kg^{-1} and R848 dosage of 0.5 mg kg^{-1} indicated in Fig. **8i**. Data are presented as means ± s.d. (n = 6 mice per group; one-way ANOVA followed by Tukey's HSD post-hoc test). Source data are provided as a Source Data file.

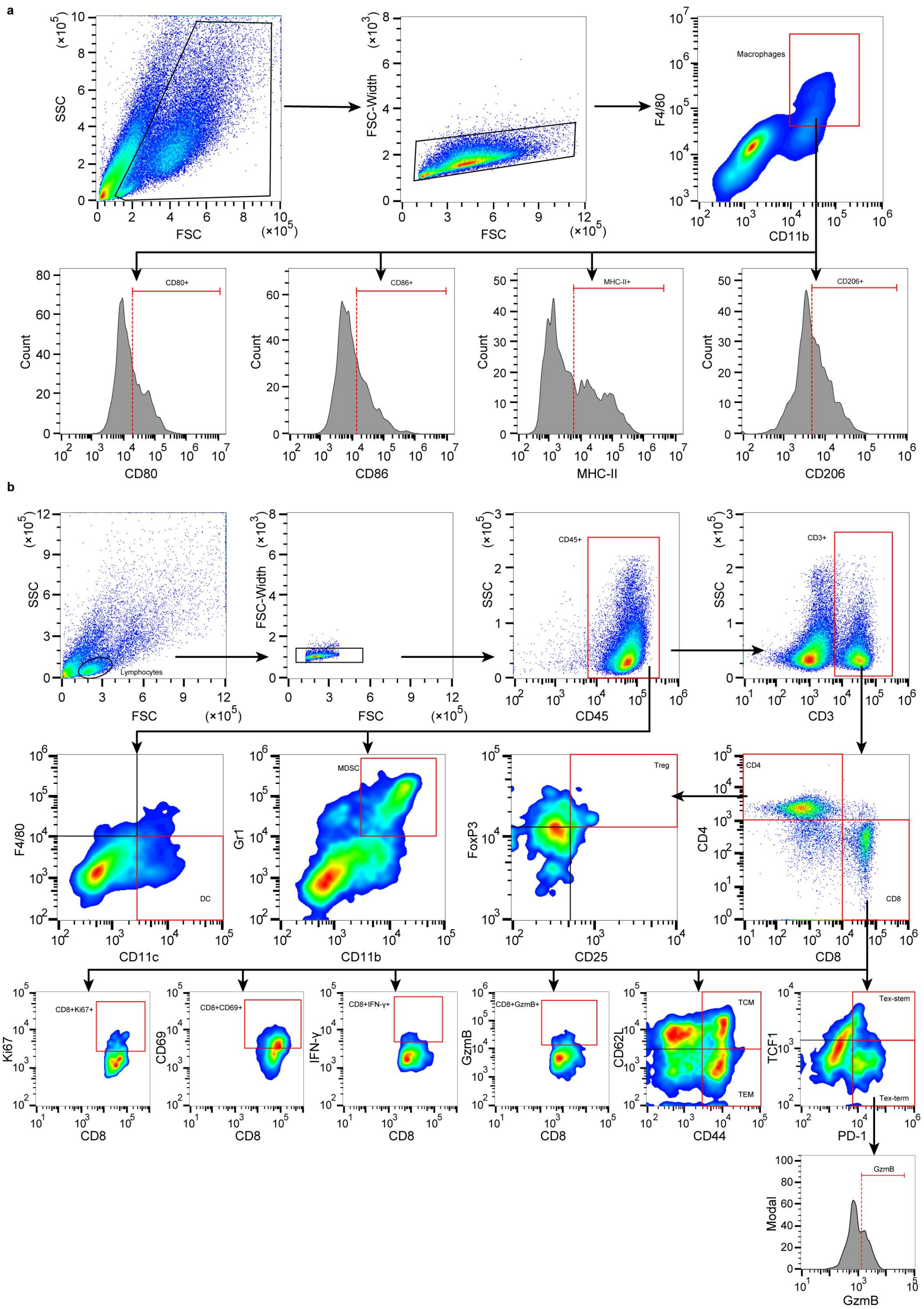

Supplementary Fig. 46 Gating strategy for identifying major immune cell populations in tumor tissues of tumor-bearing mice. (**a**) Gating strategy for identifying M1- and M2-like TAMs in tumor tissues of tumor-bearing mice presented

on Fig. 3g, h, 4b-e, 7g-j, 8l and Supplementary Fig. 28d, 36a-d and 43a-d. (**b**) Gating strategy for identifying DCs (CD45^{+}F4/80^{-}CD11c^{+} cells), MDSCs (CD45^{+}CD11b^{+}Gr1^{+} cells), Tregs (CD45^{+}CD3^{+}CD4^{+}CD25^{+}FoxP3^{+} cells), CD4^{+} T cells (CD45^{+}CD3^{+}CD4^{+} cells), CD8^{+} T cells (CD45^{+}CD3^{+}CD4^{+} cells), CD8^{+}CD69^{+} T cells (CD45^{+}CD3^{+}CD8^{+}CD69^{+} cells), CD8^{+}Ki67^{+} T cells (CD45^{+}CD3^{+}CD8^{+}Ki67^{+} cells), CD8^{+}IFNγ^{+} T cells (CD45^{+}CD3^{+}CD8^{+}IFNγ^{+} cells), CD8^{+}GzmB^{+} T cells (CD45^{+}CD3^{+}CD8^{+}GzmB^{+} cells), Tem cells (CD45^{+}CD3^{+}CD8^{+}CD44^{+}CD62L^{-} cells), Tcm cells (CD45^{+}CD3^{+}CD8^{+}CD44^{+}CD62L^{+} cells), stem-like CD8^{+} T cells (CD45^{+}CD3^{+}CD8^{+}PD-1^{+}TCF-1^{+} cells), terminally exhausted CD8^{+} T cells (CD45^{+}CD3^{+}CD8^{+}PD-1^{+}TCF-1^{-} cells) and terminally exhausted CD8^{+} T cells secreting GzmB (CD45^{+}CD3^{+}CD8^{+}PD-1^{+}TCF-1^{-}GzmB^{+} T cells) in tumors, spleens or lymph nodes of tumor-bearing mice presented on Fig. 3g, 5e-l, 6e-l, 7k-r, 8l and Supplementary Fig. 24a-d, 25a-c, 28e-i, 31f, 37a-d, 38a-d, 43e-l, 44a-f, k and 45a-g, k, l.
