# Supplementary material for: Cell microparticles loaded with tumor antigen and resiquimod reprogram tumor-associated macrophages and promote stem-like CD8+ T cells to boost anti-PD-1 therapy
Source: Nat Commun. 2023 Sep 13;14:5653. doi: 10.1038/s41467-023-41438-9 (PMC10499806; doi:10.1038/s41467-023-41438-9)
Supplement: Supplementary file 2 — Reporting Summary [file 41467_2023_41438_MOESM2_ESM.pdf]

nature portfolio

Corresponding author(s): Lu Gan, Xiangliang Yang

Last updated by author(s): Aug 30, 2023

Reporting Summary

Nature Portfolio wishes to improve the reproducibility of the work that we publish. This form provides structure for consistency and transparency in reporting. For further information on Nature Portfolio policies, see our Editorial Policies and the Editorial Policy Checklist.

Statistics

For all statistical analyses, confirm that the following items are present in the figure legend, table legend, main text, or Methods section.

n/a	Confirmed	
☐	☒	The exact sample size (*n*) for each experimental group/condition, given as a discrete number and unit of measurement
☐	☒	A statement on whether measurements were taken from distinct samples or whether the same sample was measured repeatedly
☐	☒	The statistical test(s) used AND whether they are one- or two-sided<br>*Only common tests should be described solely by name; describe more complex techniques in the Methods section.*
☒	☐	A description of all covariates tested
☒	☐	A description of any assumptions or corrections, such as tests of normality and adjustment for multiple comparisons
☐	☒	A full description of the statistical parameters including central tendency (e.g. means) or other basic estimates (e.g. regression coefficient) AND variation (e.g. standard deviation) or associated estimates of uncertainty (e.g. confidence intervals)
☐	☒	For null hypothesis testing, the test statistic (e.g. *F*, *t*, *r*) with confidence intervals, effect sizes, degrees of freedom and *P* value noted<br>*Give P values as exact values whenever suitable.*
☒	☐	For Bayesian analysis, information on the choice of priors and Markov chain Monte Carlo settings
☒	☐	For hierarchical and complex designs, identification of the appropriate level for tests and full reporting of outcomes
☒	☐	Estimates of effect sizes (e.g. Cohen's *d*, Pearson's *r*), indicating how they were calculated

Our web collection on statistics for biologists contains articles on many of the points above.

Software and code

Policy information about availability of computer code

Data collection	Flow-cytometry data were collected with CytoFLEX S. Images were collected with imaging softwares, such as Caliper IVIS Lumina II for in vivo an ex vivo image, Multi-Mode 8 for AFM images, ELISPOT Plate Reader for IFNγ spot image and Pannoramic MIDI for image of H & E staining.
Data analysis	CytExpert (Ver.2.4.) and Flowjo (Ver.10.5.3) were used to analyse flow-cytometric data; Statistical analysis was performed using GraphPad Prism 7.

For manuscripts utilizing custom algorithms or software that are central to the research but not yet described in published literature, software must be made available to editors and reviewers. We strongly encourage code deposition in a community repository (e.g. GitHub). See the Nature Portfolio guidelines for submitting code & software for further information.

Data

Policy information about availability of data

All manuscripts must include a data availability statement. This statement should provide the following information, where applicable:

- Accession codes, unique identifiers, or web links for publicly available datasets
- A description of any restrictions on data availability
- For clinical datasets or third party data, please ensure that the statement adheres to our policy

The data that support the findings of this study are available within the paper and its Supplementary information files, or are available from the corresponding authors upon reasonable request.

Research involving human participants, their data, or biological material

Policy information about studies with human participants or human data. See also policy information about sex, gender (identity/presentation), and sexual orientation and race, ethnicity and racism.

Reporting on sex and gender	N/A
Reporting on race, ethnicity, or other socially relevant groupings	N/A
Population characteristics	N/A
Recruitment	N/A
Ethics oversight	N/A

Note that full information on the approval of the study protocol must also be provided in the manuscript.

Field-specific reporting

Please select the one below that is the best fit for your research. If you are not sure, read the appropriate sections before making your selection.

[x] Life sciences   [ ] Behavioural & social sciences   [ ] Ecological, evolutionary & environmental sciences

For a reference copy of the document with all sections, see nature.com/documents/nr-reporting-summary-flat.pdf

Life sciences study design

All studies must disclose on these points even when the disclosure is negative.

Sample size	The sample sizes of this study were determined on the basis of similar published studies (Liang, et al. Nat Biomed Eng 3(9): 729-740; Yong, et al. Nat Commun 10(1): 3838; Wei, et al. Nat Commun 12(1):440). In antitumor experiments, 5-10 mice each group were used to evaluate tumor volume; 5-6 mice each group were used to evaluate tumor weight or tumor nodules; 6-10 mice each group were used to analyze survival time of tumor-bearing mice; 5-6 mice each group were used to analyze tumor microenvironment; three mice each group were used to analyze bio-distribution and cellular uptake in vivo; five mice each group were used to analyze AFP-specific CD8+ T cell cytotoxicity against tumor cells in vivo. For other in vitro experiments, the sample size for each group was 3-5.
Data exclusions	No data were excluded in the analysis.
Replication	Experiments were independently repeated as indicated and experimental findings were reproducible (described in figure legends for further details).
Randomization	All samples were randomly allocated into experimental groups.
Blinding	Investigators were blinded during tumor inoculation and animal grouping. Survival data were determined by blinded staff. But investigators were not blinded to group allocation in tumour volume, tumor nodules and tumor weight measurement, because scientists must keep careful track of conditions, it would be exceedingly difficult to blind such studies. For in vitro experiments, blinding was impossible because a single investigator processed all steps of the experiment, and there was no step that introduced personal bias.

Reporting for specific materials, systems and methods

We require information from authors about some types of materials, experimental systems and methods used in many studies. Here, indicate whether each material, system or method listed is relevant to your study. If you are not sure if a list item applies to your research, read the appropriate section before selecting a response.

Materials & experimental systems

n/a	Involved in the study	
[ ]	[x]	Antibodies
[ ]	[x]	Eukaryotic cell lines
[x]	[ ]	Palaeontology and archaeology
[ ]	[x]	Animals and other organisms
[x]	[ ]	Clinical data
[x]	[ ]	Dual use research of concern
[x]	[ ]	Plants

Methods

n/a	Involved in the study	
[x]	[ ]	ChIP-seq
[ ]	[x]	Flow cytometry
[x]	[ ]	MRI-based neuroimaging

Antibodies

Antibodies used

Antibodies used included those for:
(1) Western blot: anti-alpha 1 Fetoprotein (Abcam, cat. No ab213328, clone EPR20667, 1/1000 dilution), anti-Ovalbumin (Abcam, cat. No ab17293, clone 6C8, 1/1000 dilution), anti-β-Actin (CST, cat. No 4970S, clone 13E5, 1/1000 dilution).
(2) Immunofluorescent staining: anti-F4/80 (Sercivebio, cat. No gb113373, 1/3000 dilution), CD11c (Servicebio， cat. No gb11059, 1/200 dilution); anti-TCF-1 (Sercivebio, cat. No gb111378, 1/3000 dilution), Anti-CD86 (CST, cat. No 19589S, clone E5W6H, 1/200 dilution).
(3) Antibodies for anticancer effect study: Anti-CD4 antibody (BioXcell, cat. BE0003-1, clone GK1.5, 100 µ per mouse), Anti-CD8 antibody (BioXcell, cat. BE0061, clone 2.43, 100 µ per mouse), Anti-NK1.1 antibody (BioXcell, cat. BE0036, clone PK136, 100 µ per mouse), Anti-PD-1 antibody (BioXcell, cat. BE0146, clone RMP1-14, 5 mg/kg),
(4) Flow cytometer:
anti-mouse CD11b PerCP/Cyanine5.5 (Biolegend, cat. No 101228, clone M1/70, 1/80 dilution),
anti-mouse CD11b FITC (Biolegend, cat. No 101206, clone M1/70, 1/200 dilution),
anti-mouse F4/80 Brilliant Violet 421TM (Biolegend, cat. No 123137, clone BM8, 1/100 dilution),
anti-mouse F4/80 APC (Biolegend, cat. No 123116, clone BM8, 1/100 dilution),
anti-mouse CD80 PE/Cyanine7 (Biolegend, cat. No 104734, clone 16-10A1, 1/50 dilution),
anti-mouse CD80 PE (Biolegend, cat. No 104708, clone 16-10A1, 1/50 dilution),
anti-mouse CD86 PE/Cyanine7 (Biolegend, cat. No 105116, clone PO3, 1/50 dilution),
anti-mouse I-A/I-E (MHC Ⅱ) APC (Biolegend, cat. No 107613, clone M5/114.15.2, 1/100 dilution),
anti-mouse CD206 APC (Biolegend, cat. No 141708, clone C068C2, 1/50 dilution),
anti-mouse CD206 (MMR) FITC (Biolegend, cat. No 141703, clone C068C2, 1/500 dilution),
anti-mouse H-2Kb bound to SIINFEKL PE (Biolegend, cat. No 141604 , clone 25-D1.16, 1/100 dilution)
anti-mouse CD45 Brilliant Violet 510TM (Biolegend, cat. No 103137, clone 30-F11, 1/20 dilution),
anti-mouse CD45 APC (Biolegend, cat. No 103112, clone 30-F11, 1/100 dilution),
anti-mouse CD45 PerCP/Cyanine5.5 (Biolegend, cat. No 103132, clone 30-F11, 1/80 dilution),
anti-mouse PD-L1 PE (Biolegend, cat. No 124308, clone 10F.9G2, 1/80 dilution),
anti-mouse CD3 PE/Cyanine7 (Biolegend, cat. No 100220, clone 17A2, 1/100 dilution),
anti-mouse CD3 PE (Biolegend, cat. No 100206, clone 17A2, 1/100 dilution),
anti-mouse CD3 PerCP/Cyanine5.5 (Biolegend, cat. No 100218, clone 17A2, 1/20 dilution),
anti-mouse CD3 APC (Biolegend, cat. No 100236, clone 17A2, 1/50 dilution),
anti-mouse CD3 FITC (Biolegend, cat. No 100204, clone 17A2, 1/50 dilution),
anti-mouse CD4 PerCP/Cyanine5.5 (Biolegend, cat. No 100539, clone RM4-5, 1/80 dilution),
anti-mouse CD8a PE/Cyanine7 (Biolegend, cat. No 100722, clone 53-6.7, 1/80 dilution),
anti-mouse CD11c PE/Cyanine7 (Biolegend, cat. No 117317, clone N418, 1/80 dilution),
anti-mouse Ly-6G/Ly-6C (Gr-1) Brilliant Violet 421TM (Biolegend, cat. No 108433, clone RB6-8C5, 1/20 dilution),
anti-mouse Ly-6G/Ly-6C (Gr-1) APC (Biolegend, cat. No 108412, clone RB6-8C5, 1/100 dilution).
anti-mouse CD25 APC (Biolegend, cat. No 102011, clone PC61, 1/100 dilution),
anti-mouse FoxP3 Brilliant Violet 421TM (Biolegend, cat. No 126419, clone MF-14, 1/50 dilution),
anti-mouse IFNγ APC (Biolegend, cat. No 505810, clone XMG1.2, 1/20 dilution),
anti-human/mouse GzmB PE (Biolegend, cat. No 372208, clone QA16A02, 1/20 dilution),
anti-human/mouse GzmB Alexa Fluor® 700 (Biolegend, cat. No 372222, clone QA16A02, 1/20 dilution),
anti-mouse Ki-67 APC (Biolegend, cat. No 652405, clone 16A8, 1/100 dilution),
anti-mouse CD44 FITC (Biolegend, cat. No 103005, clone IM7, 1/200 dilution),
anti-mouse CD62L PE (Biolegend, cat. No 104407, clone MEL-14, 1/100 dilution),
anti-mouse CD279 (PD-1) Brilliant Violet 421TM (Biolegend, cat. No 135221, clone 29F.1A12, 1/200 dilution),
Alexa Fluor® 647 anti-TCF1 (TCF7) antibody (Biolegend, cat. No 655204, clone 7F11A10, 1/20 dilution).
which were used according to manufacturers' instructions.

Validation

All antibodies were verified by the manufacturers and all validation statements can be found on the respective antibody website:
anti-alpha 1 Fetoprotein https://www.abcam.cn/alpha-1-fetoprotein-antibody-epr20667-ab213328.html;
anti-Ovalbumin https://www.abcam.cn/ovalbumin-antibody-6c8-ab17293.html;
anti-β-Actin https://www.cellsignal.cn/products/primary-antibodies/b-actin-13e5-rabbit-mab/4970?site-search-type=Products&N=4294956287&Ntt=4970s&fromPage=plp&_requestid=707340;
anti-F4/80 Rabbit polyclonal antibody https://www.servicebio.cn/goodsdetail?id=6567;
anti-CD11c Rabbit olyclonal antibody https://www.servicebio.com/goodsdetail?id=41340;
anti-TCF-1 https://www.servicebio.cn/goodsdetail?id=3759;
anti-CD86 Rabbit mAb https://www.cellsignal.cn/products/primary-antibodies/cd86-e5w6h-rabbit-mab/19589?site-search-type=Products&N=4294956287&Ntt=19589s&fromPage=plp&_requestid=707732;
anti-mouse/human CD11b PerCP/Cyanine5.5 https://www.biolegend.com/en-us/products/percp-cyanine5-5-anti-mouse-human-cd11b-antibody-4257;
anti-mouse/human CD11b FITC https://www.biolegend.com/en-us/products/fitc-anti-mouse-human-cd11b-antibody-347;
anti-mouse F4/80 Brilliant Violet 421TM https://www.biolegend.com/en-us/products/brilliant-violet-421-anti-mouse-f4-80-antibody-7199;
anti-mouse F4/80 APC https://www.biolegend.com/en-us/products/apc-anti-mouse-f4-80-antibody-4071;
anti-mouse CD80 PE/Cyanine7 https://www.biolegend.com/en-us/products/pe-cyanine7-anti-mouse-cd80-antibody-9320;
anti-mouse CD80 PE https://www.biolegend.com/en-us/products/pe-anti-mouse-cd80-antibody-43;
anti-mouse CD86 PE/Cyanine7 https://www.biolegend.com/en-us/products/pe-cyanine7-anti-mouse-cd86-antibody-3045;

anti-mouse I-A/I-E (MHC Ⅱ) APC https://www.biolegend.com/en-us/products/apc-anti-mouse-i-a-i-e-antibody-2488;
anti-mouse CD206 (MMR) APC https://www.biolegend.com/en-us/products/apc-anti-mouse-cd206-mmr-antibody-7425;
anti-mouse CD206 (MMR) FITC https://www.biolegend.com/en-us/products/fitc-anti-mouse-cd206-mmr-antibody-7318;
anti-mouse H-2Kb bound to SIINFEKL PE https://www.biolegend.com/en-us/products/pe-anti-mouse-h-2kb-bound-to-siinfekl-antibody-7247;
anti-mouse CD45 Brilliant Violet 510TM https://www.biolegend.com/en-us/products/brilliant-violet-510-anti-mouse-cd45-antibody-7995;
anti-mouse CD45 APC https://www.biolegend.com/en-us/products/apc-anti-mouse-cd45-antibody-97;
anti-mouse PD-L1 PE https://www.biolegend.com/en-us/products/pe-anti-mouse-cd274-b7-h1-pd-l1-antibody-4497;
anti-mouse CD45 PerCP/Cyanine5.5 https://www.biolegend.com/en-us/products/percp-cyanine5-5-anti-mouse-cd45-antibody-4264;
anti-mouse CD3 PE/Cyanine7 https://www.biolegend.com/en-us/products/pe-cyanine7-anti-mouse-cd3-antibody-6060;
anti-mouse CD3 PE https://www.biolegend.com/en-us/products/pe-anti-mouse-cd3-antibody-47;
anti-mouse CD3 PerCP/Cyanine5.5 https://www.biolegend.com/en-us/products/percp-cyanine5-5-anti-mouse-cd3-antibody-5596;
anti-mouse CD3 APC https://www.biolegend.com/en-us/products/apc-anti-mouse-cd3-antibody-8055;
anti-mouse CD3 FITC https://www.biolegend.com/en-us/products/fitc-anti-mouse-cd3-antibody-45;
anti-mouse CD4 PerCP/Cyanine5.5 https://www.biolegend.com/en-us/products/percp-cyanine5-5-anti-mouse-cd4-antibody-4230;
anti-mouse CD8a PE/Cyanine7 https://www.biolegend.com/en-us/products/pe-cyanine7-anti-mouse-cd8a-antibody-1906;
anti-mouse CD11c PE/Cyanine7 https://www.biolegend.com/en-us/products/pe-cyanine7-anti-mouse-cd11c-antibody-3086,
anti-mouse Ly-6G/Ly-6C (Gr-1) Brilliant Violet 421TM https://www.biolegend.com/en-us/products/brilliant-violet-421-anti-mouse-ly-6g-ly-6c-gr-1-antibody-7201,
anti-mouse Ly-6G/Ly-6C (Gr-1) APC https://www.biolegend.com/en-us/products/apc-anti-mouse-ly-6g-ly-6c-gr-1-antibody-456.
anti-mouse CD25 APC https://www.biolegend.com/en-us/products/apc-anti-mouse-cd25-antibody-420,
anti-mouse FoxP3 Brilliant Violet 421TM https://www.biolegend.com/en-us/products/brilliant-violet-421-anti-mouse-foxp3-antibody-12143,
anti-mouse IFNγ APC https://www.biolegend.com/en-us/products/apc-anti-mouse-ifn-gamma-antibody-993,
anti-human/mouse GzmB PE https://www.biolegend.com/en-us/products/pe-anti-human-mouse-granzyme-b-recombinant-antibody-14431,
anti-human/mouse GzmB Alexa Fluor® 700 https://www.biolegend.com/en-us/products/alexa-fluor-700-anti-humanmouse-granzyme-b-recombinant-antibody-15595,
anti-mouse Ki-67 APC https://www.biolegend.com/en-us/products/apc-anti-mouse-ki-67-antibody-8447,
anti-mouse CD44 FITC https://www.biolegend.com/en-us/products/fitc-anti-mouse-human-cd44-antibody-314,
anti-mouse CD62L PE https://www.biolegend.com/en-us/products/pe-anti-mouse-cd62l-antibody-386,
anti-mouse CD279 (PD-1) Brilliant Violet 421TM https://www.biolegend.com/en-us/products/brilliant-violet-421-anti-mouse-cd279-pd-1-antibody-7330,
Alexa Fluor® 647 anti-TCF1 (TCF7) antibody https://www.biolegend.com/en-us/products/alexa-fluor-647-anti-tcf1-tcf7-antibody-12714.

Eukaryotic cell lines

Policy information about cell lines and Sex and Gender in Research

Cell line source(s)	H22 and RAW264.7 cells were provided from Type Culture Collection of the Chinese Academy of Sciences (Shanghai, China). B16-OVA cells were kindly provided by Prof. Bo Huang (Institute of Basic Medical Sciences, Chinese Academy of Medical Sciences, Beijing, China). Hepa1-6 cells were purchased from Boster Biological Technology Ltd (Wuhan, China).
Authentication	Each cell line we used was morphologically confirmed according to the information provided by the cell-source center.
Mycoplasma contamination	Cells were tested negative for mycoplasma contamination by using the MycAway-Color one-step mycoplasma detection kit.
Commonly misidentified lines (See ICLAC register)	No commonly misidentified cell lines were used.

Animals and other research organisms

Policy information about studies involving animals; ARRIVE guidelines recommended for reporting animal research, and Sex and Gender in Research

Laboratory animals	C57BL/6 mice (Male or female, 6-8 weeks old), BALB/c mice (male, 6-8 weeks old) and C3H/HeN mice (male, 6-8 weeks old) were purchased from Beijing Vital River Laboratory Animal Technology Co., Ltd. (Beijing, China). Mice were housed in groups of 6 mice per individually ventilated cage in a 12 h light-dark cycle, with constant room temperature 21±1°C and relative humidity 40%-70%. All mice had free access to food and water.
Wild animals	No wild animals were used.
Reporting on sex	Male mice were used in this study for establishing the subcutaneous H22 cancer models, subcutaneous Hepa1-6 cancer models, orthotopic Hepa1-6 cancer models and B16-OVA cancer models; Female C57BL/6 mice and male C3H/HeN mice were crossed-breeding to obtain B6C3 F1 mice and male B6C3 F1 mice were used for establishing DEN-induced HCC models. All cancer models are established based on our historical experience and literature reports. No sex-based analysis were performed and the sex of the animals was not expected to affect the results.

Field-collected samples	No filed-collection was performed.
Ethics oversight	All animal experiments were carried out under the guidance of the Institutional Animal Care and Use Committee at Tongji Medical College, Huazhong University of Science and Technology (Wuhan, China).

Note that full information on the approval of the study protocol must also be provided in the manuscript.

Flow Cytometry

Plots

Confirm that:

☒ The axis labels state the marker and fluorochrome used (e.g. CD4-FITC).

☒ The axis scales are clearly visible. Include numbers along axes only for bottom left plot of group (a 'group' is an analysis of identical markers).

☒ All plots are contour plots with outliers or pseudocolor plots.

☒ A numerical value for number of cells or percentage (with statistics) is provided.

Methodology

Sample preparation	1) Cultured cells or BMDMs were trypsinized, washed with PBS for three time, and then were collected for cytometric analysis;<br>(2) Mouse: For the determination of intracellular accumulation in tumour cells and immune cells and the analysis of tumor immune microenvironment, the tissues were cut into small pieces and incubated in RPMI 1640 medium containing 0.8 mg/mL collagenase type I and 5 µg /ml DNase I at 37 °C for 30 min. The homogenates were passed through a 200-mesh cell strainer, treated with red blood cells lysis buffer and then washed with PBS to acquire single cell suspensions.
Instrument	CytoFLEX S.
Software	The software used for collecting and analyzing was Beckman Coulter cytExpert 2.3.0.84.
Cell population abundance	The relative abundance was maintained by diluting all the samples at equal volume and collecting samples at a fixed and consistent time.
Gating strategy	Cells were gated by FSC/SSC gates and then FSC/FSC-width to select single cells. After that the detail gating strategy was showed as follows:<br>Mouse macrophages: CD11b+, F4/80+,<br>Mouse DCs: CD45+, F4/80-, CD11c+,<br>Mouse M1-like Tumor associated macrophages (TAMs): CD11b+, F4/80+, CD80+ (expression CD86, MHC Ⅱ and H-2Kb bound to SIINFEKL also were examined),<br>Mouse M2-like TAMs: CD11b+, F4/80+, CD206+,<br>Mouse T cells: CD45+, CD3+,<br>Mouse myeloid-derived suppressor cells (MDSCs): CD45+, CD11b+, Gr1+,<br>Mouse regulatory T cells (Tregs): CD45+, CD3+, CD4+, CD25+, FoxP3+,<br>Mouse CD8 T cells: CD45+, CD3+, CD8a+ (Expression of Ki67, CD69, PD-1, IFN-γ and GzmB was examined; And H-2Kb/SIINFEKL tetramer also was staining to affirm the OVA-specific CD8+ T cells),<br>Mouse stem-like CD8 T cells: CD45+, CD3+, CD8a+, TCF-1 (expression of GzmB was examined; AndH-2Kb/SIINFEKL tetramer also was staining to affirm the OVA-specific stem-like CD8+ T cells),<br>Mouse PD-L1+ tumoer cells: CD45-, PDL1+,<br>Mouse effector memory T cells: CD3+, CD8+, CD44+, CD62L-,

☒ Tick this box to confirm that a figure exemplifying the gating strategy is provided in the Supplementary Information.
